# Orthogonal control of expression mean and variance by epigenetic features at different genomic loci

**DOI:** 10.15252/msb.20145704

**Published:** 2015-05-05

**Authors:** Siddharth S Dey, Jonathan E Foley, Prajit Limsirichai, David V Schaffer, Adam P Arkin

**Affiliations:** 1 Department of Chemical and Biomolecular Engineering and the Helen Wills Neuroscience Institute, University of CaliforniaBerkeley, CA, USA; 2 Institute for Quantitative Biosciences, University of CaliforniaBerkeley, CA, USA; 3 Department of Bioengineering, University of CaliforniaBerkeley, CA, USA; 4 Department of Plant and Microbial Biology, University of CaliforniaBerkeley, CA, USA; 5 Physical Biosciences Division, Lawrence Berkeley National LaboratoryBerkeley, CA, USA; 6 Virtual Institute of Microbial Stress and Survival, Lawrence Berkeley National LaboratoryBerkeley, CA, USA; 7 DOE, Joint BioEnergy Institute, Lawrence Berkeley National LaboratoryBerkeley, CA, USA

**Keywords:** chromatin environment, gene expression noise, single-cell biology, single-molecule RNA FISH

## Abstract

While gene expression noise has been shown to drive dramatic phenotypic variations, the molecular basis for this variability in mammalian systems is not well understood. Gene expression has been shown to be regulated by promoter architecture and the associated chromatin environment. However, the exact contribution of these two factors in regulating expression noise has not been explored. Using a dual-reporter lentiviral model system, we deconvolved the influence of the promoter sequence to systematically study the contribution of the chromatin environment at different genomic locations in regulating expression noise. By integrating a large-scale analysis to quantify mRNA levels by smFISH and protein levels by flow cytometry in single cells, we found that mean expression and noise are uncorrelated across genomic locations. Furthermore, we showed that this independence could be explained by the orthogonal control of mean expression by the transcript burst size and noise by the burst frequency. Finally, we showed that genomic locations displaying higher expression noise are associated with more repressed chromatin, thereby indicating the contribution of the chromatin environment in regulating expression noise.

See also: **S Tyagi** (May 2015)

## Introduction

While there is increasing evidence that non-genetic individuality in cells arises in part from noise in the fundamental processes of gene expression, there have been few studies elucidating either the mechanistic basis of this expression noise from a given gene or its coupling to the immediate genetic environment. Here, we take the first steps toward understanding the molecular basis of gene expression noise. It has long been known that isogenic populations grown under identical conditions exhibit non-genetic heterogeneity (Spudich & Koshland, 1976), which arises from the inherently random nature of biochemical processes in which infrequent reactions and/or low numbers of molecules regulate important behavior in a system. Such random fluctuations or noise can be further amplified by underlying biological networks to drive dramatic phenotypic variations within isogenic populations of bacterial, yeast, insect, and mammalian cells (Arkin *et al*, 1998; Balaban *et al*, 2004; Weinberger *et al*, 2005; Wernet *et al*, 2006; Acar *et al*, 2008; Chang *et al*, 2008; Spencer *et al*, 2009; Balázsi *et al*, 2011). In particular, gene expression noise arising from stochastic fluctuations in transcription has been shown to be an important source of non-genetic heterogeneity in mammalian cells. Over the last decade, significant work has modeled and experimentally validated this process of stochastic gene expression, enabled by the development of powerful single-cell analysis techniques such as flow cytometry, high-throughput microscopy, and recently single-molecule RNA fluorescent *in situ* hybridization (smFISH) (Thattai & van Oudenaarden, 2001; Elowitz *et al*, 2002; Blake *et al*, 2003; Paulsson, 2004; Raser & O'Shea, 2004; Kaern *et al*, 2005; Friedman *et al*, 2006; Raj *et al*, 2006; Raj & van Oudenaarden, 2008). However, the mechanistic roles and impact of key molecular factors on gene expression noise remain largely undissected, which is the focus of this work.

Transcriptional noise in the expression of a gene is modulated by both the genomic location with its associated chromatin environment, and its promoter architecture. Elegant large-scale studies in *S. cerevisiae* have investigated how noise scales with mean protein expression for several endogenous genes (Bar-Even *et al*, 2006); however, the contributions of the promoter architecture and genomic location to gene expression noise were not explored. Additionally, a study utilizing the well-characterized yeast Pho5 promoter identified the contribution of different transcription factor mutants, which remodel nucleosomes during transcriptional activation, to expression noise (Mao *et al*, 2010). However, it was not clear whether the results reflected the general dynamics of gene expression at different sites across the whole genome or were specific to the particular endogenous locus examined. Also, while a number of transcriptional regulatory mechanisms are conserved between yeast and mammals, mammalian genomes exhibit additional complexity, with differences in both large-scale chromatin dynamics and promoter proximal chromatin marks that may limit the generalization of findings from yeast to mammals (Rando & Chang, 2009; Court *et al*, 2011). A recent study in mammalian cells investigated the impact of *cis*-regulatory sequences on gene expression noise (Suter *et al*, 2011); however, the use of pharmacological perturbations that globally alter chromatin structure, as well as the small dataset, did not enable a general understanding of the role of the genomic location in regulating gene expression noise. Thus, our understanding of how both genomic locations and underlying molecular factors regulate gene expression noise is limited.

To address how genomic locations regulate expression noise, we designed a lentiviral-based system that effectively deconvolves the influence of the promoter sequence from genomic location, thereby allowing a comprehensive and systematic study of how the genomic environment influences gene expression noise regulation in a mammalian system. In particular, semi-random lentiviral integration efficiently samples a myriad of genomic locations while maintaining the same promoter architecture (Jordan *et al*, 2001). Furthermore, lentiviral promoters exhibit many archetypal features of endogenous eukaryotic promoters, such as a TATA box, extensive *cis* acting elements, and well-positioned nucleosomes along the promoter (Verdin *et al*, 1993; Pereira *et al*, 2000). Therefore, to systematically study the influence of genomic location on mRNA and protein expression noise, we generated a large set of single-cell clones spanning hundreds of integration positions. Furthermore, to address the question of which key molecular players may be involved in regulating expression noise, we extended recent studies in *S*. *cerevisiae* (Mao *et al*, 2010; Weinberger *et al*, 2012)—which have begun to unravel the roles of chromatin and chromatin-modifying complexes—to a mammalian system by systematically measuring the chromatin state of promoters integrated into different genomic locations.

While we and others have previously used lentiviral-based vectors to study gene expression noise (Weinberger *et al*, 2005, 2008; Singh *et al*, 2010a; Skupsky *et al*, 2010; Dar *et al*, 2012), small datasets and indirect measurements of transcription have resulted in discrepancies in the results. Furthermore, these studies have been unable to infer specific molecular features underlying the regulation of expression noise. We previously found that the transcript burst size—the number of transcripts generated during a short interval during which a “bursty” promoter is mediating transcription—primarily correlates with the mean level of gene expression, whereas another study recently found that both the burst size and frequency—the rate of promoter transitions into the productive bursty state—correlate with mean expression across genomic locations (Skupsky *et al*, 2010; Dar *et al*, 2012). To discriminate between these conflicting results, we conducted a large-scale analysis to accurately quantify mRNA levels by smFISH as well as protein levels by flow cytometry. We then integrated these data to show orthogonal control of mean expression by the burst size and noise by the burst frequency. Furthermore, to gain deeper molecular insights, we assessed chromatin accessibility across integration sites and found that noisier clones with infrequent bursts are associated with more repressed chromatin. Identifying the molecular players and sources of such non-genetic heterogeneity may offer better means to target diseases such as viral pathogenesis or cancer (Balaban *et al*, 2004; Weinberger *et al*, 2005; Cohen *et al*, 2008; Spencer *et al*, 2009; Sharma *et al*, 2010; Singh *et al*, 2010b; Miller-Jensen *et al*, 2011).

## Results

### High-throughput generation of single-integration clones to comprehensively observe diversity in expression mean and expression noise

**Figure 1:**
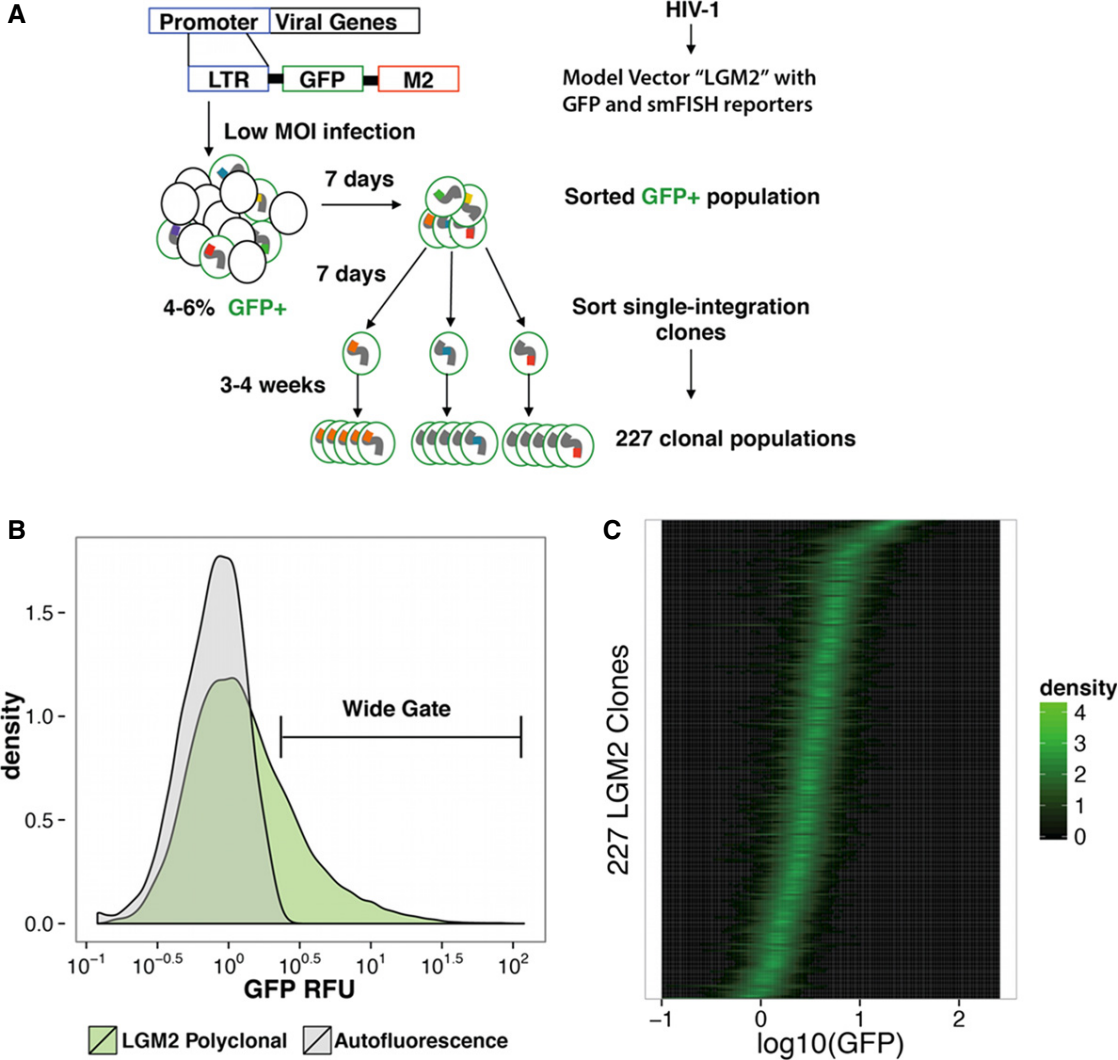
High-throughput single-integration clone generation robustly captures the diversity of expression mean and noise exhibited by HIV-1 across integration sites

To facilitate a comprehensive, systematic analysis of the modulation of long terminal repeat (LTR) basal transcriptional dynamics across genomic locations, a high-throughput workflow (Fig1A) was designed to create a diverse and representative set of clones. To directly compare RNA and protein levels in single cells within such clonal populations, and thereby provide a deeper and more direct inference of the transcriptional dynamics than previously studied, a model vector (LGM2) consisting of the HIV-1 LTR driving dual protein (GFP) and RNA (M2 smFISH) reporters was developed. Importantly, previous studies have used limited sets of fewer than 40 clones (Singh *et al*, 2010a; Skupsky *et al*, 2010) or used a short half-life GFP variant (d2GFP; Dar *et al*, 2012) that, due to the accompanying reduced fluorescence and sensitivity, limited analysis to only the subset of genomic locations that yield high gene expression. Furthermore, our and others’ studies have strictly used GFP reporters of gene expression activity as opposed to direct quantification of mRNA molecules. Together, these previously used features may provide a biased or limited portrait of expression dynamics across genomic locations.

Clonal generation workflow. Jurkat T cells were infected at low MOI (˜0.05) with HIV-1 LGM2 and allowed to reach steady state expression for 7 days. To facilitate discrimination of infected cells by GFP fluorescence, the culture was stimulated with TNF-α for 16 h. Following stimulation, GFP-positive cells were sorted and allowed to expand for 7 days. From this GFP^;^ population, single cells were sorted into 96-well plates and allowed to expand for 3–4 weeks to generate 227 clonal populations.

Unbiased clone sorting from long tailed polyclonal distribution. As indicated by the overlay of the polyclonal LGM2 GFP distribution with autofluorescence of uninfected Jurkat T cells, the HIV-1 LTR supports wide and highly skewed gene expression with cells exhibiting expression 1–2 orders of magnitude greater than the very low expression mode. For further analysis of the modulation of LTR gene expression across viral integrations, an unbiased gate distinct from autofluorescence was used to capture a diverse and representative set of single-cell clones.

Evidence of noise independence from mean expression across large set of clones. Analysis of GFP expression of the 227 clones by flow cytometry reveals modulation of both the mean level of expression and expression ‘noise’ as indicated by significant differences in distribution width within groups of clones with similar means. Each row within the plot represents the 1D kernel smoothed density of the GFP expression of a single clone, with the clones rank ordered by mean expression.

To capture a spectrum of single-integration clones that robustly represent the wide and highly skewed bulk distribution (Fig1B), single cells were sorted into 96-well plates from a wide gate spanning 1.8 log_10_ RFU units, distinct from autofluorescence. This gate, chosen to limit sampling bias toward any particular regime of mean expression level, resulted in 227 LGM2 clones. While we isolate clones from the entire range of mean expressions, preferential integration of HIV into actively transcribing genes and activating chromatin marks potentially results in more clones being integrated within such regions than within gene deserts, methylated CpG regions, and repressive chromatin marks (Schröder, 2002; Lewinski *et al*, 2005; Wang *et al*, 2007). Furthermore, to validate results obtained from this first large set, we repeated the sorting scheme to isolate an additional, independent 191 LGM2 single-integration clones (Supplementary Fig S6). We observed a high degree of population level agreement between the two sets of clones, suggesting that any potential effects arising from experimental bias were negligible.

The expression of each of the initial 227 clones was determined by flow cytometry, and each distribution was subsequently gated (Supplementary Fig S1) with a data-driven algorithm that samples a small region of forward scatter and side scatter space and retains at least 4,000 cells per clone. However, we found minimal dependence between the gate chosen and the resulting scaling of the GFP distribution moment (Supplementary Figs S2, S3, and S4). Importantly, this ensemble of clones exhibited mean expression levels spanning 2 orders of magnitude, identical to the range observed in the bulk distribution (Fig1C).

### Uncorrelated expression mean and noise suggest primarily orthogonal control across genomic locations

For a given mean expression level, there was considerable variability in distribution width (Fig1C). If these distributions arose from a constant-rate Poisson transcription process in which the promoter is always in a productive state, we would expect distribution variance to scale linearly with mean and the coefficient of variation (CV) to decrease as CV∝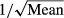. In contrast, for a non-Poissonian process in which RNA is produced in infrequent bursts arising from stochastic promoter transitions from a ‘Off’ state and an ‘On’ state, variance would be anticipated to relate to mean through a power-law relationship. We used the GFP distribution moments from the 227 clones to differentiate between these scenarios.

**Figure 2:**
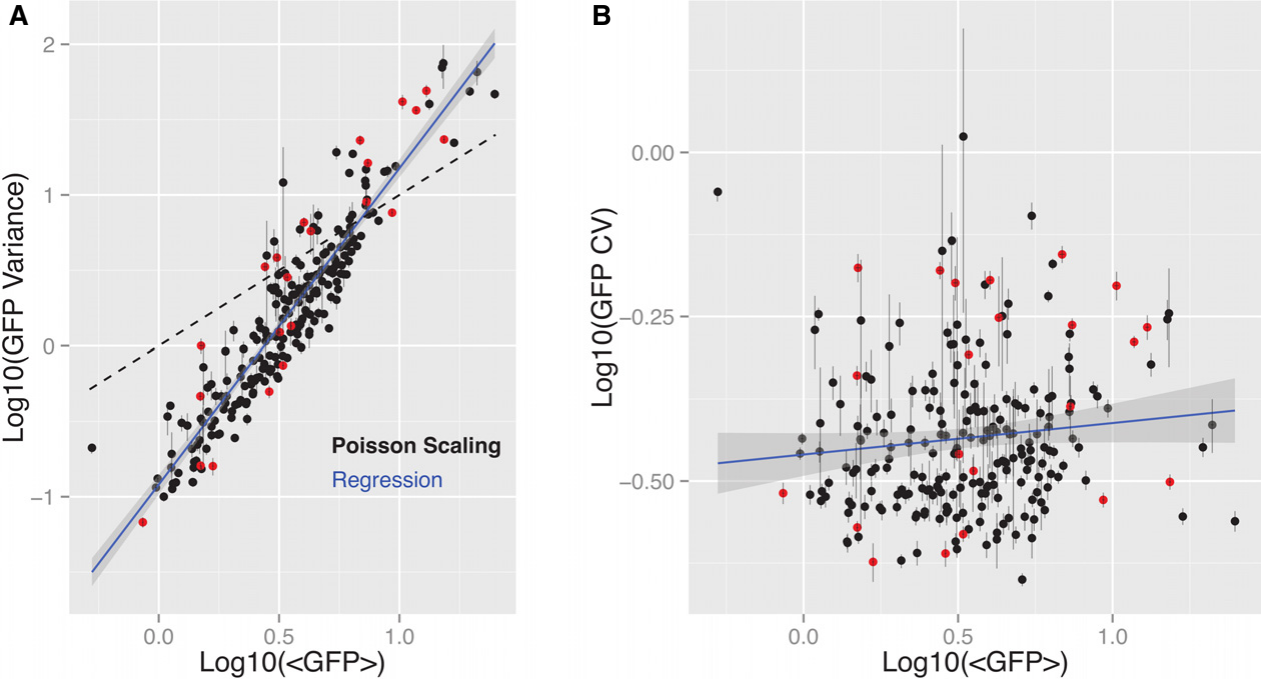
Scaling between distribution mean and variance is highly consistent with non-Poissonian transcription while CV scaling suggests independent control of expression mean and noise across integration positions

To infer whether either of these basic models of gene expression could explain the observed distributions, we next examined the relationships between the mean and variance in gene expression for the clonal populations, and between their expression mean and the coefficient of variation (expression noise). Examination of the relationship (Fig2A, blue line) between GFP mean and GFP variance revealed that in log-transformed space, variance is highly correlated with distribution mean (*R*^2^ = 0.86, Spearman correlation coefficient, *r*_s_ = 0.92), strongly suggesting an underlying power-law relationship with σ^2^∝μ^2.1 ± 0.1^. This scaling is distinct from Poisson scaling (Fig2A, dashed black), which would predict a linear relationship resulting from a constant rate of production. However, this power-law relationship is consistent (*P* < 0.01) with distribution scaling arising through promoter transitions between Off and On states.

Clonal distribution moment scaling. GFP fluorescence of approximately 10^4^ cells from each clone was measured via flow cytometry. The highly significant power-law relationship between distribution mean and variance with a log-log linear regression slope of 2.1 ± 0.11 (blue line, *R*^2^ = 0.86, *r*_s_ = 0.92, *P* < 0.001) is distinct from Poisson scaling (dashed line) and is consistent with distribution scaling arising through ‘input-controlled’ promoter transitions between “Off” and “On” states.

Expression mean and noise independence. Uncorrelated GFP expression noise, measured as log-log-transformed coefficient of variation (CV), and GFP mean across clones (*R*^2^ = 0.013, *r*_s_ = 0.12) suggests independent control of mean expression and expression noise across integration positions. Error bars represent 95% confidence interval estimates derived from bootstrapped GFP fluorescence distributions for each clone. Red dots represent clones on which single-molecule mRNA FISH was performed.

While correlations between the first two moments is a useful comparison to the theoretical models, analysis of a dimensionless expression noise normalized with respect to mean, such as the coefficient of variation (CV, σ/μ), provides a direct comparison to other experimental systems (Elowitz *et al*, 2002; Bar-Even *et al*, 2006; Singh *et al*, 2010a). Consistent with an underlying non-Poissonian process, examination of the relationship (Fig2B, blue line) between GFP CV and GFP mean reveals an uncorrelated relationship (*R*^2^ = 0.013, *r*_s_ = 0.12). Interestingly, for a given mean level of expression, a nearly constant range of CV is sampled (Fig2B and Supplementary Fig S5). This result stands in stark contrast to genome-wide studies of expression noise in yeast (Bar-Even *et al*, 2006; Newman *et al*, 2006), which have indicated that ∼CV∝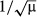. We found that this lack of correlation was not an artifact of the gating strategy used (Supplementary Fig S4). Furthermore, to ensure that these results were not influenced by artifacts introduced during infections, cell culture, or cell sorting, the second set of 191 isolated single-integration clones (as described in Fig1A) reproduced a lack of correlation between mean expression and CV (Supplementary Fig S6). Additionally, to ensure that the lack of correlation between mean expression and CV was not influenced by extrinsic sources of noise such as cell size, we noted that forward scatter measurements in flow cytometry were not correlated with GFP fluorescence (Supplementary Fig S2). Similarly, applying a data-driven gating strategy that sampled a small region of forward and side scatter space showed no linear or other monotonic correlation between cell size and GFP expression (Supplementary Fig S3). Furthermore, by choosing either a wide gate of forward and side scatter of live cells or a small gate centered on the density mode of forward and side scatter, we found that the observed independence of gene expression noise and mean expression did not change (Supplementary Fig S4A). Moreover, by changing the gate size centered around the density mode to capture between 10–90% of the cells, we found the slope of the variance in gene expression vs. mean expression best-fit line did not change (Supplementary Fig S4C). Finally, we previously performed extensive controls in our experimental system to show that gene expression noise in our system was dominated by intrinsic noise and was not influenced by extrinsic sources of noise such as cell cycle, cell size, or aneuploidy (Weinberger *et al*, 2005). Similarly, studies in other mammalian systems have used two-color reporter systems to show that gene expression noise is dominated by intrinsic sources (Raj *et al*, 2006). Taken together, these factors strongly suggest that across genomic locations, expression mean and expression noise are differentially controlled by intrinsic processes rather than extrinsic sources of noise (see Supplementary Information for additional details).

### Subsetting clones for further analysis by smFISH reflects properties of the full set of clones

**Figure 3:**
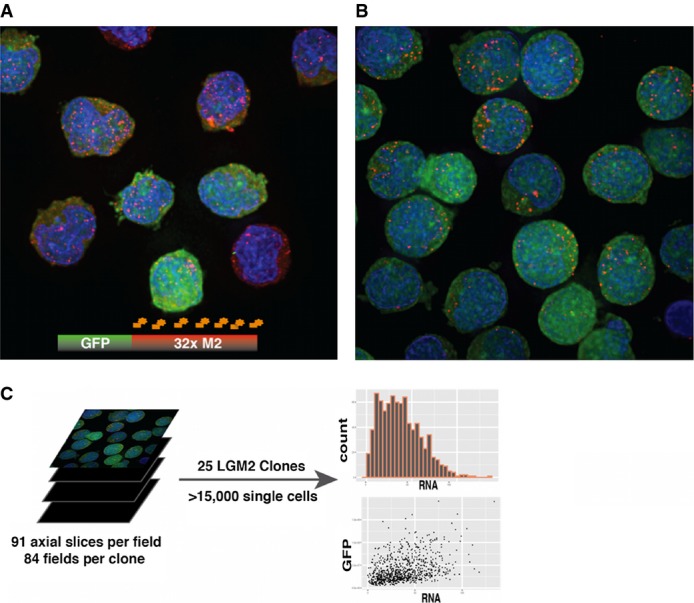
Hybrid unsupervised segmentation of both cells and smFISH signals enables high-throughput analysis of expression output of thousands of single cells

The above results provide an initial, diagnostic assessment that is strongly consistent with a non-Poissonian bursting transcriptional mechanism. However, the observations are inherently dependent on post-transcriptional processes that may obscure important underlying information. In particular, the long half-life of the GFP reporter potentially buffers and smoothens important dynamic information, yet use of a destabilized, lower sensitivity GFP would preclude analysis of all but the brightest clones. Therefore, to provide a more direct and sensitive measure of transcriptional mechanisms underlying the observed differential variation of expression mean and expression noise across genomic locations, a subset of 25 clones was selected for analysis by smFISH using a probe against the M2 array in the integrated LGM2 provirus. These clones were chosen by allocating the initial 227 distributions into four clusters via a hierarchical clustering approach (Supplementary Information and Supplementary Figs S7 and S8). To effectively sample both the range of expression means and noises observed in this full set of clones, and to enable detailed inferences about the molecular factors regulating gene expression dynamics as a function of the genomic location, pairs of clones with similar means but markedly different CVs were selected from each cluster. Examination of the relationship between mean and variance (Supplementary Fig S9A), and mean and CV (Supplementary Fig S9B), for this subset of clones revealed trends that are not statistically different (*F*-test, *P* > 0.1) from those observed in the full set of clones, indicating that the sample was representative. We thus proceeded to quantify exact RNA copy numbers per cell in both high noise (Fig3A) and low noise (Fig3B) clones from this subset.

A, B Transcriptional bursting and expression heterogeneity in high and low clones. (A) Significant heterogeneity in RNA copy number and GFP within a high noise clonal population and direct evidence of transcriptional bursting is revealed by a false-colored maximum intensity projection (MIP) of a deconvolved wide-field optically sectioned field of cells imaged at 100× magnification in three channels (GFP, DAPI, TAMRA). (B) Same as (A), with image from a low noise clonal population.

C High-throughput image analysis of microscopy images. Custom morphological segmentation of cells in fields and FISH signals enabled analysis of over 15,000 cells across 25 clones. For each clone, a distribution of RNA number per cell is determined (see Supplementary Information for details).

### RNA distribution shape is highly related to protein distribution shape

**Figure 4:**
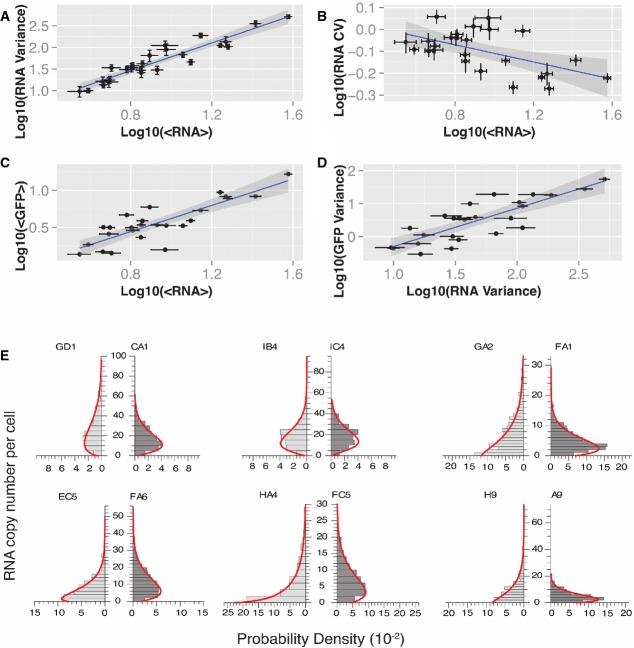
RNA distribution scaling and systematic fitting of RNA distributions reveal that LTR expression output is primarily controlled by intrinsic fluctuations in promoter activity

High-throughput computational image analysis resolved counts of LGM2 RNA per cell for an average of 630 cells per clone (Fig3C, Supplementary Information and Supplementary Figs S10, S11, and S12). To analyze whether the process was stereotypical Poisson vs. non-Poissonian at the transcriptional level, we determined the relationships for RNA variance or RNA CV as a function of RNA mean (Fig4A, blue line and B, blue line, respectively). We find that RNA mean and variance are highly correlated (*R*^2^ = 0.89, *r*_s_ = 0.93) with σ_RNA_^2^∝μ_RNA_^1.68 ± 0.25^. This relationship again follows a power law, though the different slope from the protein result (Fig2A), indicating that post-transcriptional steps may augment noise. However, we again find that RNA CV is only weakly dependent on RNA mean (*R*^2^ = 0.31, *r*_s_ = −0.38, *P* = 0.03), suggestive of a non-Poissonian process (Fig4B). This lack of correlation between CV and mean at the RNA level further suggests that the observed orthogonal control of expression mean and noise is not governed by extrinsic sources related to translation, such as the number of ribosomes.

A RNA variance correlated with mean RNA copy number. Log-log linear regression of RNA variance as a function of RNA mean finds a high-confidence scaling with a slope of 1.68 ± 0.25 (*R*^2^ = 0.89, *r*_s_ = 0.92, *P* < 0.001), which is intermediate to Poisson and ‘input-controlled’ scaling.

B RNA mean and noise primarily independent. Log-log linear regression of RNA CV (noise) as a function of mean finds an approximately uncorrelated of noise on mean expression (slope = −0.2 ± 0.13, *R*^2^ = 0.31, *r*_s_ = −0.38, *P* = 0.03). This suggests that RNA mean and expression noise are controlled by primarily independent mechanisms.

C, D RNA distribution shape predominantly explains GFP distribution shape. RNA and GFP means are highly correlated (C) (*R*^2^ = 0.79, *r*_s_ = 0.86, *P* < 0.001). Similarly, RNA and GFP variances are strongly correlated (D) (*R*^2^ = 0.73, *r*_s_ = 0.84, *P* < 0.001), with RNA variance explaining the majority of variation observed in GFP variance. Together, these suggest that contributions to the mean and width of the protein distributions from processes downstream of transcription are minimal. Furthermore, this strongly implies that intrinsic transcriptional dynamics are the primary determinant of protein distribution shape.

E Maximum-likelihood fitting of a two-state model to RNA distributions. Copy number distributions were determined by smFISH and automated analysis of the set of 25 selected LGM2 clones. Resulting distributions were fit to the analytical probability density function (*pdf*) of a stochastic two-state transcriptional model through maximum-likelihood parameter estimation. mRNA copy number distributions are depicted as paired density histograms for six representative pairs of high noise (pair left, light gray) and low noise (pair right, dark gray). Each pair represents clones with similar mean expression but different CV. Experimentally determined histograms are shown overlaid with the probability density functions (red curve) evaluated using best-fit model parameters for each clone.

Data information: In (A–D), error bars on RNA moments represent 95% confidence interval estimates derived from bootstrapped smFISH distributions for each clone. *r*_s_ represents the Spearman correlation coefficient for the explanatory and response variables in each pairwise regression, and *P*-values represent support for correlation.

Furthermore, it is frequently assumed that translation is a constant-rate first-order process that does not vary between clonal populations. Under such an assumption, we would expect RNA mean and variance to strongly predict protein mean and variance. To examine this assumption directly, RNA mean and variance of distributions for each clone were compared to their corresponding GFP moments. We find that variation in RNA mean predicts variations in GFP mean well (*R*^2^ = 0.79, *P* < 0.001) (Fig4C), and RNA variance predominantly explains GFP variance (*R*^2^ = 0.73, *P* < 0.001) (Fig4D). This suggests that GFP distribution shape is predominantly determined by the underlying RNA distribution, with contributions from post-transcriptional processes.

### Systematic fitting of RNA distributions reveals that a two-state model can describe both low and high noise clones

While scaling of the RNA distributions is highly suggestive of an underlying non-Poissonian process, with distribution shape predominantly determined by stochastic promoter transitions between ‘On’ and ‘Off’ states, further analysis may yield insights into the unanticipated independence of mean expression level and noise. This may imply that distinct molecular mechanisms could regulate the mean and noise of gene expression, thereby allowing cells to precisely tune gene expression distributions. We therefore examined kinetic parameters underlying the gene expression distribution shapes. Specifically, maximum-likelihood estimation (MLE) of kinetic parameters was performed for the ‘standard’ two-state transcription model (Supplementary Figs S13 and S14), which has received considerable attention by ourselves and other recent studies (Raser & O'Shea, 2004; Raj *et al*, 2006; Skupsky *et al*, 2010). While this model is an idealization of complex molecular phenomena, it has an analytical solution (Peccoud & Ycart, 1995; Raj *et al*, 2006) and has been found to parsimoniously explain observed protein and RNA distributions for synthetic and endogenous promoters in yeast and mammalian systems (Raser & O'Shea, 2004; Raj *et al*, 2006; Skupsky *et al*, 2010).

MLE best-fit parameters for all 25 clones analyzed with smFISH quantitatively fit the measured mRNA histograms (Fig4E). Specifically, we find that each clone can be described by the average rate of promoter transitions to the ‘On’ state and the average number of transcripts produced in the ‘On’ state. In particular, we find that the two-state model can effectively fit both the low (Fig4E right/dark gray in each pair) and high noise (Fig4E left/light gray in each pair) clone pairs with similar mean expression levels previously selected for smFISH analysis. Consistent with prior work (Singh *et al*, 2010a; Skupsky *et al*, 2010), burst size (the ratio of transcription rate to promoter ‘Off’ rate) and frequency (the inverse of promoter ‘On’ rate) vary across genomic locations. Furthermore, we find that all fitted clones are non-Poissonian with inferred burst sizes larger than 1. Finally, systematic fitting of the RNA distributions obtained from smFISH for clones with integration positions over the entire genome was the first step toward understanding the molecular features that may explain the observed protein and mRNA distribution shape scaling.

### Differential control of expression mean and noise by burst size and rate of promoter ON transitions

**Figure 5:**
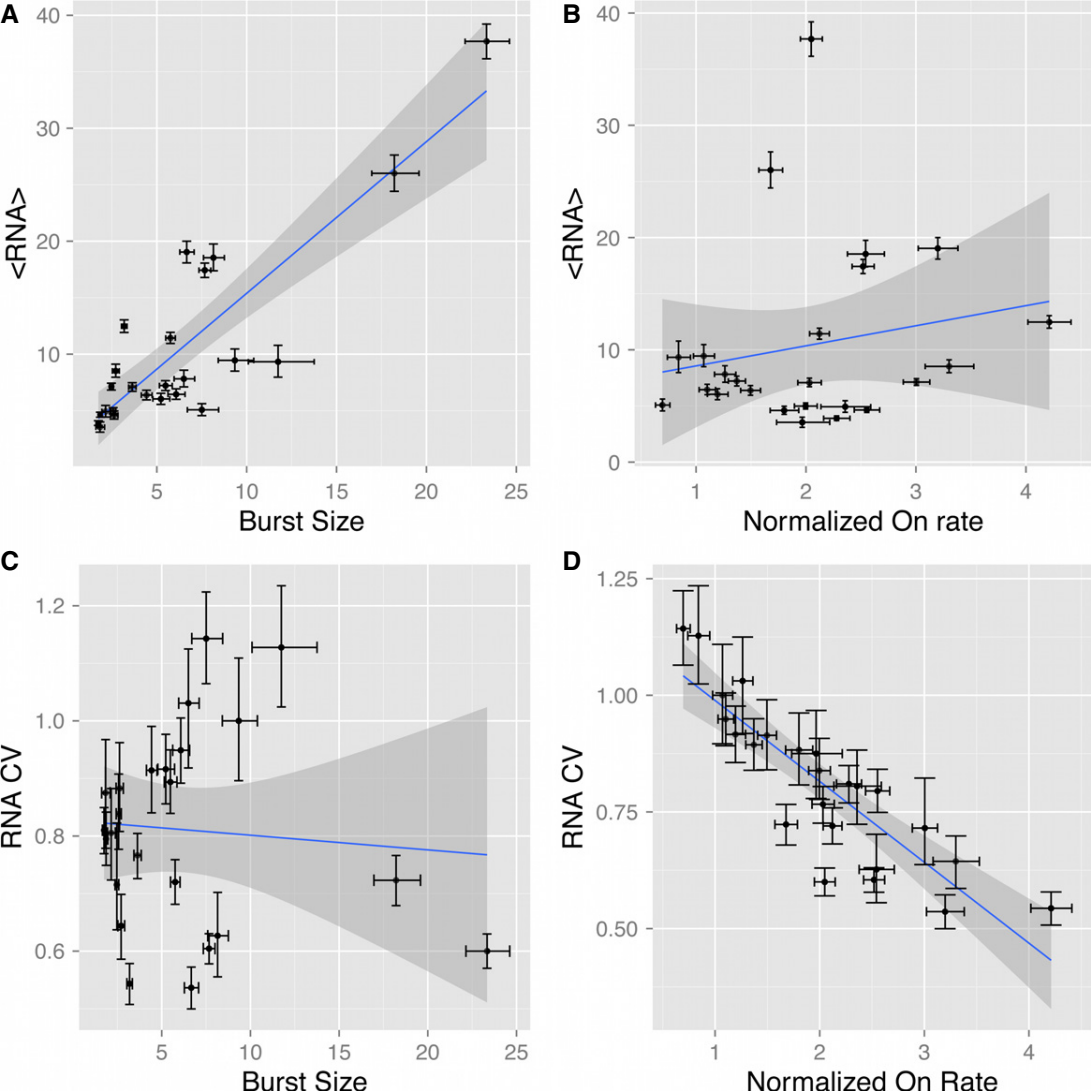
Maximum-likelihood fitting of a stochastic model of transcription to smFISH distributions reveals burst size primarily accounts for expression mean while promoter transitions from Off to On primarily accounts for expression noise

Systematic determination of RNA distributions with smFISH and fitting to a stochastic model of transcription revealed several interesting correlations. Burst size is primarily correlated with RNA mean (Fig5A, *R*^2^ = 0.75, *r*_s_ = 0.8), whereas the promoter ‘On’ rate (normalized by the measured rate of RNA degradation) explained expression noise (Fig5D, *R*^2^ = 0.75, *r*_s_ = −0.89). This suggested that burst size and promoter On rate may differentially regulate mean expression and noise and may thus have distinct molecular underpinnings. To further analyze these relationships, we examined correlations between burst frequency and RNA mean (Fig5B) and between burst size and RNA noise (Fig5C). Surprisingly, we found that neither is correlated significantly (*P* > 0.3). This result strongly suggests that while burst size and burst frequency vary across genomic locations, they independently determine the mean and noise of expression (Supplementary Fig S15). Specifically, increased burst sizes drive higher mean RNA expression, while an increasing rate of promoter transitions to the ‘On’ state reduces expression noise in a highly monotonic relationship. While the kinetic linkage between promoter On rate and expression noise has been theorized, this is to our knowledge the first experimental demonstration that the rate of promoter transitions to the ‘On’ state is inversely correlated with the level of transcriptional noise.

Burst size accounts for variation in mean expression. Maximum-likelihood estimation of burst size for each clone significantly accounts for a majority of the variation observed in mean RNA copy number across integration positions (slope = 1.35 ± 0.35, *R*^2^ = 0.75, *r*_s_ = 0.8, *P* < 0.001).

Promoter On transitions are uncorrelated from expression mean. Maximum-likelihood estimation of promoter On rate normalized by the rate RNA degradation cannot account for variation in mean RNA copy number (*R*^2^ = 0.05, *r*_s_ = 0.2, *P* = 0.37).

Burst size is uncorrelated from expression noise. Burst size cannot account for the variation in observed expression noise (CV) (*R*^2^ = 0.01, *r*_s_ = 0.01).

Promoter On transitions accounts for expression noise variation. Normalized On rate (by rate of RNA degradation) significantly accounts for variation in expression noise (CV) across integration positions (slope = −0.52 ± 0.14, *R*^2^ = 0.75, *r*_s_ = −0.89, *P* < 0.001). This independence in cross-correlations suggests that while both burst size and promoter On rate vary across integration positions, expression mean and noise are controlled by primarily orthogonal mechanisms.

Data information: Error bars on RNA moments represent 95% confidence interval estimates derived from bootstrapped smFISH distributions for each clone. Error bars on model parameters represent 95% confidence intervals estimated using 1.92 log-likelihood ratio units. *r*_s_ represents the Spearman correlation coefficient for the explanatory and response variables in each pairwise regression and *P*-values represent support for correlation.

Our finding of the independent variation and kinetic control of expression mean and noise across genomic locations contrasts with previous findings (Dar *et al*, 2012). This may be a consequence of the previous use of clones with a bias toward high expression (burst sizes ∼100–200) or a lack of systematic parameter estimation. Regardless, our results significantly enhance our mechanistic understanding by providing a concise kinetic mechanism that can account for the observed orthogonality between mean and expression noise initially observed in both GFP and RNA moment scaling (Figs2 and 4). However, what mechanism may differentially affect burst size and On rate remains to be discovered. The relationship between promoter On rate and expression noise led us to question whether stochastic molecular events such as chromatin or nucleosome dynamics might control the promoter On rate and expression noise.

### Nucleosome occupancy at the transcription start site regulates gene expression noise and burst frequency

**Figure 6:**
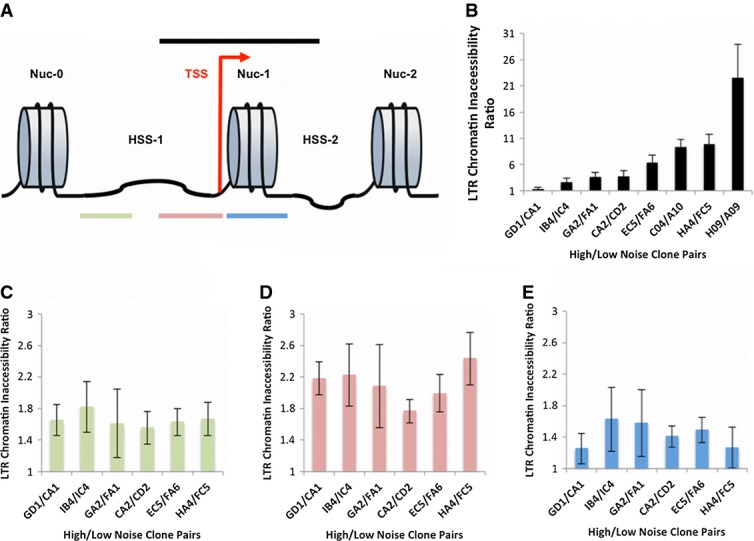
Noisier clones exhibit more inaccessible promoters

HIV-1 has well-positioned nucleosomes along the entire length of the viral genome (Verdin *et al*, 1993; Rafati *et al*, 2011). In particular, the promoter has one nucleosome (called Nuc-1) that is immediately adjacent to the transcriptional start site (TSS) and another that is 450 nucleotides upstream (called Nuc-0) (Fig6A). Such well-positioned nucleosomes, stereotypical of TATA-containing promoters in yeast and mammalian cells, are in a strong position to regulate gene expression dynamics. We thus hypothesized that the nucleosome organization at the LTR and the chromatin density at the site of integration may play a critical role in influencing transitions between the Off and On promoter states, thereby regulating the width of the RNA/protein distributions. Since HIV-1 integrations sample a large diversity of genomic locations (Jordan *et al*, 2001), our model system offers further mechanistic insights into how chromosome location may be important in regulating expression noise of endogenous genes.

A HIV-1 LTR exhibits positioned nucleosomes. Schematic figure showing the well-characterized placement of nucleosomes along the HIV-1 promoter. Nuc-1 is positioned at the transcription start site (TSS), and Nuc-0 is placed further upstream along the promoter.

B Significant differences in DNA accessibility around TSS between high and low noise clones. The region highlighted in black was probed after digestion with DNase I for 16 clones. The figure plots the level of chromatin inaccessibility for pairs of clones that exhibit similar mean levels of expression. Clones that exhibit noisier gene expression also have more closed chromatin.

C–E Differential DNA accessibility between high and low noise clones across three distinct sites in the LTR. The HIV-1 promoter was probed in greater detail with the color scheme matching that shown in (A). As in (B), it was found that for similar mean levels of gene expression, noisier clones exhibited more closed chromatin along the entire length of the promoter (ratios > 1 in all cases). Interestingly, compared to the two other regions of the promoter, the hypersensitive site (HSS) showed maximum differences in chromatin inaccessibility between high and low noise clones.

Data information: qPCR experiments were performed in triplicate, and the error bars reflect the standard deviation from the mean.

To quantitatively measure the chromatin accessibility of the LTR across different genomic locations, we used DNase I sensitivity assays as previously described (Dey *et al*, 2012; Miller-Jensen *et al*, 2012). Initially, to assess whether the chromatin accessibility at the promoter may be related to gene expression noise in general, we probed a large region of the promoter centered on the TSS (as indicated by the black bar, Fig6A and B) for 6 pairs of clones (Fig4E), with each pair expressing similar mean levels of RNA/protein but different expression noise. In agreement with our hypothesis that the chromatin density at the promoter may regulate expression noise characteristics, we found that the ratio of chromatin inaccessibility between high and low noise clone pairs of the *same* mean was > 1 in all cases, implying that clones that are integrated into more closed chromatin display noisier gene expression (Fig6B). Thus, it appeared that chromatin features within the promoter potentially regulate gene expression noise, independent of the mean level of expression.

To gain a more detailed molecular picture of chromatin features regulating gene expression noise, we performed DNase I sensitivity analysis of 3 shorter regions (Rafati *et al*, 2011) along the length of the viral promoter: (i) Nuc-1, a nucleosome that has previously been shown to be important for LTR-mediated gene expression, (ii) the hypersensitive site (HSS) between Nuc-1 and Nuc-0, a region that contains binding sites for critical transcription factors NF-κB and Sp1, and (iii) Nuc-0. Recently, it was shown that the presence of a nucleosome at Nuc-1 or HSS could potentially significantly influence the gene expression state of the viral promoter (Rafati *et al*, 2011). Analysis of the chromatin state at these three sites showed that the ratio of chromatin inaccessibility for the high noise clone to its low noise partner is always > 1, further supporting our hypothesis that high noise clones have more closed chromatin (Fig6C–E) along the entire length of the promoter.

Furthermore, while the ratios are > 1 at all three sites, the difference in the chromatin state between high and low noise clones is maximized at the HSS (Fig6D). This high ratio at the HSS could arise from more inaccessible chromatin for the high noise clones or more open chromatin for the low noise clones at the HSS. We found that while the high noise clones appear to have consistently high and similar levels of chromatin inaccessibility at all three sites along the promoter, the HSS site for the low noise clones has significantly lower levels of chromatin inaccessibility than the other two sites (Nuc-1 and Nuc-0) in the promoter (Supplementary Fig S16). Thus, low noise clones appear to have particularly open chromatin at HSS, which might arise from the binding of transcription factors such as NF-κB and Sp1 to sites within this region (Burnett *et al*, 2009). Thus, these results show that for a given mean level of gene expression, noise in expression (of RNA/protein distribution) is correlated with integration site-specific chromatin accessibility at the promoter.

**Figure 7:**
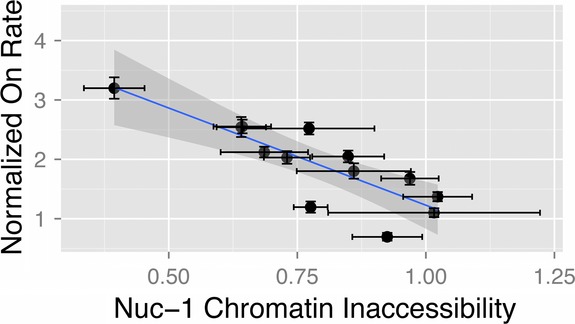
Nuc-1 occupancy can explain variation in promoter activation rates across genomic positions Chromatin state around the transcription start site is strongly correlated with the promoter activation rate. Unlike other regions of the HIV-1 promoter (see Supplementary Fig S17), chromatin inaccessibility around Nuc-1 was strongly correlated with the frequency of promoter transition from the Off to On state (slope = −1.1 ± 0.5, *R*^2^ = 0.69, *r*_s_ = −0.85, *P* < 0.001). Clones with more closed chromatin produced more infrequent transitions from the Off to On promoter state. qPCR experiments were performed in triplicate, and error bars reflect the standard deviation from the mean. Error bars on ka represent 95% confidence intervals estimated using 1.92 log-likelihood ratio units. *r*_s_ represents the Spearman correlation coefficient for the explanatory and response variables in each pairwise regression, and *P*-values represent support for correlation.

We next sought to quantify whether chromatin accessibility at the promoter correlates with the primary metrics describing ‘bursty’ transcription – the transcript burst size and the rate of promoter transitions from the Off to On state. Interestingly, we found that the rate of promoter On transitions correlates very strongly with the chromatin density around the transcription start site at Nuc-1 (*R*^2^ = 0.69, *r*_s_ = −0.85, *P* < 0.001) (Fig7). This relationship was much weaker in the case of the HSS (*R*^2^ = 0.49) or Nuc-0 (*R*^2^ = 0.41) site (Supplementary Fig S17). Finally, to understand whether some linear combination of the chromatin density around Nuc-1, HSS, and Nuc-0 is a better predictor of the rate of promoter On transitions, we performed principal components analysis (PCA), which established that the rate of promoter transitions is nearly inversely correlated with the chromatin density at Nuc-1 and essentially orthogonal to the HSS and Nuc-0 axis (Supplementary Fig S18A). Thus, the chromatin state at Nuc-1 is the best predictor of the burst frequency, with increased chromatin density at the transcription start site resulting in more infrequent transitions to the On promoter state and greater expression noise. In contrast, the burst size appears to be uncorrelated with the chromatin state of the promoter (Supplementary Fig S18B).

These studies demonstrate that the chromatin environment around the LTR at different genomic locations, especially the nucleosome Nuc-1 proximal to the transcription start site, correlates strongly with the rate of promoter transitions to the On state, and the CV (expression noise) of the RNA/protein distributions (Supplementary Fig S19). To our knowledge, these findings provide the first indication of molecular features that determine gene expression noise across different genomic locations in a mammalian system.

## Discussion

Quantitative investigation of the flow of genetic information from dynamic chromatin regulation to transcription and translation is fundamental to our understanding of genome function and cellular behavior. While the nature of noisy stochastic gene expression has received intense interest in both synthetic and natural systems (Arkin *et al*, 1998; Thattai & van Oudenaarden, 2001; Elowitz *et al*, 2002; Golding *et al*, 2005; Weinberger *et al*, 2005; Raj *et al*, 2006), the molecular mechanisms underlying noisy expression phenotypes have remained elusive. Here, using a dual-reporter lentiviral model system, we analyzed over 400 clonal populations by flow cytometry and over 15,000 single cells from 25 clonal populations by smFISH. By linking clonal protein expression, single-cell measurements of mRNA copy number, and measurements of promoter chromatin occupancy, we provided three key insights. First, expression mean and CV are uncorrelated across integration positions. Second, this observed large-scale independence between expression mean and CV can be systematically explained by the independent control of gene expression mean by burst size and CV by promoter On rate. Lastly, chromatin density at the promoter can explain the promoter activation rate but does not provide an explanation for burst size. In particular, we systematically demonstrate that promoter chromatin density correlates with promoter activation rate, which in turn regulates the CV or noise of the RNA distribution. This suggests that local chromatin density may modulate the frequency of transcriptional bursting and thereby tune expression noise.

These findings contrast with recent studies suggesting equal modulation of burst size and frequency with expression mean across genomic sites (Dar *et al*, 2012). The equal modulation of burst size and frequency observed by Dar *et al* was inferred from polyclonal populations that were assumed to correspond to single-cell clones to model underlying gene expression kinetic parameters. Also, the clustered polyclonal populations were treated as isogenic populations based on the *qualitative* similarity of their CV values. Therefore, it is unclear how the clustering procedure may obscure the estimation of kinetic parameters across genomic locations. Furthermore, reported burst sizes (> 100 for most clones) lie at the high end of the full range of LTR-driven expression (Skupsky *et al*, 2010). As a result, conclusions on the role of genomic location on noise characteristics are limited.

In addition, our results contrast with the assertion that *cis*-regulatory elements but not chromatin structure influence expression noise and bursting dynamics (Suter *et al*, 2011). However, this conclusion rests on very limited clonal data gathered using trichostatin A, a highly cytotoxic histone deacetylase inhibitor that *globally* remodels chromatin and has unknown molecular effects on the specific genomic locations studied. Furthermore, their dataset shows that a few clones exhibit large changes in transcription rates and burst sizes upon stimulation with TSA, suggesting that chromatin can influence bursting kinetics, thereby making the claims of Suter *et al* inconclusive. The conclusions of another recent study may similarly be affected by the use of the strong, global inhibitors TSA and 5-AzaC (Viñuelas *et al*, 2013). While differences in results between these studies could arise from some of the reasons mentioned above, the use of different model organisms, cell types, promoters, and chromosomal locations could also influence the final conclusions of these studies. Taken together, these recent studies and our work suggest that both promoter architecture and the local chromatin environment may combine to yield the observed expression distributions and inferred transcription dynamics. Further studies are needed to systematically study the nature of this coupling, which may influence the evolution of promoters and selection for non-random positioning of genes within the genomes to minimize noise (Batada & Hurst, 2007).

We also observed differential nucleosome occupancy across genomic locations and quantitatively correlated chromatin density with the rate of promoter activation. Our observation of differential chromatin density across genomic locations is supported by genome-wide studies in *Drosophila* that quantified nucleosome dynamics and showed that different regions of the genome could have variable nucleosome turnover rates (Deal *et al*, 2010). Together, these findings may suggest that variable nucleosome occupancy is a general mechanism for regulating promoter switching rates and noise. Furthermore, we provide quantitative evidence that the chromatin density around the TSS plays the most important role in regulating the promoter On rate. Several biochemical processes could result in the independent control of expression mean and noise observed in this study. While we show that the nucleosome positioned at the transcription start site plays the most important role in regulating gene expression noise, variability in chromatin modifications and transcription factor recruitment at different genomic locations may influence polymerase processivity or burst size, thereby independently regulating mean gene expression. Finally, the interplay between these processes, together with other mechanisms of gene regulation such as DNA methylation or polymerase pausing, could potentially result in the independent control of expression mean and noise. Further investigation of the molecular underpinnings of burst size modulation will further deepen our molecular interpretation of bursting dynamics.

Nucleosomes have been known to regulate transcription by setting a threshold for initiating transcription (Lam *et al*, 2008; Miller-Jensen *et al*, 2012). This work shows that in mammalian systems, nucleosomes and chromatin density around the TSS may also be important to fine-tune transcription (Tirosh & Barkai, 2008; Hornung *et al*, 2012). Such a strategy where the chromatin environment regulates gene expression noise could be an important mechanism to generate different cellular phenotypes from isogenic populations in a manner that can confer increased evolutionary fitness. For example, in simple eukaryotes, such phenotypic switching has been shown to confer increased survival fitness (Acar *et al*, 2008). Similarly, such cell-to-cell heterogeneity in cancer populations may be an important mechanism that contributes to different drug sensitivities and drug-tolerant states (Cohen *et al*, 2008; Spencer *et al*, 2009; Sharma *et al*, 2010; Singh *et al*, 2010b). Finally, phenotypic heterogeneity may be an important contributor to producing low-frequency latent HIV-1 infections that remain one of the main obstacles to completely eliminating the virus from a patient (Weinberger *et al*, 2005; Ho *et al*, 2013). Thus, understanding the contribution of the chromatin environment in regulating gene expression noise and the resulting phenotypic heterogeneity may be important for understanding the design principles governing evolution and for developing better treatments in a variety of diseased states. Just as miRNAs have been implicated in imparting robustness to genetic and environmental perturbations (Ebert & Sharp, 2012), nucleosomes may perform a similar function. Therefore, this study quantitatively establishes that nucleosomes and chromatin density around the TSS, in addition to its known functions in controlling expression levels and imparting cellular memory, may regulate gene expression noise.

## Materials and Methods

### Viral cloning

To facilitate single-molecule detection of transcripts, the M2 array from pGEM-M2-32x (Raj *et al*, 2006) was cloned as a directional SalI-XhoI fragment into the single XhoI site of HIV CLG (Weinberger *et al*, 2005) to generate HIV CLGM2 used in this study.

### Cell culture

Jurkat cells, used for creating clonal LGM2 cell line and HEK293T cells, used for packaging virus were cultured in RPMI 1640 and Iscove's DMEM, respectively. Cells were maintained at 37°C and 5% CO_2_ with the cell media supplemented with 10% fetal bovine serum (FBS) and 100 U/ml of penicillin–streptomycin.

### Viral harvesting, titering, and infections

To package the LGM2 construct, HEK293T cells were transfected with 10 μg of the plasmid along with the helper plasmids pMDLg/pRRE, pcDNA3 IVS VSV-G, and pRSV-Rev as described previously and harvested (Dull *et al*, 1998). Harvested lentivirus was concentrated by ultracentrifugation to yield between 10^7^ and 10^8^ infectious units/ml. To titer, 10^5^ Jurkat cells per well were infected with a range of concentrated virus doses, and 6 days post-infection, gene expression was stimulated with 20 ng/ml tumor necrosis factor-α (TNF-α, Sigma-Aldrich). After stimulation for 18 h, GFP expression was measured by flow cytometry, and titering curves were constructed by determining the percentages of cells that exhibited GFP fluorescence greater than background levels.

### Clone generation

Assuming a Poisson distribution, the well with 5% GFP-infected cells was selected for expansion. This corresponds with a low MOI of ∼0.05 and as previously demonstrated ensures at most one integration event per infected cell within the population (Weinberger *et al*, 2005). The selected population was expanded for 7 days and stimulated with TNF-α as described above. Approximately 10^5^ cells were sorted from the GFP^;^ population on a DAKO-Cytomation MoFlo Sorter. The resulting population, which represents a polyclonal ensemble of single-integration clones, was expanded for 7 days. Single-cell clones were sorted from a wide gate distinct from background fluorescence into multi-well plates and cultured for 3–4 weeks to facilitate expansion.

### Flow cytometry

GFP expression of expanded clonal cultures was assessed by flow cytometry using a Beckman Coulter FC500 analytical cytometer. Multiple reads over the course of a week were obtained to ensure that measured fluorescence represented stationary distributions of gene expression. Flow cytometry data were processed using Bioconductor packages in custom R scripts as described in the Supplementary Information.

### Cell fixation

For each sample, 1–2 million cells washed once in PBS and allowed to adhere to 0.01 mg/ml poly-L-lysine (Sigma-Aldrich P5899)-coated chambered #1 cover glass (Nunc Lab-Tek #155380). Cells were fixed with 4% formaldehyde (Sigma-Aldrich 252549) solution in PBS. Slides with fixed cells were washed once with PBS and 70% cold ethanol added to permeabalize cellular membranes. Slides were stored at 4°C until hybridization.

### *In situ* hybridization

Single-molecule labeling of LGM2 transcripts was performed as previously described with the following exceptions (Raj *et al*, 2006): (a) 35% formamide was used in the hybridization buffer, (b) a shorter, TAMRA dual end labeled probe (BioSearch Inc., 5′-GTCGATCAGCTGGCTGGTGCTCTTCGTCCACAAAC-3′) was used, and c) the hybridization reaction was carried out for 16 h at 30°C. Prior to imaging, slides were washed twice with 2× SSC 35% formamide. Cell nuclei were counterstained with 0.5 μg/ml DAPI. Just prior to imaging, samples were mounted under a coverslip with aqueous oxygen scavenging buffer system (Raj *et al*, 2008).

### Imaging and deconvolution

84 randomly selected fields were imaged per sample. To facilitate imaging of the entire cell volume, each field was imaged in three channels (GFP, DAPI, TAMRA) 90 *z*-axial slices at 0.2-μm spacing with a 100× oil immersion objective on a Deltavision Core (API) widefield deconvolution microscope equipped with standard fluorescent filters. Image stacks were iteratively deconvolved using an experimentally determined point-spread function (PSF) with Huygens Core 3.3 (SVI) running on a small Linux cluster. Optimal deconvolution parameters were empirically determined to yield the best subjective image quality. Custom Tcl scripts running under Silicon Grid Engine were used to manage job creation and scheduling.

### Image processing

Deconvolved image stacks were processed using custom software developed in MATLAB (Mathworks Inc.) using the DIPImage Toolbox (Quantitative Imaging Group, TU Delft). Under minimal user intervention, the software automatically segments cells and smFISH objects within single cells. A detailed protocol for the processing pipeline is contained in the Supplementary Information.

### Model fitting

RNA distributions were fit using maximum-likelihood estimation (MLE) of model parameters using the full analytical solution to the two-state stochastic gene expression model (Peccoud & Ycart, 1995). MLE was implemented using custom code in Mathematica 8 (Wolfram Inc.) as numerical minimization over the negative log-likelihood function defined over the *pdf* given the observed RNA counts the rate of RNA degradation set to our experimentally determined rate and transcription rate assumed to be constant across integration sites as previously discussed (Skupsky *et al*, 2010). In this manner, the effective fit parameters are the burst frequency and burst size.

### Statistical analysis

95% confidence intervals on descriptive statistics of RNA distributions were estimated from the 2.5% and 97.5% quantiles of bootstrapped copy number counts per cell. 95% confidence intervals on fit parameters were estimated from the log-likelihood function assuming asymptotic normality of the estimates. These analyses were performed in Mathematica 8. All regression and correlation analysis was performed in R using the *lm* and *rcorr* functions. The regression *P*-values of all primary inferences reported in this study fall below an α of 0.05. Distribution clustering and principal component analysis were performed in MATLAB (Mathworks Inc).

### DNase I sensitivity assay

The assay was performed using the EpiQ Chromatin Analysis Kit (Bio-Rad) as previously described (Dey *et al*, 2012; Miller-Jensen *et al*, 2012). Briefly, 2.5 × 10^5^ cells were either treated with DNase I or left untreated for 1 h at 37°C. After quenching the reaction, DNA was extracted and quantified by qPCR using the EpiQ Chromatin SYBR Supermix (Bio-Rad). The following primers were used to quantify the chromatin density at the HIV promoter:

LTRfor (5′-GGACTTTCCGCTGGGGACTTTCCAGGG-3′)

LTRrev (5′-GCGCGCTTCAGCAAGCCGAGTCCTGCGTCGAG-3′)

Nuc-1for (5′-AGCTCTCTGGCTAACTAGGG-3′)

Nuc-1rev (5′-AAAGGGTCTGAGGGATCTCTAG-3′)

HSSfor (5′-GGGACTTTCCGCTGGGGAC-3′)

HSSrev (5′-CCCAGTACAGGCAAAAAGCAGC-3′)

Nuc-0for (5′-GAGCCTGCATGGGATGG-3′)

Nuc-0rev (5′-CTCCGGATGCAGCTCTC-3′)

The qPCR results were normalized by the chromatin density at the hemoglobin promoter using the primers:

hHBBfor (5′-AAGCCAGTGCCAGAAGAGCCAAGGA-3′)

hHBBrev (5′-CCCACAGGGCAGTAACGGCAGACTT-3′)

qPCR experiments were performed in triplicate with melt curves to ensure product specificity.

### mRNA extraction and RT–qPCR

To determine the half-life of transcripts, a polyclonal LGM2 population was stimulated by α-amanitin and total RNA was extracted from cells at different time points using TRIzol (Invitrogen). RNA was also extracted from unstimulated cells at these time points. LGM2 and β-actin mRNA were quantified by RT–qPCR using the single-step Quantitect SYBR Green RT–PCR kit (Qiagen). qPCR experiments were performed in triplicate. For additional details and primers, see Supplementary Information.

## Supporting Information

Supplementary Information

Analysis Scripts

Review Process File
