# Supplementary material for: Orthogonal control of expression mean and variance by epigenetic features at different genomic loci
Source: Mol Syst Biol. 2015 May 5;11(5):806. doi: 10.15252/msb.20145704 (PMC4461400; doi:10.15252/msb.20145704)
Supplement: Supplementary file 1 [file msb0011-0806-sd1.pdf]

Supplementary Information for

Orthogonal control of expression mean and variance by epigenetic features at different genomic loci

Siddharth S. Dey[1,2,†,||], Jonathan E. Foley[3,||], Prajit Limsirichai[4], David V. Schaffer[1,2,3,5,*], Adam P. Arkin[3,5,6,7,*]

[1] Department of Chemical and Biomolecular Engineering and the Helen Wills Neuroscience Institute, University of California, Berkeley, CA 94720, USA
[2] Institute for Quantitative Biosciences, University of California, Berkeley, CA 94720, USA
[3] Department of Bioengineering, University of California, Berkeley, CA 94720, USA
[4] Department of Plant and Microbial Biology, University of California, Berkeley, CA 94720, USA
[5] Physical Biosciences Division, Lawrence Berkeley National Laboratory, Berkeley, CA 94720, USA
[6] Virtual Institute of Microbial Stress and Survival, Lawrence Berkeley National Laboratory, Berkeley, CA 94720
[7] DOE, Joint BioEnergy Institute, Lawrence Berkeley National Laboratory, Berkeley, CA 94720

* Corresponding authors.

[||] These authors contributed equally to this work

[†] Present address: Hubrecht Institute-KNAW (Royal Netherlands Academy of Arts and Sciences) and University Medical Center Utrecht, Cancer Genomics Netherlands, 3584 CT Utrecht, The Netherlands.

Table of Contents

I. Gating of Flow Cytometry Data

II. Independent Clone Generation Replicate

III. Clone Selection and Stability

IV. Computational Analysis of Microscopy Images

V. Measurement of RNA Degradation

VI. Model Fitting

VII. Additional Fit Parameter Analysis

VIII. Additional Nucleosome Sensitivity Analysis

IX. Summary of Statistical Analysis

X. Experimentally Measured Protein and mRNA Moments and Model Fit Parameters

I. Gating of Flow Cytometry Data

Previous studies in yeast have suggested a strong dependence between cell size (Forward Scatter, FSC) and noise as measured by the Coefficient of Variation (CV) or CV^2(Newman *et al*, 2006). As previously suggested (Skupsky *et al*, 2010), we find an extremely narrow gate to be unnecessary to limit the correlation between cell size (FSC) and GFP. However, to minimize any errors due to manual gating we used a data driven gating strategy provided by the *norm2h* filter of the Bioconductor flowViz package in R (Supplementary Figure S1). To ascertain whether there was any residual correlation between FSC and GFP we examined both linear (Supplementary Figure S2) and non-linear correlations and found that for all clones there is no significant correlation between FSC and GFP (Supplementary Figure S3).

Furthermore, as previously suggested we do not find that size gating has any quantitative effect on the scaling between GFP mean and GFP variance or between GFP mean and GFP CV (Newman *et al*, 2006). Specifically, we examined the relationship between expression noise (CV) as a function of expression before and after gating and found that both exhibited uncorrelated relationships. (Supplementary Figure S4A) Additionally, we examined whether the relationship between variance and mean was strongly modulated by gate size (Supplementary Figure S4B). We found that neither the regression slope (Supplementary Figure S4C) nor the extent of correlation (Supplementary Figure S4D) was strongly affected by gate size. In contrast to findings in yeast, these results suggest that cell size does not have a significant impact on either expression mean or noise.

In addition to cell size, we have previously shown that other extrinsic sources of noise, such as cell cycle and cell line aneuploidy do not influence gene expression noise in our system (Weinberger *et al*, 2005). Further, the lack of correlation between mean expression and CV at the mRNA level also suggests that extrinsic cellular mechanisms involved with translation, such as the number of ribosomes, do not influence gene expression noise in our system (Figure 4B). Finally, we make a theoretical argument that has previously been discussed in detail, for why other extrinsic factors, such as transcription factors, potentially do not influence the results of our study (Skupsky *et al*, 2010). First, the range of noise (CV) observed with the HIV promoter for different clones

used in this study (~0.25-0.8) is larger than that observed in other large-scale studies involving several cellular promoters (~0.1-0.3) (Sigal *et al*, 2006; Newman & Weissman, 2006). Next, the noise observed in the mRNA distributions (CV_t) are typically higher than those observed in the protein (GFP) distributions (CV_p) (Figures 2B and 5C). Further, assuming a bursting regime, the noise in promoter state transitions can be given by (Skupsky *et al*, 2010):

$$CV_{promoter}^2 = \left(\frac{b}{b+1}\right)\left(\frac{k_r}{k_{t-}}\right)CV_t^2$$

In our dataset, we observe burst sizes (*b)* greater than 1 (Figure 5A), and thus the first term in the equation is close to 1. The second term is much greater than 1 for all the clones. This implies that the noise introduced from the promoter state transitions ($CV_{promoter}$) is greater than the noise in transcript counts (CV_t), which in turn is greater than noise in protein distributions (CV_p). In general, in a cascade of stochastic processes, for noise at a particular step to make a significant contribution and propagate to the next step, the upstream step needs to be noisier than the downstream step. But since it appears that the observed noise in protein (GFP) distributions from the HIV promoter is possibly higher than endogenous genes, it is unlikely that noise in transcription factors (or other cellular proteins) could influence and propagate through the HIV promoter to significantly affect the observed mRNA and GFP distributions.

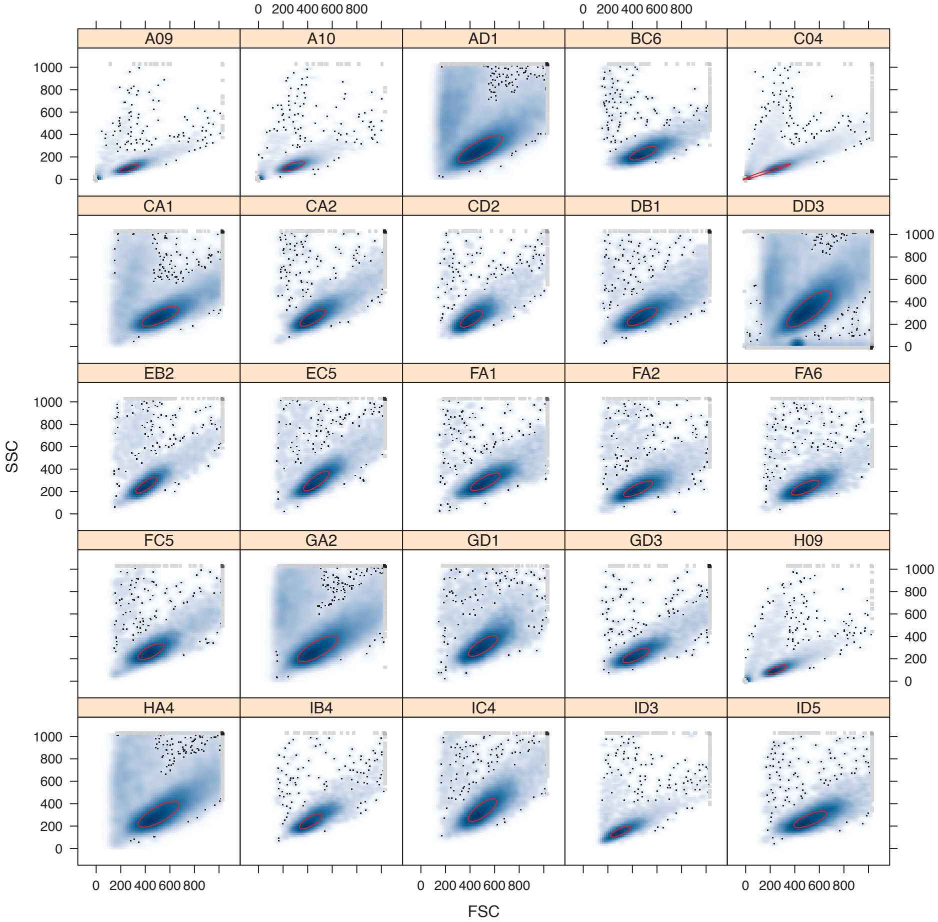

Supplementary Figure 1 - Representation of gating strategy for subset of clones. Raw flow cytometry channel data for all 227 LGM2 clones was gated in Side Scatter (SSC) and Forward Scatter (FSC) space using the *norm2h* filter provided by the BioCondutor flowViz package. This filter fits a 2D Gaussian around the mode of the data, which results in a small ellipse. The resulting gate for the 25 clones used for FISH analysis is depicted (red ellipse) overlaid on a smoothed FSC v SSC scatterplot.

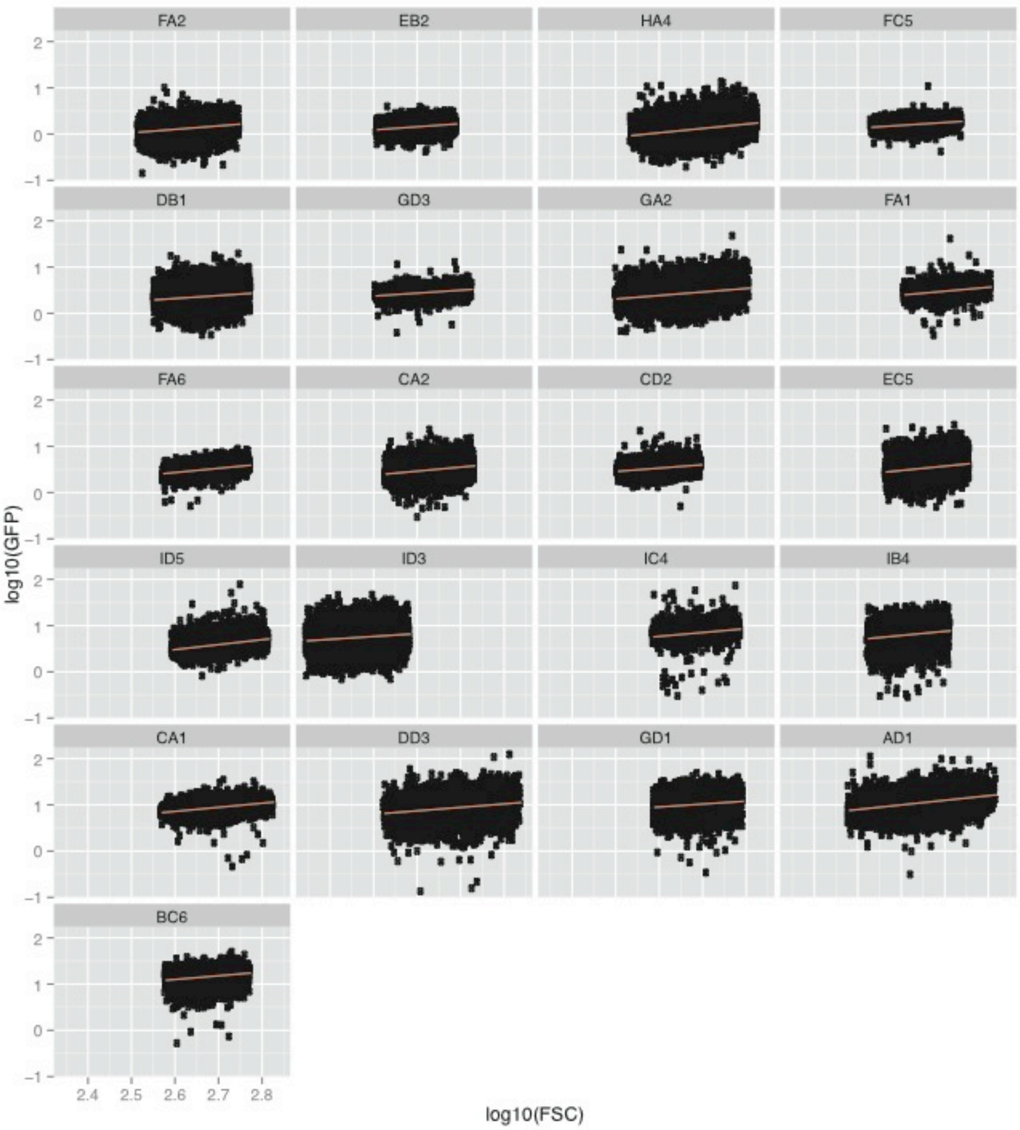

Supplementary Figure 2 - Cell Size and GFP are not linearly correlated post-sorting. As an initial assessment of whether any relationship remained between cell size (FSC) and GFP we examined scatterplots and performed linear fits (red line) for the clones used for FISH analysis. There is no apparent relationship and all linear regressions do not have slopes significantly different from zero. Furthermore, the scatterplots suggests very little difference (the range of GFP is independent of FSC) in the CV in GFP as a function of FSC.

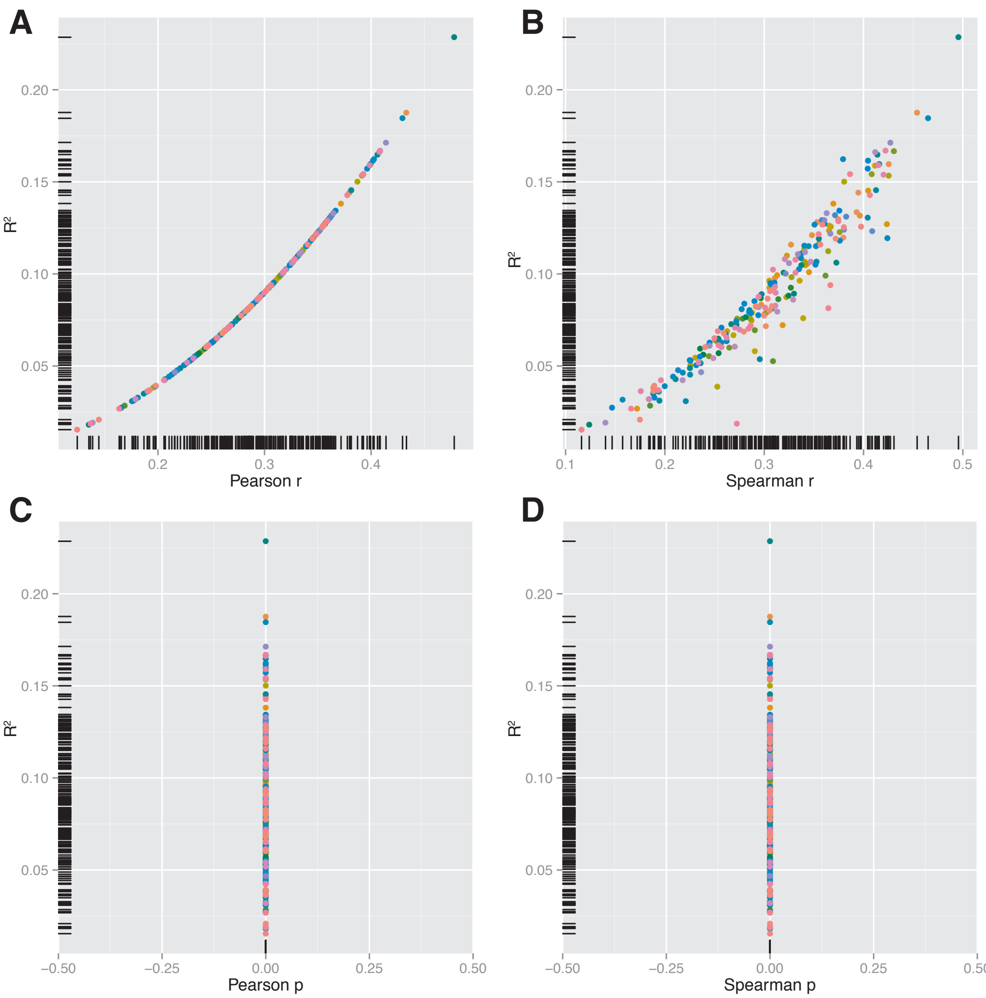

Supplementary Figure 3 - No significant linear or non-linear relationship between cell size and GFP expression observed across all clones. To further verify our gating strategy and analyze whether cell size correlated to GFP expression across all 227 LGM2 clones we examined both linear and non-linear correlations and performed statistical testing. (A) Minimal variance in GFP explained by FSC. A plot of FSC vs. GFP R^2 versus the Pearson correlation coefficient for each clone reveals very little observed variance (R^2<0.2,Pearson r<0.4). The marginal rug plots indicate the relative density for the majority of clones centers around R^2~0.1 and Pearson r~0.3. (B) Minimal non-parametric relationship between FSC and GFP. To further address whether any monotonic relationship between FSC and GFP existed post-gating, we generated a plot of FSC vs. GFP R^2 versus the Spearman correlation coefficient. Similar to linear correlation, this plot suggests very limited monotonic relationships exist for all the clones

(Spearman $r<0.5$). (C,D) Minimal correlations not significantly different from zero. Statistical testing of the correlations does not find significant support for either linear or non-linear correlations.

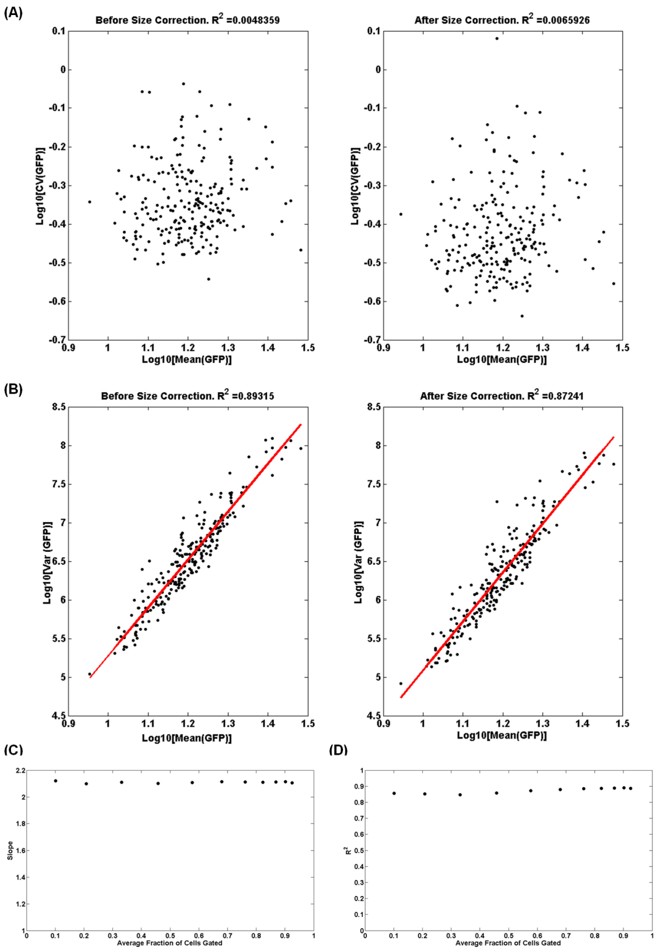

Supplementary Figure 4 - Cell size does not significantly underlie moment based relationships. (A) Uncorrelated relationship between mean and noise not a function of cell size. Cells were first gated using a broad gate capturing all live cells. Subsequent cell size correction by gating in a small region of SSC/FSC space does not alter the observed uncorrelated relationship between expression mean and expression noise (R^2~0). (B) Observed non-poissonian scaling between mean and variance not a function of cell size. We examined the relationship between mean and variance prior to and after cell size correction and find that both exhibit scaling not significantly different from 2 and have similar R^2 values. (C,D) Inference of slope and correlation not a function of gate size. The regression slope and correlation between variance and mean were examined following gating with various size square gates centered about the density mode in SSC/FSC space that capture from 10% to 90% of the total live cells. We do not find a significant modulation of the inferred relationship between mean and variance as a function of gate size.

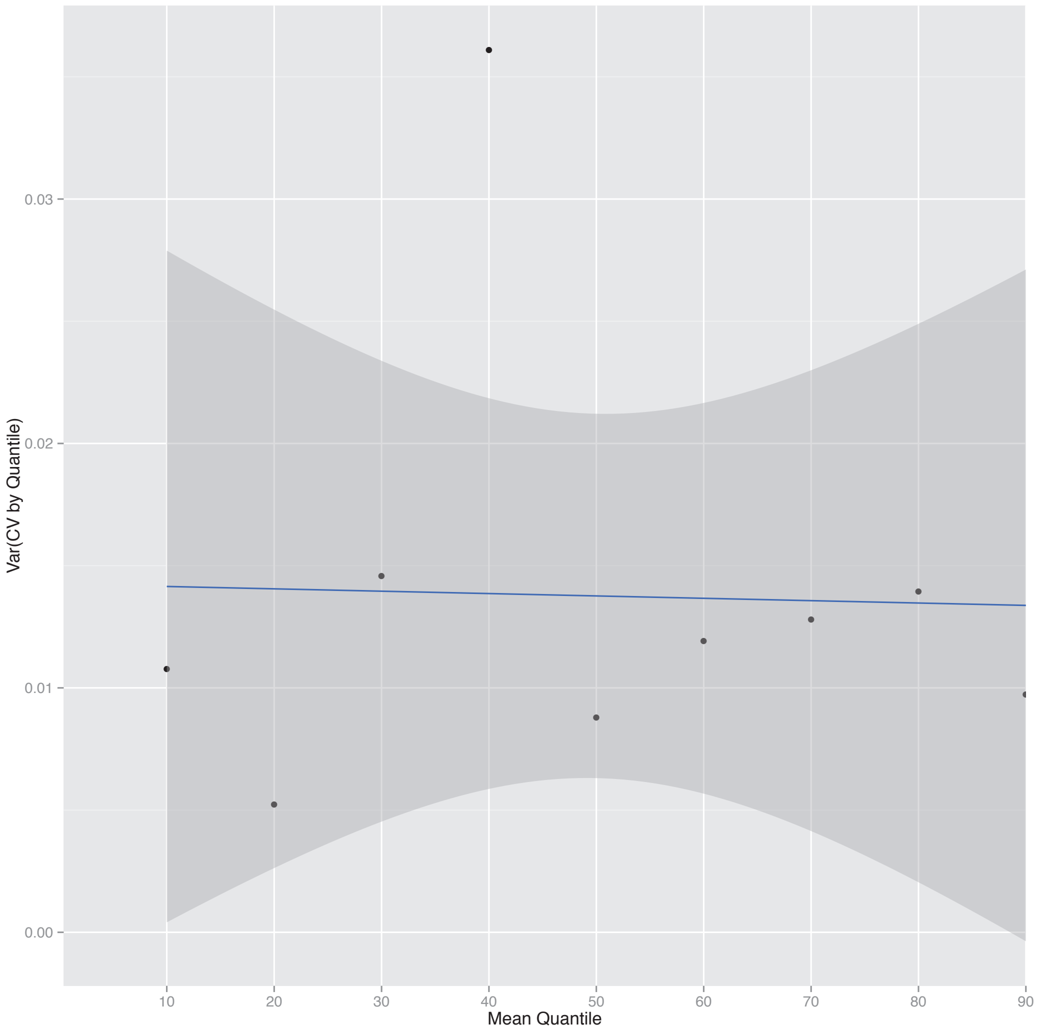

II

Supplementary Figure 5 - Variance of CV is highly similar as a function of Mean. To demonstrate that expression noise is constant as a function of Mean, the Variance of CV as a function of Mean Quantiles (10%) was determined. No correlation between Mean Quantile and Var(CV) is observed and values fall within a narrow band.

II. Independent Clone Generation Replicate

Despite the large number of clones generated, we entertained the possibility that the relationships inferred from this large set of clones may be an artifact of the particular clones generated or the particular culture conditions. Therefore, to address this possibility we generated an independent set of 191 clones. These clones were derived under identical conditions as the main set from an independent infection of uninfected Jurkat T cells with LGM2 virus. We found that the relationships between Mean and Variance (regression slope ~2) and Mean and CV (no significant correlation) were not significantly different from the main set of clones used in this study (Figure 2 and Supplementary Figure S5) Therefore, we do not find evidence that suggests our findings are dependent on the particular set of clones generated or culture conditions.

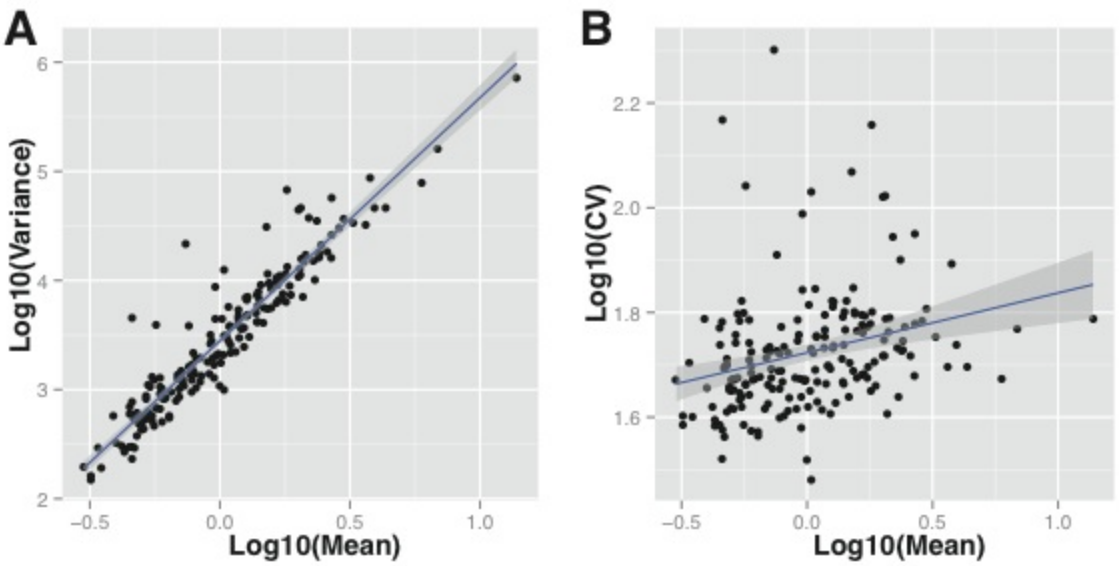

Supplementary Figure 6 - Repeat clone generation and analysis reveals no significant difference from main clones. A set of 191 clones were independently derived using the same protocol described for the main set of clones used in this study and scaling between moments was examined. (A) No significant difference in mean vs. variance relationship. Regression analysis of the mean vs. variance relationship of the independent set of clones reveals a high degree of correlation (R^2=0.89) and no significant difference in slope (p>0.1) from our analysis in the main text. (B) Uncorrelated Mean and CV. In agreement with the main set of clones, we find there to be no significant correlation between expression noise (CV) as a function of mean (R^2=0.08).

III. Clone Selection and Stability

Our initial observation of an uncorrelated relationship between expression noise and mean led us to devise a clone selection method that would: a) capture the range of CV and mean observed and b) permit deeper analysis of the regulation of expression noise for a given mean. Toward this aim, we performed hierarchical clustering and analyzed the mean silhouette value as a function of cluster number (Supplemental Figure S7A, left). We found that the mean silhouette is only weakly dependent on the cluster number with four clusters yielding the highest value. Examining the intra-cluster Silhouette value for each clone (Supplemental Figure S7A, right) reveals those 4 clusters result in broad shoulder of clones with similar Silhouette values and very few clones with negative Silhouette values. Clustering primarily places clones with similar mean expression into the same cluster (Supplemental Figure S7B). For further analysis, pairs of clones were selected from each cluster that had similar mean expression but large differences in their CV (Supplemental Figure S7C). A subset of 25 representative clones were chosen for further analysis to capture the span of expression means and variances observed in the full set of clones.

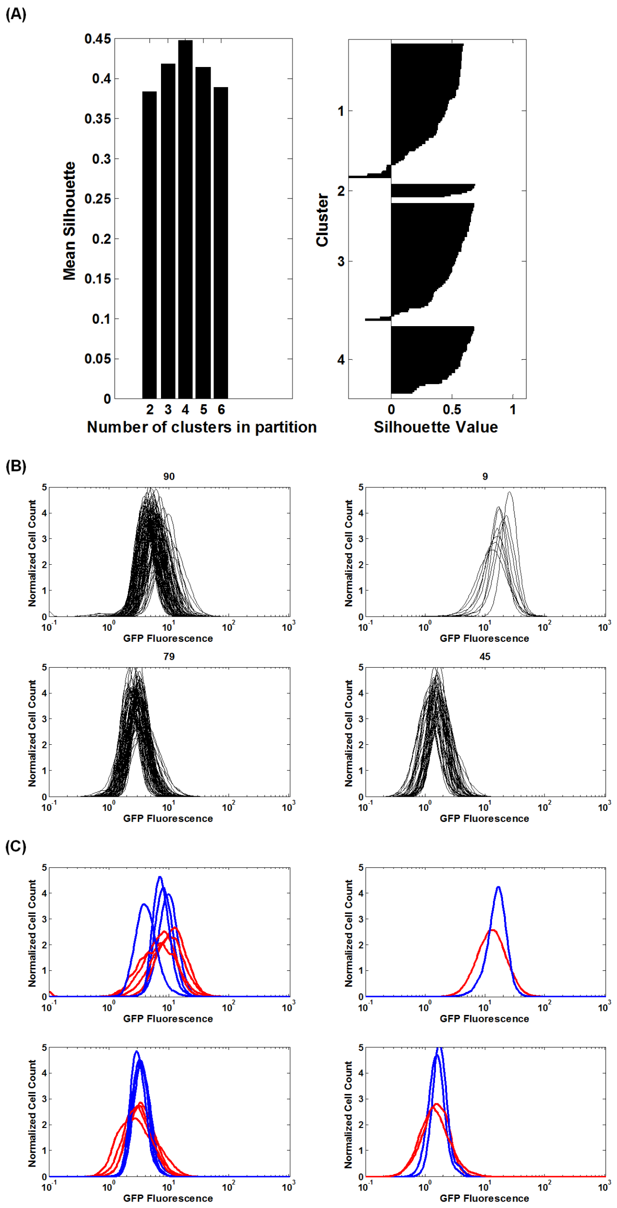

Supplemental Figure 7 - Hierarchical clustering of clones results in four clusters with similar intra-cluster mean but differing CV. Normalized GFP distributions of 223 clones were clustered into different number of clusters using a hierarchical clustering algorithm. (A) *Left* - The mean silhouette shows that the clones are optimally clustered into 4 groups. *Right* - The silhouette value of each clone when clustered into 4 groups. Most of the clones within a group have similar silhouette values with very few clones having negative silhouette values. (B) Each sub-figure represents the normalized GFP distributions of clones that fall within that cluster. The clones get segregated into different clusters primarily on the basis of their mean expression level. The number

above each sub-figure represents the number of clones within that cluster. (C) Pairs of clones were chosen from each cluster to have similar mean but widely differing CV. In this figure, each pair is represented by a blue and red distribution. The GFP distributions in blue indicate clones with low CV whereas the GFP distributions in red represent clones with high CV.

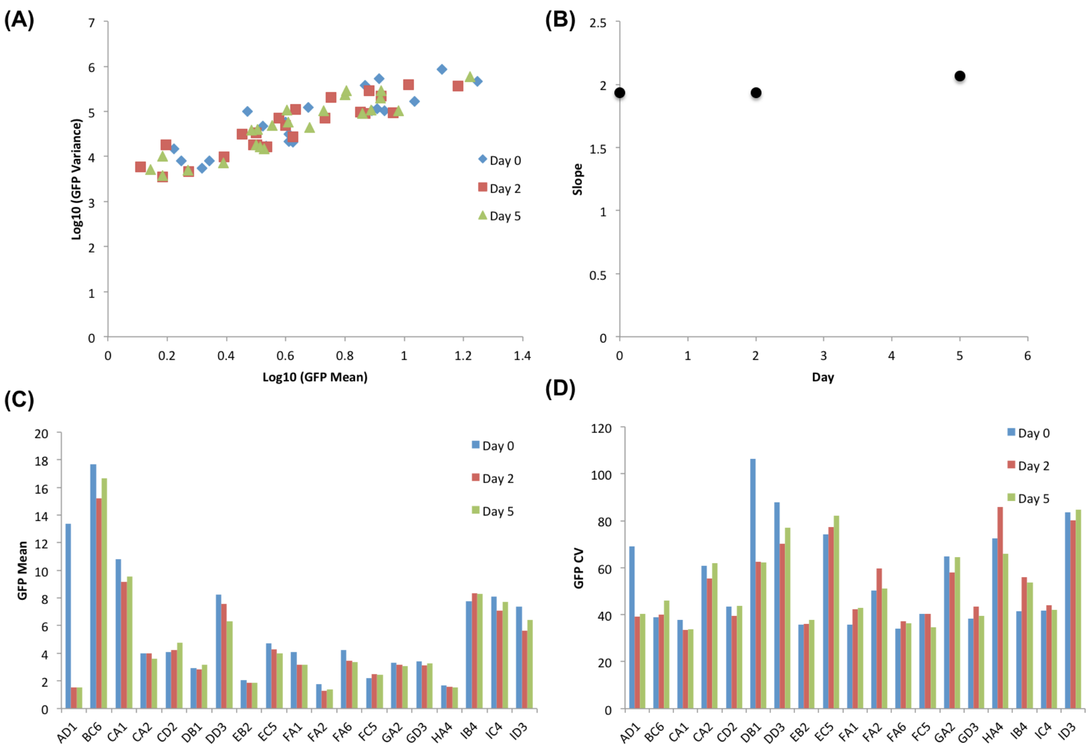

Supplemental Figure 8 - Clones selected for smFISH display stability in mean and CV over 5 days. Cell sorting and expansion produce stable clones that achieve steady state gene expression. GFP distribution for clones that were chosen for smFISH were monitored over 5 days. (A) Each color represents GFP variance vs. mean for all the clones on one particular day. (B) The slope of the GFP variance vs. mean plot remains unchanged over the 5 days with a value of ~2. (C) GFP Mean and (D) GFP CV for individual clones remain unchanged over the 5 days, indicating that they have achieved steady state gene expression.

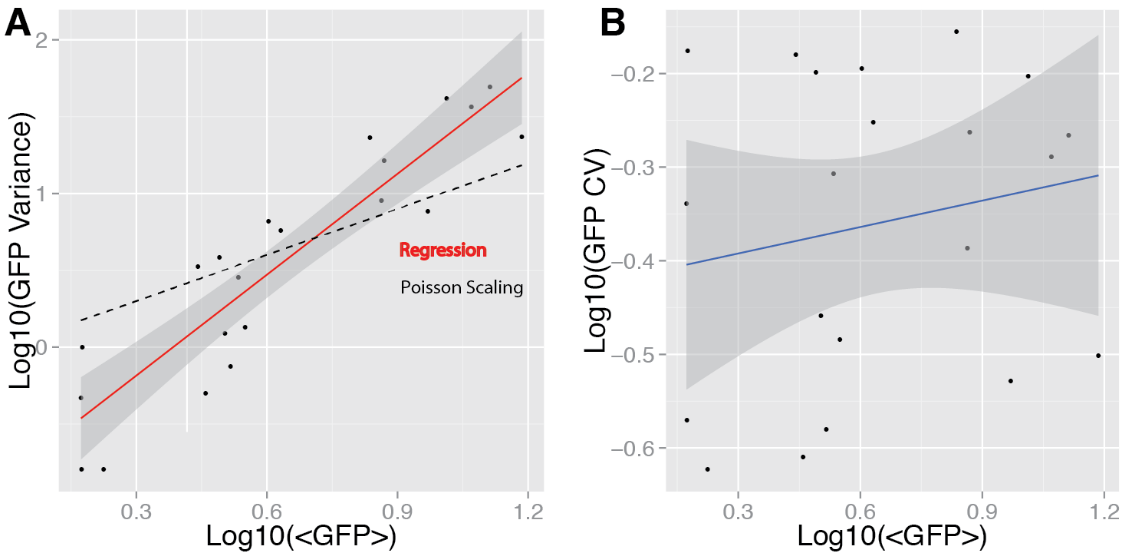

Supplemental Figure 9 - Subset of clones exhibit distribution scaling not significantly different from full set of clones. Following subsetting, the resulting relationships between log-log transformed variance and mean (A, slope=2.03±0.36, R^2=0.85, r_s=0.93, $p<0.001$) and between mean and CV, (B, R^2=0.02, r_s=0.05) indicate that this subset reasonably reflects the scaling relationships observed in the full set of clones. r_s represents the Spearman correlation coefficient for the explanatory and response variables in each pairwise regression while p values represent support for the correlation.

IV. Computational Analysis of Microscopy Images

smFISH enables elegant visualization of single mRNA molecules in fixed populations of cells. However, its application in high-throughput has been hampered in part by the lack of automated software tools. Therefore, to enable the application of smFISH across many clonal populations and over fifteen thousand single cells, we implemented primarily automated cell and smFISH segmentation software in MATLAB (R2011a, Mathworks Inc.) using the freely available DIPImage toolbox (v. 2.2, Linux). DIPImage provides a rich set of basic morphological, intensity and feature based image processing and measurement functions. Importantly, all functions in the toolbox scale to stacks of images such as those resulting from wide-field deconvolution microscopy. We broke down high-throughput image processing into three tasks: 1) deconvolution to enhance signal-noise and correct geometry 2) cell segmentation and 3) smFISH signal segmentation.

High-throughput Deconvolution

The optical properties of wide-field epifluorescent microscopes lead to blurring from out of focus light and geometric distortion from the point-spread-function (PSF) of the light path. We found that deconvolution using a PSF measured using 100nm fluorescent beads (Invitrogen Inc.) significantly enhances the automation of downstream processing. In particular, morphological operations and shape-based classification greatly benefit from the higher signal-to-noise ratio and corrected geometry resulting from deconvolution. Therefore, all 84 fields from each of the 25 LGM2 clones analyzed were first deconvolved using Huygens Core (v3.4, SVI) on a 10-node dual-processor (AMD 2.2Ghz, 2Gb RAM per node) Linux cluster with jobs managed by Sun Grid Engine (Sun Microsystems). Custom scripts in Tcl and Bash issued deconvolution commands and ran jobs. Deconvolution parameters were heurtistically determined to maximize final image quality.

Morphological Image-processing enables the high-throughput analysis of over fifteen thousand single cells across many clones

Previously, owing to the intensive task of image processing, smFISH has been performed on limited numbers of cells across few conditions, which may significantly limit inference of distribution shape and model fitting. We performed smFISH at a large

scale by imaging all 25 clones in 3 channels across 84 fields (capturing ~5-10 cells per field) with 91 axial slices at 0.2 μm spacing. This spacing was determined to provide optimal resolution of smFISH signals and record the entire cell volume. Importantly, performing RNA FISH at this scale presented two challenges: (1) accurate segmentation of cells in fields with touching cells, and (2) reliable identification of single molecule FISH signals across many samples. Previously reported methods involve either z-projection of image stacks, manually intensive threshold selection, or training set development (Raj *et al*, 2006; Rifkin, 2011). Z-projection flattens the recorded image stack and exhibits a propensity to generate overlapping signals that then must be algorithmically separated. Similar to manual threshold selection, training set development requires significant manual intervention. Furthermore, the potential to over-fit limited training data makes scaling to many samples uncertain.

To overcome these issues and enable smFISH at a larger scale, we developed custom software that segments complex fields in a highly automated fashion and reliably identifies smFISH signals with the minor requirement of manual estimation of a ‘ballpark’ threshold that weakly affects the resulting counts. A necessity for a perfect threshold that efficiently discriminates true signals from background is overcome through application of a heuristic multi-feature classifier that represents a model of smFISH signals. Such multi-feature classification proved more time-efficient and robust (relative to signals distinguished by eye) to inclusion of background artifacts across many images and clones than thresholding alone.

Cell Segmentation

Deconvolved image stacks undergo a multi-step process (Supplementary Figure S10) to segment whole cells in the field. Each raw image stack (Supplementary Figure S10A) is first pre-processed using a homomorphic filter (Supplementary Figure S10B) to remove the slowly varying luminance component from the image. Specifically, each image stack is first log-transformed and a heavily blurred (Gaussian kernel with large sigma) copy of this log-transformed image is subtracted. The resulting stack is then transformed back to linear intensity space by exponentiation and linearly stretched to 8-bit gray scale. Subsequently, to remove interior peaks in intensity the image stack is then morphologically reconstructed (Supplementary Figure S10C) with an H-dome (Vincent, 1993), which results in flatter intensity profiles that are less susceptible to over-segmentation. To generate a binary mask, the reconstructed image stack is then

thresholded using an empirically determined multiplier of the *otsu* threshold. Then to separate touching objects, a Euclidean distance transform is applied (Supplementary Figure S10D) and then thresholded using a different multiplier of the *otsu* threshold. This results in unique seeds within each cell (Supplementary Figure S10E). Importantly, to prevent analysis of partial cells, seeds intersecting the image boundary are rejected. We found this method of seeding to be more robust than computing seeds from the DAPI stained nuclei. Specifically, nuclei frequently have interior holes and discontinuities that frequently result in over-segmentation. The resulting seeds were then first grown on the Euclidean transformed image, which effectively results in larger seeds that were then grown on the reconstructed image to arrive at final segmentation of the field (Supplementary Figure S10F). The resulting objects are rejected on the basis of perimeter to area (P2A) and Podczeck circularity shape descriptors, which identify aberrant objects resulting from under or over segmentation. Fields with rejected objects are retained for manual inspection. Retained fields typically comprise less than 5% of total fields. Segmentation problems in these fields can be overcome through a combination of threshold adjustment. Segmentation object boundaries accurately determine cell boundaries (Supplementary Figure S10G). Furthermore, the sizes of the cell objects are not significantly different from clone to clone (Supplementary Figure S11A) and individual cell size distributions are well described by normal distributions (Supplementary Figure S11B). Together, these suggest that this segmentation method is robust across the clones studied and that cell size is not a significant factor underlying our analysis.

smFISH segmentation

Each segmented cell object then undergoes a multistep filtering and classification process to identify both single molecule RNA signals and burst-like features. To remove the majority of slowly varying background remaining in deconvolved images (Supplemental Figure S12A), a morphological TopHat filter with an elliptical structuring element sized (7 pixels) to pass FISH signals but reject background is applied. To further enhance spherical signals a Laplacian of Gaussian transform is applied (Raj *et al*, 2006), resulting in a stack where almost all the background has been removed (Supplemental Figure S12B). What remains are true FISH signals, which are highly spherical, and irregular blobs. For each clone, a 'ballpark' threshold, which provides a reasonable mask of FISH signals and blobs, is manually determined by assessing performance across ten

fields chosen at random. A previously reported method relied on setting a precise threshold for each cell (Raj *et al*, 2006). This method does not scale efficiently to tens of thousands of cells and may be susceptible to user bias. Rather than relying strictly on a threshold, we found that classifying objects on the basis of size, circularity and intra-object grey level intensity standard deviation provided a robust discrimination of FISH objects.

We empirically determined thresholds on these features that yielded classification in good agreement with manual classification. The combination of these three features efficiently distinguishes aberrant blobs from FISH signals. Specifically, to segment FISH signals connected component analysis is performed on the thresholded image. Very small (<30 total pixels) and very large (>300) objects are excluded in this step, which provides an initial classification filter. Subsequently, objects are only accepted if their perimeter to area (p2a) values are highly consistent with a sphere (p2a between 0.96 and 1.02). Secondly, background blobs have very uniform pixel intensity across the object while FISH signals have radially decreasing intensity from the center of the diffraction limited spot. This is captured most succinctly in the pixel grey level standard deviation within each object. Therefore, objects with a grey level standard deviation below an empirical threshold are also rejected. In practice, this classification provide counts <5% different from manual counts and is highly scalable. The combination of a 'ballpark' threshold with a three-feature classification scheme allowed us to scale semi-automated FISH analysis to tens of thousands of single cells.

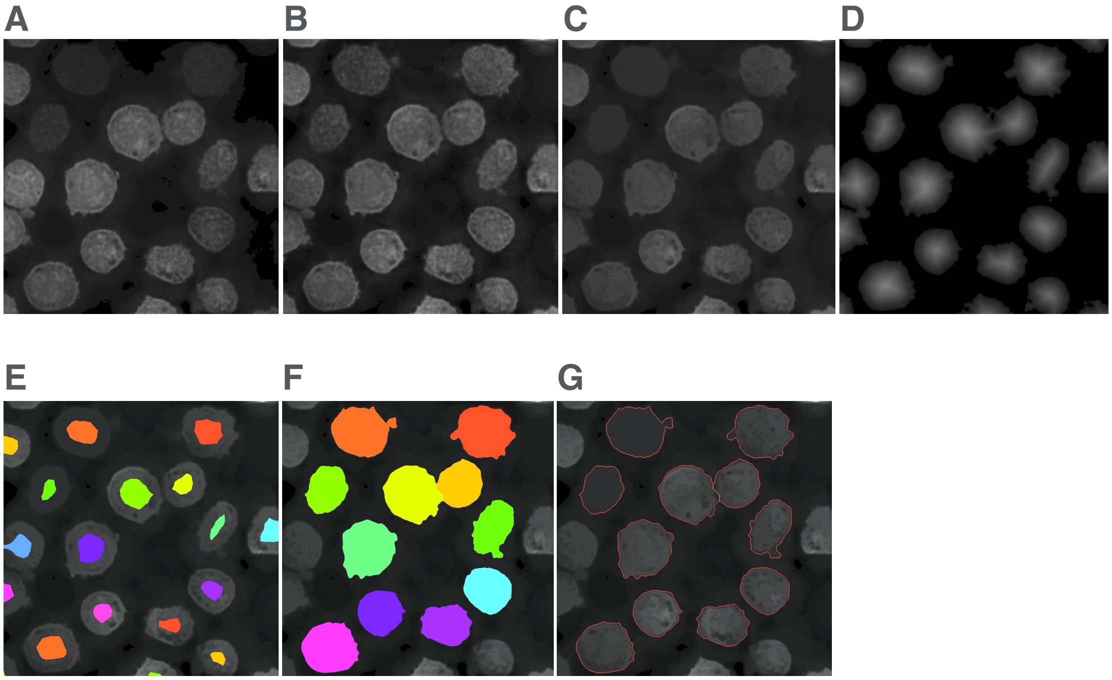

Supplemental Figure 10 - Unsupervised segmentation of cells in fields accurately identifies single cell boundaries. To normalize intensity and reduce intensity noise within cells, deconvolved fields (A) are first pre-processed with a homomorphic filter (B) and then reconstructed with an H-dome structuring element (C). The reconstructed image is then thresholded and a Euclidean distance transform applied (D). Subsequently, the transformed image is thresholded and seeds identified using connected components analysis (E). These seeds are then expanded through a seeded region-growing algorithm to arrive at final segmentation (F), which provides robust identification of single cells and accurately identifies cell boundaries (G). All image are maximum intensity projections, however processing is performed on the entire image stack.

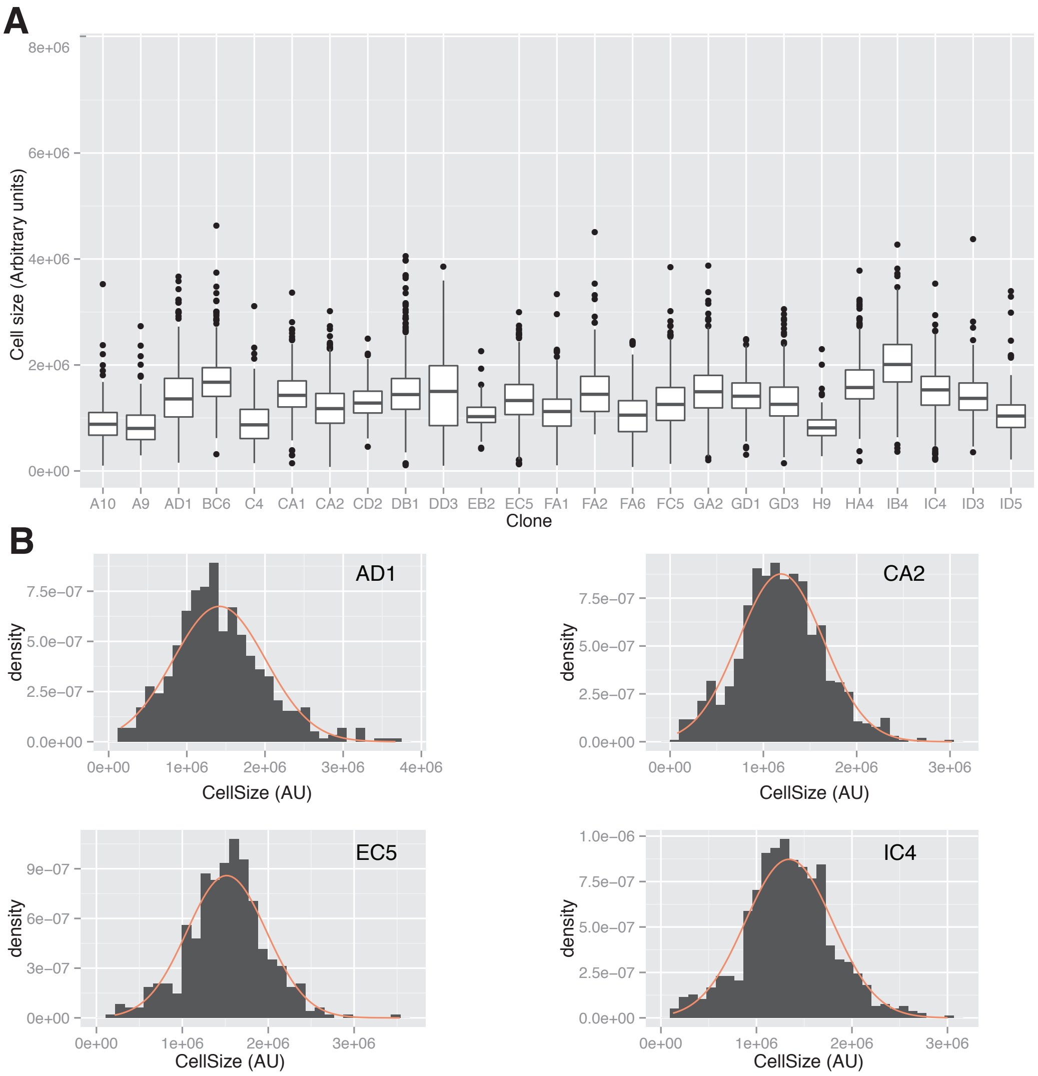

Supplemental Figure 11 - Cell sizes are well described by Normal distributions and mean cell size exhibits low variation across clones. As an initial assessment of the quality of segmented objects, we examined the cell size (sum of segmented object pixels) distribution for each clone. (A) Box and Whisker plots of clone cell size. Examination of Box and Whisker plots of cell size for each of the clones we analyzed reveals little variation (<2) in mean cell size and no apparent trend between either mean RNA copy number or noise. (B) Normal Fits of Cell Size Distributions. The cell size distributions of all clones are well described by a Normal distribution. A subset of clones with Normal fits overlaid on histograms of cell size is shown.

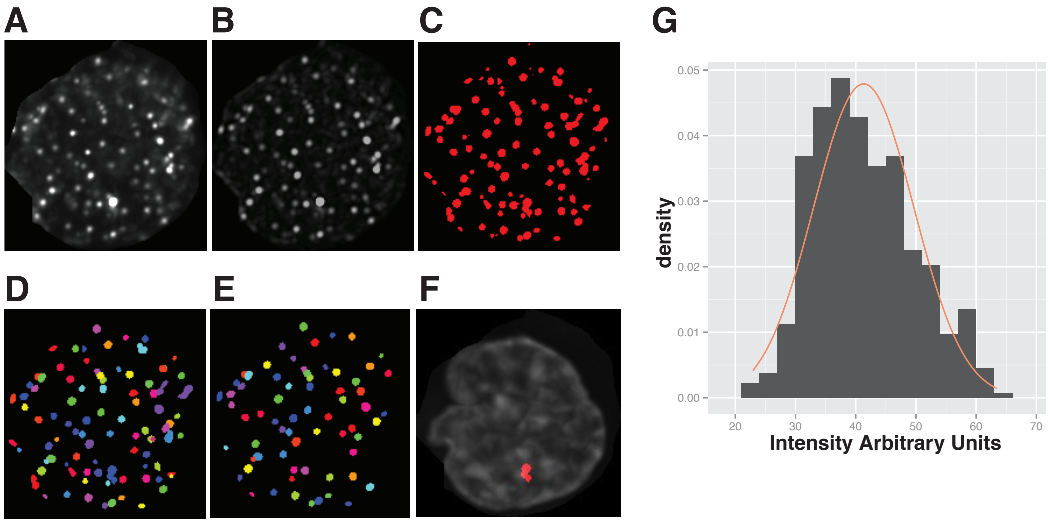

Supplemental Figure 12 - A combination of thresholding and feature based classification permits high-throughput identification of smFISH signals. (A-E) Multistep filtering and segmentation of FISH signals. To remove background and amplify spherical signals in the deconvolved images (A), a TopHat filter and Laplacian of Gaussian transform are sequentially applied (B). A 'ballpark' threshold determined for each clone is used to threshold the filtered and transformed image (C). Connected components analysis identifies all particles between 30 and 300 pixels in size (D). Perimeter to area (P2A) and pixel grey-level standard deviation are used to classify true FISH signals (E) from background objects. (F) Burst identification. On the basis of size, summed pixel intensity and pixel grey-level standard deviation active transcriptional centers ('Bursts') can also be identified. (G) Normally distributed particle intensity. The intensity of the identified smFISH signals are well described by a single Gaussian (red) distribution, suggesting predominantly single molecule signals. The histogram shown represents thousands of particles scaled according to empirical density to permit comparison to Gaussian density.

V. Measurement of RNA Degradation

Basal mRNA degradation rates were determined through the measurement of GFP mRNA abundance in a polyclonal population of HIV-1 LGM2 infected Jurkat T cells treated with 50μM α-amanitin (Sigma A2263). Total RNA samples were obtained by collecting 100,000 cells in TRIzol (Invitrogen 15596-018) at 0, 1, 2, 4, 6, and 8 hours post α-amanitin treatment and then extracted by repeated phenol-chloroform extractions. RNA concentration was quantified with a NanoDrop 1000 spectrophotometer. GFP mRNA abundance was determined by RT-qPCR in triplicate using a QIAGEN QuantiTect SYBR Green RT-PCR Kit (QIAGEN 204243) with a Bio-Rad iQ5 system. 50ng of total RNA and 0.25μM of GFP forward and reverse primers were used according to the supplied instructions at an annealing temperature of 62°C for 45 cycles. To assess the reliability of our measurement, β-Actin mRNA degradation was also measured as a control for normal mRNA degradation rates. We consistently observed an increase in the apparent LGM2 mRNA level at the 2hr time-point, which is likely due to RNA export phenomena as previously reported (Raj *et al*, 2006). Furthermore, cells are markedly sick at the 8hr time-point. Therefore, we only fit the 2hr, 4hr and 6hr time-points. Best-fit estimates of RNA degradation rates were obtained by performing linear regression on semi-log transformed data. Based on this, we find the LGM2 degradation rate to be approximately 0.34 hr^{-1} (Supplementary Figure S13A, regression R^2=0.92) and the β-Actin degradation rate (Supplementary Figure S13B, regression R^2=0.73) to be approximately 0.14 hr^{-1}. Our estimated β-Actin mRNA degradation rate was found to be similar to previously published values (López-Orduña *et al*, 2007). Primer sequences used are shown below.

Primer	Sequence (5' to 3')
β-Actin forward	CCTGGCACCCAGCACAAT
β-Actin reverse	GCCGATCCACACGGAGTACT
GFP forward	AGCAAAGACCCCAACGAGAA
GFP reverse	CGTCCATGCCGAGAGTGAT

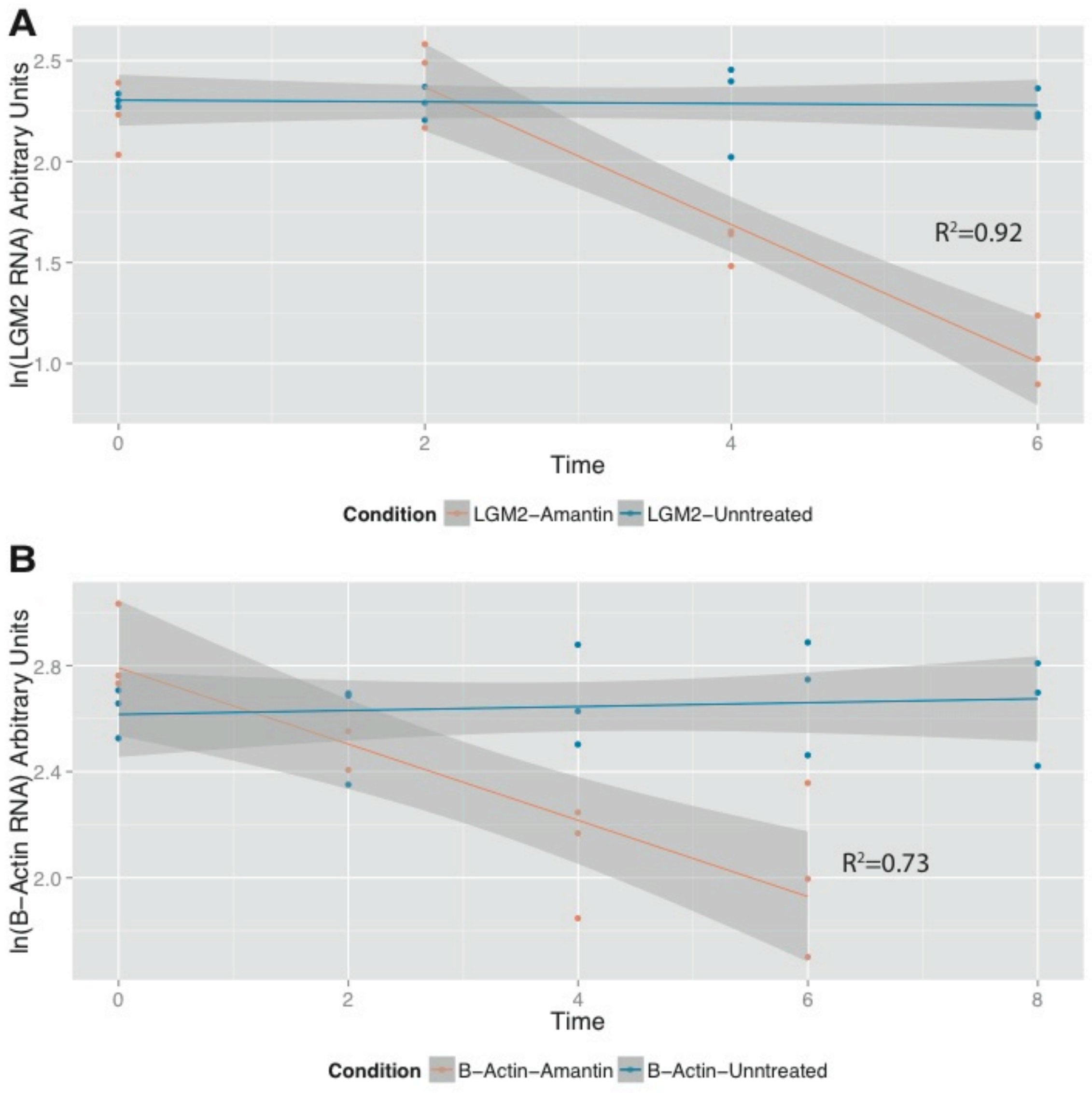

Supplementary Figure 13 - Experimental determination of mRNA degradation rates for LGM2 and β-Actin. (A) LGM2 mRNA degradation. To halt RNA Polymerase II transcription, a polyclonal LGM2 population was treated with 50μM α-Amanitin. Relative RNA levels were determined at 0-6 hours post-treatment for both α-Amantin treated (orange) and untreated (blue) populations through qPCR. Due to a consistently observed increase in LGM2 level at 2hrs, the 0 time-point was excluded from estimation of the degradation rate. The degradation rate was estimated through log-linear regression of the 2, 4, 6 hr timepoints with 3 qPCR replicates per time-point. The best-fit slope is 0.34 hr^{-1} (R^2=0.92), representing a half-life of 2.04 hours. (B) To ascertain whether our system for measuring degradation rate provided a reasonable estimate, we

also measured β-Actin levels following treatment identical to (A). Using Log-linear regression (orange line) we found the best-fit rate to be 0.14 hr^{-1} (R^2=0.73), representing a half-life of 4.95 hours, which is highly consistent with a previously published rate (López-Orduña *et al*, 2007). Shading around the best-fit regression represents the point-wise 95% confidence interval.

VI. Model Fitting

Maximum likelihood estimation (MLE) of the promoter On rate (k_a) and average burst-size (k_{t+}/k_r) for each clone was performed against the full analytical probability density function (*pdf)* of the standard 2-state model of gene expression (Supplementary Figure S14) (Peccoud & Ycart, 1995; Raj *et al*, 2006). Despite it's abstractness, this model has received widespread use due to its comprehensive ability to reveal kinetic mechanisms underlying burst-like transcription. MLE was implemented through specification of the log-likelihood function of the steady-state RNA distribution (Supplementary Figure S14B) in Mathematica 8 (Wolfram Inc.). Parameters were estimated by numerically minimizing the negative log-likelihood of the two-state *pdf* given the experimentally determined RNA distribution for each clone. As discussed, we experimentally measured the LGM2 degradation rate and therefore k_{t-} was fixed at this value. In addition, as previously reported, RNA distributions are insufficient to separately determine the promoter Off rate and the transcription rate. As we and others have done previously (Skupsky *et al*, 2010; Raj *et al*, 2006), we held the transcription rate in the On state constant across clones. We used our previously reported value of 60 hr^{-1} (Skupsky *et al*, 2010). Values ±50% of this fixed value did not significantly change estimates of k_a or burst-sizes, suggesting that our results are highly independent of the particular value chosen.

A

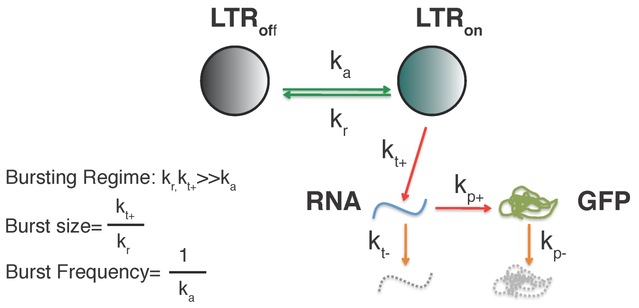

B

$$P(n\ \ RNA)=\frac{\left(\frac{k_{t+}}{k_{t-}}\right)^n \Gamma\left(\frac{k_a}{k_{t-}}+\frac{k_r}{k_{t-}}\right)\Gamma\left(\frac{k_a}{k_{t-}}+n\right)\,{}_1F_1\left(\frac{k_a}{k_{t-}}+n;\ \frac{k_a}{k_{t-}}+n+\frac{k_r}{k_{t-}};\ -\frac{k_{t+}}{k_{t-}}\right)}{\Gamma(n+1)\,\Gamma\left(\frac{k_a}{k_{t-}}\right)\Gamma\left(\frac{k_a}{k_{t-}}+n+\frac{k_r}{k_{t-}}\right)}$$

C

Mean:

$$\mu = \left(\frac{k_a}{k_a + k_r}\right)\left(\frac{k_{t+}}{k_{t-}}\right)$$

Variance:

$$\sigma^2 = \left(\frac{k_a}{k_a + k_r}\right)\left(\frac{k_{t+}}{k_{t-}}\right) + \left[\frac{k_a k_r}{(k_a + k_r)^2}\right]\left[\frac{{k_{t+}}^2}{k_{t-}(k_a + k_r + k_{t-})}\right]$$

Thus, CV is given by:

$$CV^2 = \frac{\sigma^2}{\mu^2} = \left(\frac{k_{t-}}{k_a}\right)\left\{\frac{k_a + k_r}{k_{t+}} + \frac{k_r}{k_a + k_r + k_{t-}}\right\}$$

In the bursting regime (k_r >> k_{t-} and k_r >> k_a), these equations reduce to:

$$\mu = \bar{k}_a b$$
$$\sigma^2 = \bar{k}_a b(b+1)$$
$$\sigma^2 \approx \bar{k}_a b^2 \qquad \text{..... for } b>>1$$

Thus,

$$CV^2 = \frac{\sigma^2}{\mu^2} = \frac{1}{\bar{k}_a}$$

where $\bar{k}_a$ is the normalized burst frequency and b is the burst size.

D

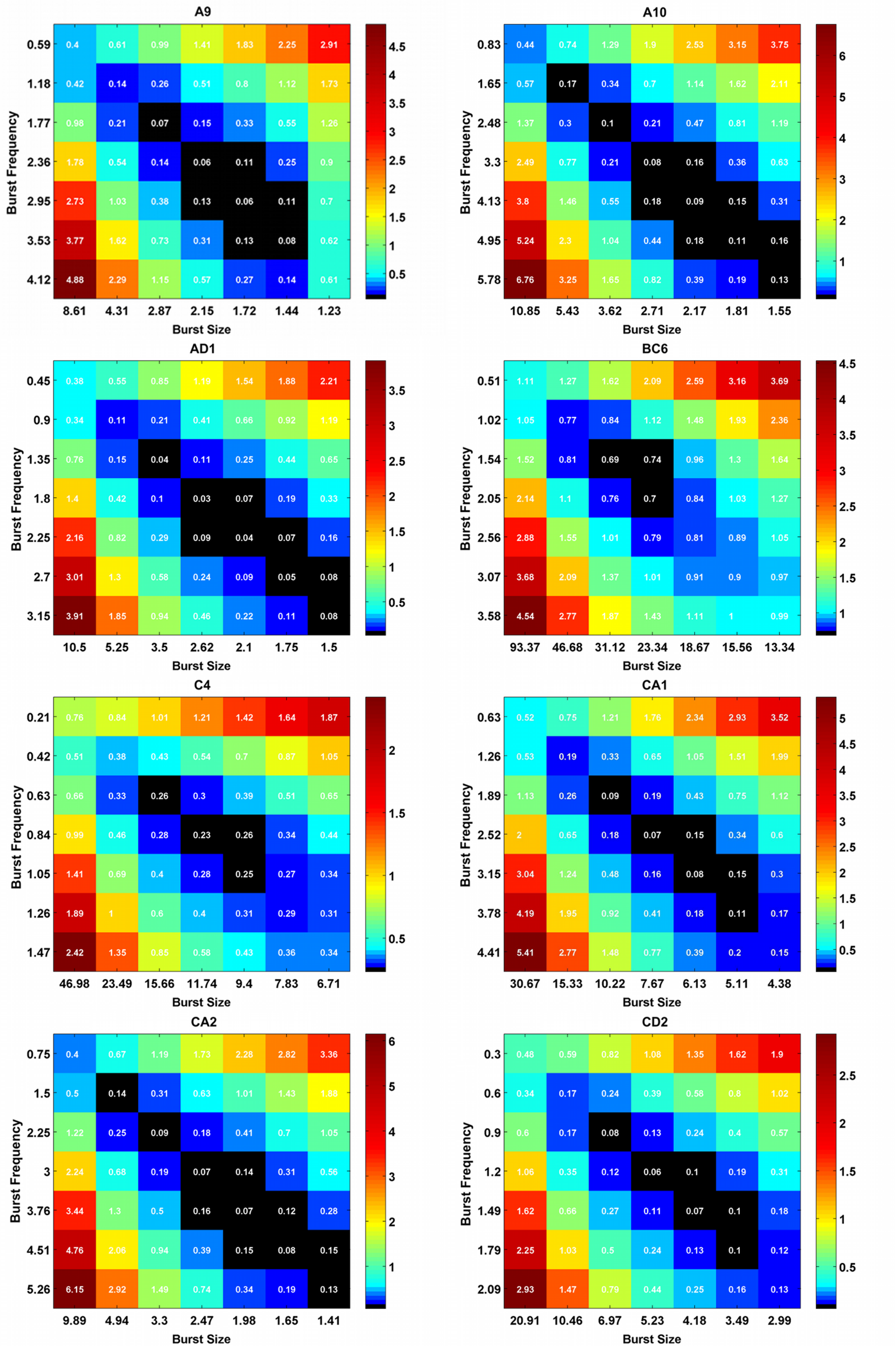

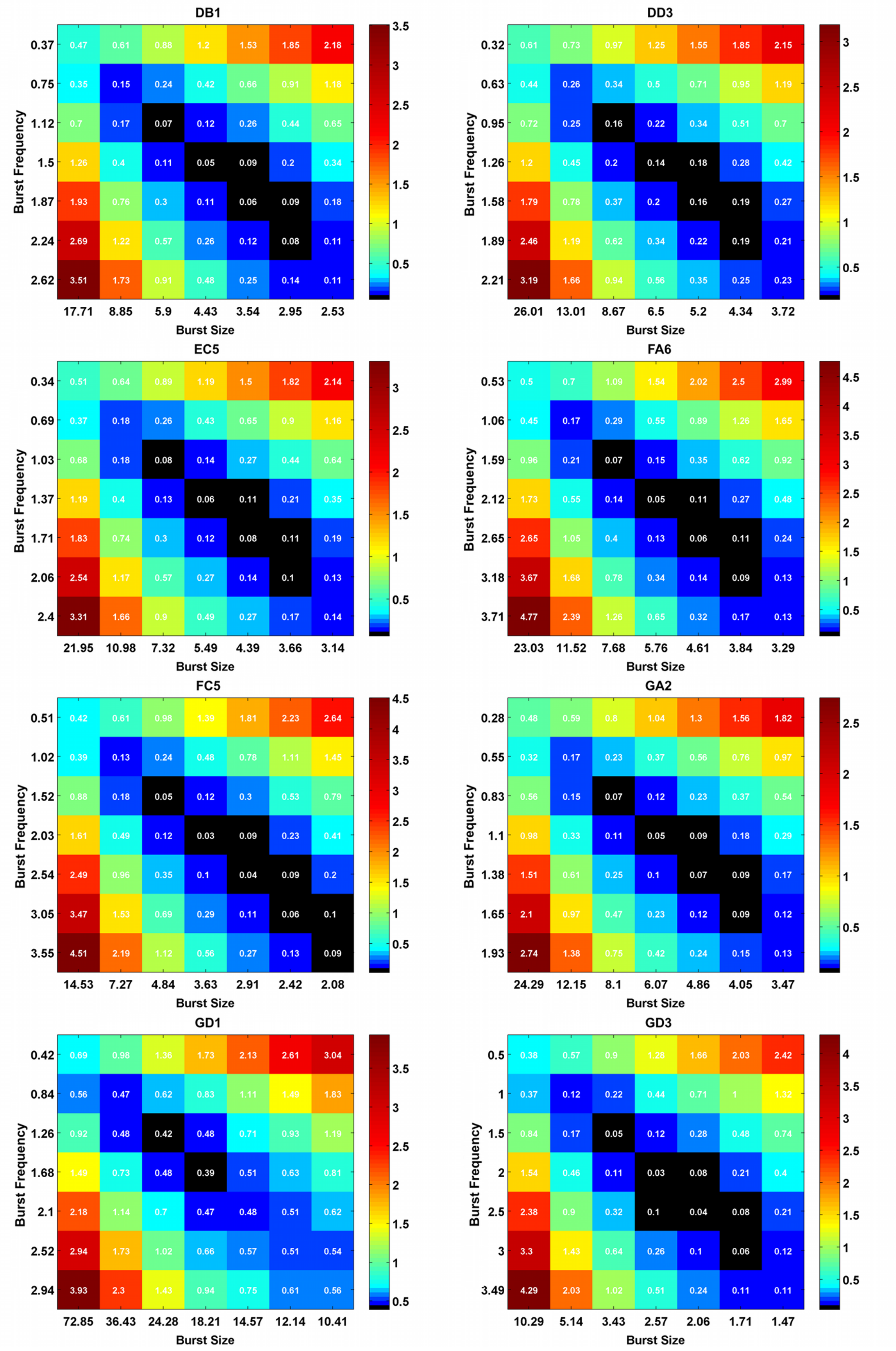
DB1
0.37 0.47 0.61 0.88 1.2 1.53 1.85 2.18
0.75 0.35 0.15 0.24 0.42 0.66 0.91 1.18
1.12 0.7 0.17 0.07 0.12 0.26 0.44 0.65
1.5 1.26 0.4 0.11 0.05 0.09 0.2 0.34
1.87 1.93 0.76 0.3 0.11 0.06 0.09 0.18
2.24 2.69 1.22 0.57 0.26 0.12 0.08 0.11
2.62 3.51 1.73 0.91 0.48 0.25 0.14 0.11
17.71 8.85 5.9 4.43 3.54 2.95 2.53
Burst Frequency
Burst Size
3.5 3 2.5 2 1.5 1 0.5
DD3
0.32 0.61 0.73 0.97 1.25 1.55 1.85 2.15
0.63 0.44 0.26 0.34 0.5 0.71 0.95 1.19
0.95 0.72 0.25 0.16 0.22 0.34 0.51 0.7
1.26 1.2 0.45 0.2 0.14 0.18 0.28 0.42
1.58 1.79 0.78 0.37 0.2 0.16 0.19 0.27
1.89 2.46 1.19 0.62 0.34 0.22 0.19 0.21
2.21 3.19 1.66 0.94 0.56 0.35 0.25 0.23
26.01 13.01 8.67 6.5 5.2 4.34 3.72
Burst Frequency
Burst Size
3 2.5 2 1.5 1 0.5
EC5
0.34 0.51 0.64 0.89 1.19 1.5 1.82 2.14
0.69 0.37 0.18 0.26 0.43 0.65 0.9 1.16
1.03 0.68 0.18 0.08 0.14 0.27 0.44 0.64
1.37 1.19 0.4 0.13 0.06 0.11 0.21 0.35
1.71 1.83 0.74 0.3 0.12 0.08 0.11 0.19
2.06 2.54 1.17 0.57 0.27 0.14 0.1 0.13
2.4 3.31 1.66 0.9 0.49 0.27 0.17 0.14
21.95 10.98 7.32 5.49 4.39 3.66 3.14
Burst Frequency
Burst Size
3 2.5 2 1.5 1 0.5
FA6
0.53 0.5 0.7 1.09 1.54 2.02 2.5 2.99
1.06 0.45 0.17 0.29 0.55 0.89 1.26 1.65
1.59 0.96 0.21 0.07 0.15 0.35 0.62 0.92
2.12 1.73 0.55 0.14 0.05 0.11 0.27 0.48
2.65 2.65 1.05 0.4 0.13 0.06 0.11 0.24
3.18 3.67 1.68 0.78 0.34 0.14 0.09 0.13
3.71 4.77 2.39 1.26 0.65 0.32 0.17 0.13
23.03 11.52 7.68 5.76 4.61 3.84 3.29
Burst Frequency
Burst Size
4.5 4 3.5 3 2.5 2 1.5 1 0.5
FC5
0.51 0.42 0.61 0.98 1.39 1.81 2.23 2.64
1.02 0.39 0.13 0.24 0.48 0.78 1.11 1.45
1.52 0.88 0.18 0.05 0.12 0.3 0.53 0.79
2.03 1.61 0.49 0.12 0.03 0.09 0.23 0.41
2.54 2.49 0.96 0.35 0.1 0.04 0.09 0.2
3.05 3.47 1.53 0.69 0.29 0.11 0.06 0.1
3.55 4.51 2.19 1.12 0.56 0.27 0.13 0.09
14.53 7.27 4.84 3.63 2.91 2.42 2.08
Burst Frequency
Burst Size
4.5 4 3.5 3 2.5 2 1.5 1 0.5
GA2
0.28 0.48 0.59 0.8 1.04 1.3 1.56 1.82
0.55 0.32 0.17 0.23 0.37 0.56 0.76 0.97
0.83 0.56 0.15 0.07 0.12 0.23 0.37 0.54
1.1 0.98 0.33 0.11 0.05 0.09 0.18 0.29
1.38 1.51 0.61 0.25 0.1 0.07 0.09 0.17
1.65 2.1 0.97 0.47 0.23 0.12 0.09 0.12
1.93 2.74 1.38 0.75 0.42 0.24 0.15 0.13
24.29 12.15 8.1 6.07 4.86 4.05 3.47
Burst Frequency
Burst Size
2.5 2 1.5 1 0.5
GD1
0.42 0.69 0.98 1.36 1.73 2.13 2.61 3.04
0.84 0.56 0.47 0.62 0.83 1.11 1.49 1.83
1.26 0.92 0.48 0.42 0.48 0.71 0.93 1.19
1.68 1.49 0.73 0.48 0.39 0.51 0.63 0.81
2.1 2.18 1.14 0.7 0.47 0.48 0.51 0.62
2.52 2.94 1.73 1.02 0.66 0.57 0.51 0.54
2.94 3.93 2.3 1.43 0.94 0.75 0.61 0.56
72.85 36.43 24.28 18.21 14.57 12.14 10.41
Burst Frequency
Burst Size
3.5 3 2.5 2 1.5 1 0.5
GD3
0.5 0.38 0.57 0.9 1.28 1.66 2.03 2.42
1 0.37 0.12 0.22 0.44 0.71 1 1.32
1.5 0.84 0.17 0.05 0.12 0.28 0.48 0.74
2 1.54 0.46 0.11 0.03 0.08 0.21 0.4
2.5 2.38 0.9 0.32 0.1 0.04 0.08 0.21
3 3.3 1.43 0.64 0.26 0.1 0.06 0.12
3.49 4.29 2.03 1.02 0.51 0.24 0.11 0.11
10.29 5.14 3.43 2.57 2.06 1.71 1.47
Burst Frequency
Burst Size
4 3.5 3 2.5 2 1.5 1 0.5

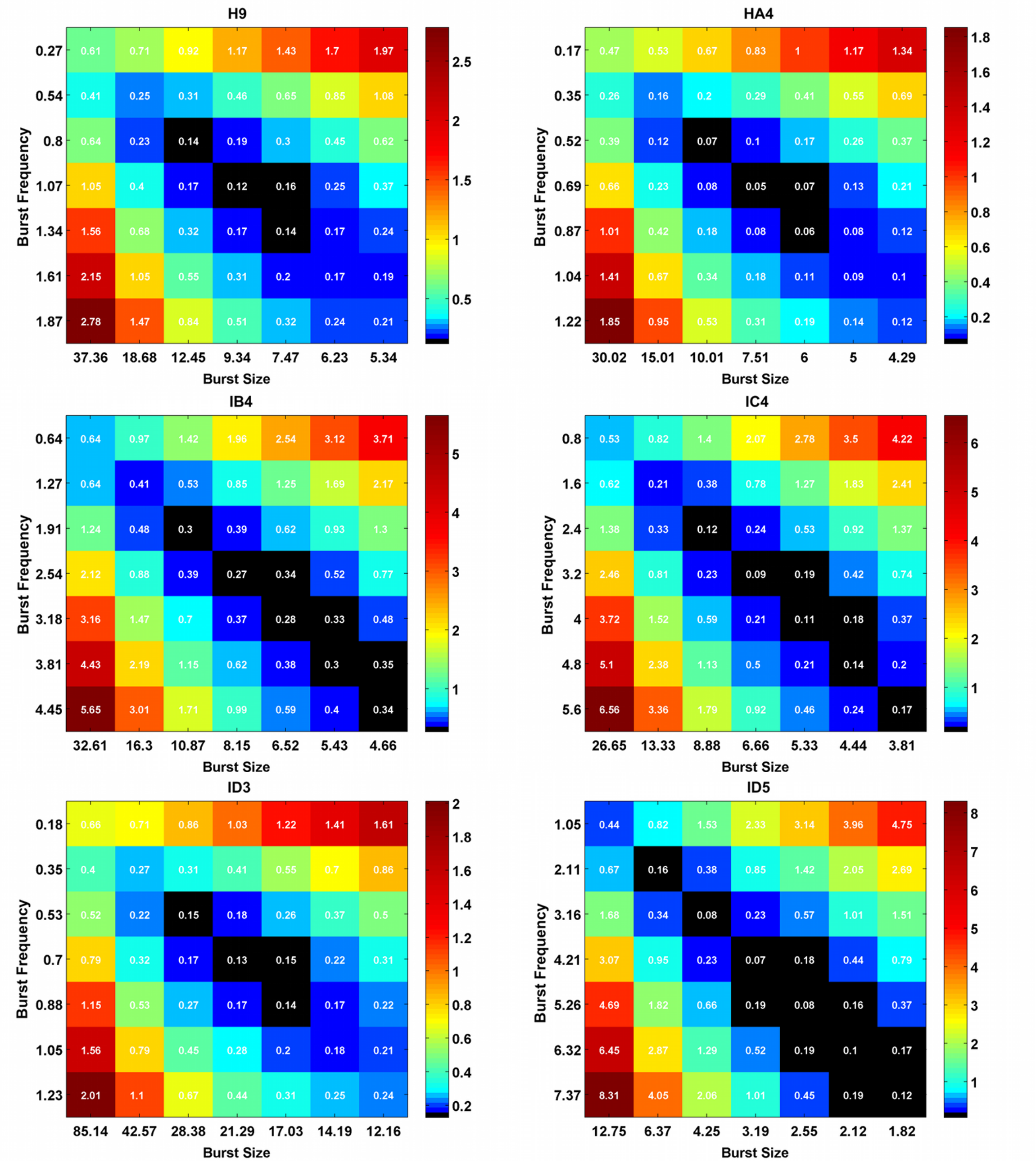

Supplementary Figure 14 - A two-state stochastic model of gene expression. (A) Model configuration. The LTR is allowed to transition between a non-productive 'Off' state and a productive 'On' state with forward and reverse rates k_a and k_r, respectively. RNA is produced in the 'On' state at rate k_t and is degraded by a first-order process at rate k_{t-}. Similarly, protein is produced and degraded at rates k_p and k_{p-}, respectively. Kinetic rates are taken to be transition state probabilities. The steady-state RNA distribution is well described by the frequency of turning on ('Burst-frequency') and the

average number of transcripts produced in the 'On' state ('Burst-size'). The burst frequency describes the rate at which the promoter switches from an inactive (or Off state from which no transcripts are produced) to an active state (or On state from which transcripts are produced at a rate of k_{t+}). The transition from the Off to On state can be thought of as a transition from a repressed promoter to an active promoter state where the transcription machinery binds and RNA polymerase initiates the production of transcripts. The burst size describes the average number of transcripts that are produced every time the promoter transitions to the On state. (B) Steady-state mRNA probability density function. Maximum-likelihood estimation of model parameters was performed against the full RNA probability density function, which represents the steady-state solution to the chemical master equation representing promoter and RNA copy number states but not protein. (C) The first two moments, mean and variance, are shown for the steady-state mRNA probability density functions in Supplementary Figure 14B. Under conditions where infrequent transitions are made to the 'On' state, the lifetime of the 'On' state is short, and transcription is fast relative to the frequency of turning 'On', transcription is 'burst-like'. Under these conditions, the first two moments of the distribution simplify as shown. (D) Sensitivity analysis for the MLE best-fits shown in Figure 4E. Experimental mRNA FISH distributions for each clone were compared to theoretical distributions (Supplementary Figure 14B) for different parameter values of burst frequency and burst size. The Kullback-Leibler (KL) divergence was used to quantify how closely the theoretical distribution with a particular set of parameters approximates the experimental distribution. The Kullback-Leibler divergence is a information theoretic measure that quantifies the extra bits of information required for the theoretical distribution, with two parameters in this case, to approximate the experimental distribution. Smaller KL divergences imply that a particular set of parameters in the theoretical distribution approximates the experimental distribution more closely. For all the clones we observed that the MLE best-fits parameters gave the lowest KL divergences with these divergences increasing in all directions in the 2-D parameter space. The heatmap shows KL divergences for different parameter values (centered around the MLE best-fit parameter values) with the coloring pattern serving as an guide to the eye.

VII. Additional Fit Parameter Analysis

In this study we reveal both observational and mechanistic othorgonality in the control of expression mean and expression noise (CV) across integration positions. Specifically, we find expression mean and noise to be uncorrelated with burst-size primarily explaining mean and promoter On rate primarily explaining noise. Despite the lack of significant cross-correlations between promoter On rate and mean, and burst-size and noise, some possibility of co-modulation remains. Therefore, we directly examined burst size as a function of promoter On rate (Supplementary Figure S15) and found no evidence of significant correlation ((R^2=0.08, p>0.1), (r_s=0-38, p>0.05)). This suggests that burst-size and k_a respectively provide orthogonal explanations for mean and noise.

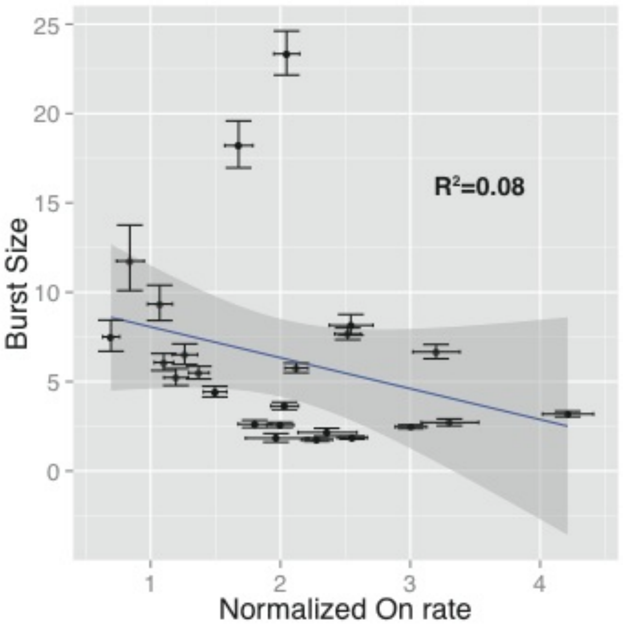

Supplementary Figure 15 - Burst-size is not a significant function of the promoter On rate. To resolve whether burst-size and promoter On rate respectively provide orthogonal explanations of expression mean and expression noise, we examined both linear regression (blue line) and non-parametric Spearman correlation. Neither linear regression (R^2=0.08, p>0.1) or the Spearman correlation coefficient (r_s=-0.38, p>0.05) suggest that there is a significant correlation. Shading around the regression line indicates the point-wise 95% confidence interval.

VIII. Additional Nucleosome Sensitivity Analysis

In this study we established that for similar mean levels of expression, noisier clones have more inaccessible chromatin at the promoter. Closer analysis along different regions of the promoter revealed that the HSS consistently has the highest ratio of chromatin inaccessibility between high- and low-noise clone pairs. This arises from much lower chromatin inaccessibility values for low-noise clones compared to other sites in the promoter (Supplemental Figure S16). We also established that chromatin inaccessibility at Nuc-1, unlike HSS or Nuc-0 (Supplemental Figure S17) is the best predictor of the promoter On rate. This is further reinforced by performing a principal components analysis (Supplemental Figure S18).

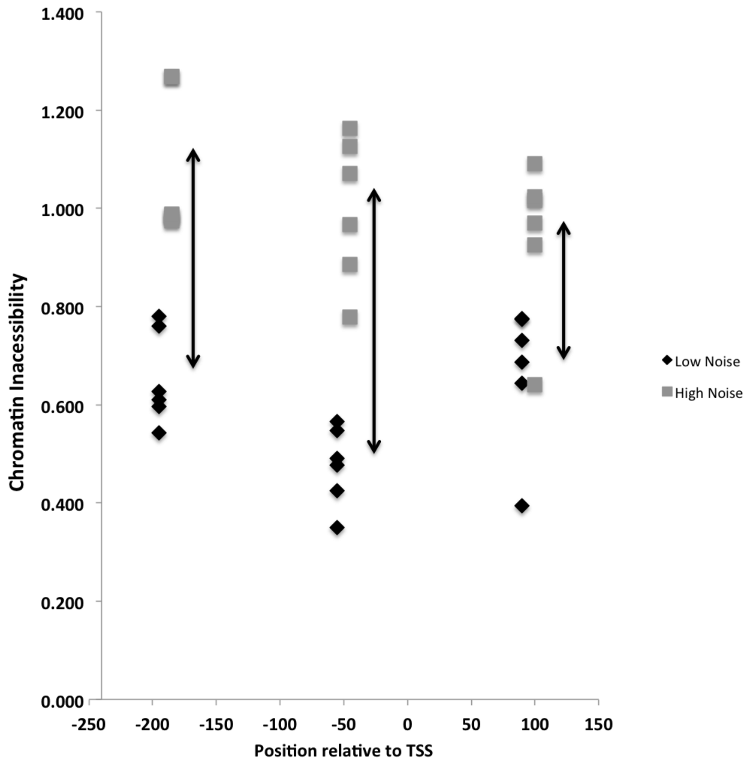

Supplementary Figure 16 - Chromatin inaccessibility for all clones at three positions along the HIV-1 LTR. The figure shows that for all clones and across all positions within the HIV-1 promoter, noisier clones have more inaccessible chromatin. Interestingly, while the mean levels of chromatin remains unchanged across the promoter for the noisier clones, low-noise clones tend to have more open chromatin at the HSS compared to other regions of the promoter. The x-axis corresponds to the midpoint of the amplicons used in qPCR.

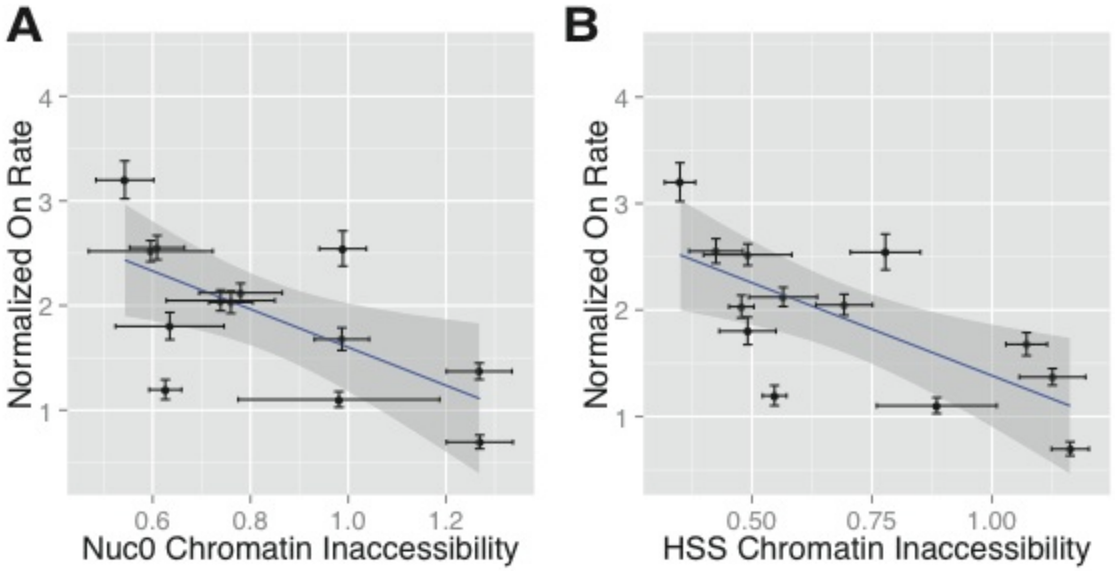

Supplementary Figure 17 - The promoter activation rate does not correlate strongly with the chromatin state at other regions of the HIV-1 promoter. Chromatin inaccessibility at (A) the hypersensitive site (HSS) and (B) the Nuc-0 site does not correlate strongly with the promoter activation rate. This suggests that Nuc-1 plays the most important role in setting the rate of promoter transition from the inactive to active state. All qPCR was performed in triplicate and error bars reflect the standard deviation from the mean. Error bars for the maximum likelihood estimates of k_a represent 95% confidence intervals.

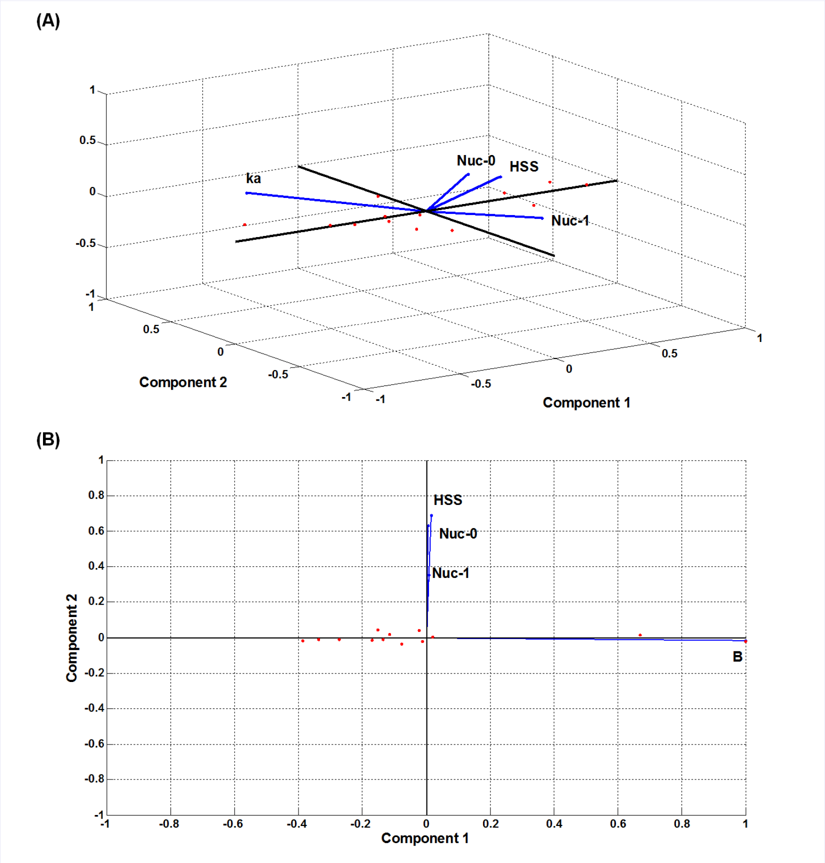

Supplementary Figure 18 - Chromatin inaccessibility at Nuc-1 is the best predictor of the promoter On rate while the burst-size is not correlated to the chromatin state at the promoter. (A) Principal components analysis (PCA) for the promoter On rate and the chromatin state at the promoter shows that most of the data lies within the 2D space of the first two principal components. The axis for chromatin inaccessibility at Nuc-1 is almost negatively correlated to the promoter On rate whereas HSS and Nuc-0 are almost orthogonal to the promoter On rate. Therefore, chromatin inaccessibility at Nuc-1 appears to be the best predictor of k_a. (B) PCA for the burst-size and the chromatin state at the promoter shows that most of the data lies along the 1D axis of the first principal component and that the burst size is independent of the three chromatin inaccessibility measurements along the promoter.

IX. Summary of Statistical Analysis

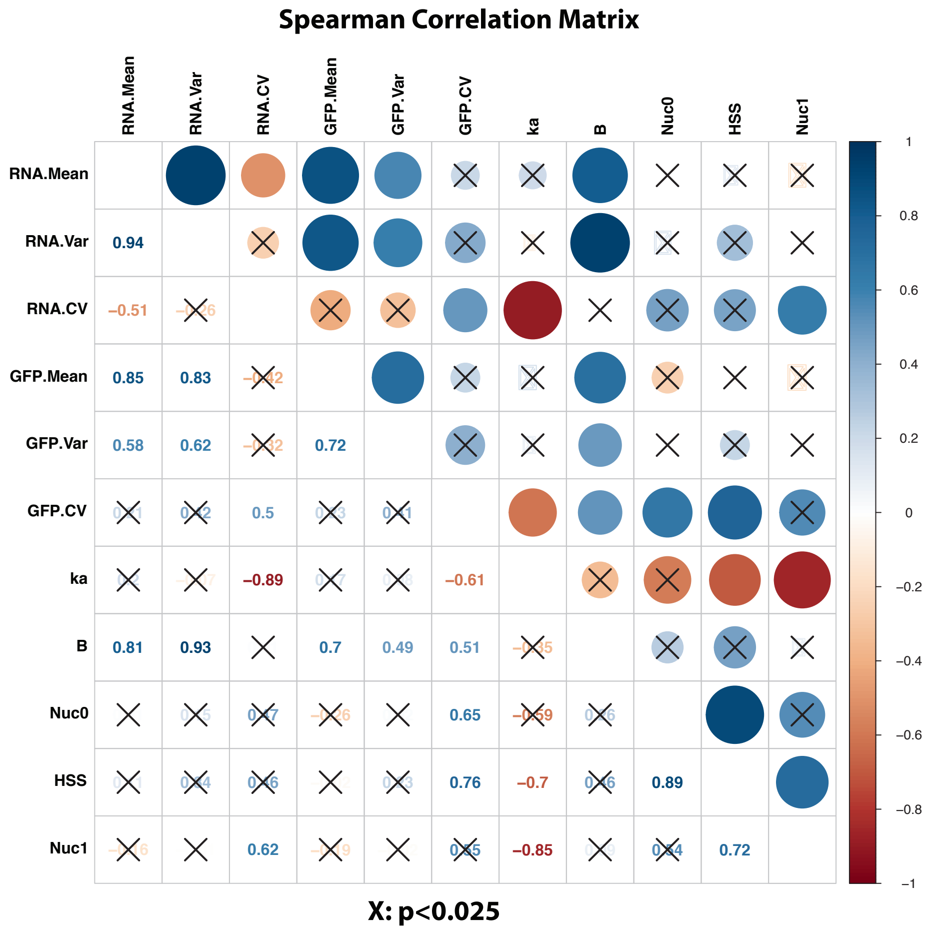

Supplementary Figure 19 - Spearman Correlation Matrix bolsters all major moment and parameter relationships. The top-half of the matrix visualizes Spearman correlation values for each row and column pair as circles where both the size and the color (legend on right) indicate the degree of coloration. The bottom-half of the matrix visualizes the Spearman correlation for each row and column pair as the numerical value and colored accordingly. In both halves of the matrix, correlations for which the p-value is greater than 0.025 are indicated by an ‘X’.

X. Experimentally Measured Protein and mRNA Moments and Model Fit Parameters

Clone	GFP (Mean)	GFP (Variance)	GFP (CV)	Number of Cells (mRNA FISH)	mRNA (Mean)	mRNA (Variance)	mRNA (CV)	Absolute Burst Frequency k_a (hour^{-1})	Burst Size	Chromatin Inaccessibility (Nuc1)	Chromatin Inaccessibility (HSS)	Chromatin Inaccessibility (Nuc0)	Chromatin Inaccessibility (Large Region around TSS)
CA1	9.398	8.432	0.309	1027	17.430	111.004	0.604	0.856	7.667	0.773	0.491	0.596	0.182
CA2	3.420	4.255	0.603	1058	7.126	25.993	0.715	1.021	2.472	1.092	0.966	0.974	0.205
CD2	4.655	3.583	0.407	482	6.041	30.634	0.916	0.406	5.228	0.776	0.547	0.626	0.055
EC5	3.899	9.890	0.807	816	7.217	41.613	0.894	0.466	5.488	1.023	1.126	1.268	0.444
FA1	3.179	1.804	0.422	1050	4.635	13.580	0.795	0.868	1.865	0.643	0.425	0.609	0.056
FA6	3.330	1.240	0.334	1061	11.425	67.679	0.720	0.721	5.758	0.686	0.565	0.780	0.069
FC5	2.323	0.431	0.283	782	7.084	29.483	0.766	0.691	3.633	0.730	0.478	0.760	0.065
GA2	2.881	3.307	0.631	679	6.454	37.508	0.949	0.375	6.073	1.016	0.885	0.981	0.203
GD1	8.318	28.012	0.636	513	26.012	353.836	0.723	0.570	18.213	0.969	1.072	0.987	0.249
HA4	1.421	0.806	0.632	469	5.079	33.701	1.143	0.236	7.506	0.925	1.163	1.269	0.649
IB4	8.262	18.933	0.527	372	18.543	135.074	0.627	0.864	8.152	0.641	0.778	0.989	0.049
IC4	7.778	10.858	0.424	435	19.041	104.307	0.536	1.088	6.664	0.394	0.350	0.543	0.018

Supplementary Table 1 - Experimentally measured protein and mRNA moments and best-fit parameters from fitting the two-state model. The table provides the first

two moments (mean and variance) for the GFP and mRNA distributions experimentally measured by flow cytometry and smFISH, respectively. The MLE best-fit values of absolute burst frequency and burst size are also shown. The table also provides the chromatin inaccessibility values for clones along different regions of the promoter.

References

López-Orduña E, Cruz M & García-Mena J (2007) The transcription of MGAT4A glycosyl transferase is increased in white cells of peripheral blood of type 2 diabetes patients. *BMC Genet.* **8:** 73

Newman JRS & Weissman JS (2006) Systems biology: many things from one. *Nature* **444:** 561–562

Newman JRS, Ghaemmaghami S, Ihmels J, Breslow DK, Noble M, DeRisi JL & Weissman JS (2006) Single-cell proteomic analysis of S. cerevisiae reveals the architecture of biological noise. *Nature* **441:** 840–846

Peccoud J & Ycart B (1995) Markovian Modeling of Gene-Product Synthesis. *Theoretical Population Biology*

Raj A, Peskin CS, Tranchina D, Vargas DY & Tyagi S (2006) Stochastic mRNA Synthesis in Mammalian Cells. *PLoS Biol.* **4:** e309

Rifkin SA (2011) Identifying fluorescently labeled single molecules in image stacks using machine learning. *Methods Mol. Biol.* **772:** 329–348

Sigal A, Milo R, Cohen A, Geva-Zatorsky N, Klein Y, Liron Y, Rosenfeld N, Danon T, Perzov N & Alon U (2006) Variability and memory of protein levels in human cells. *Nature* **444:** 643–646

Skupsky R, Burnett JC, Foley JE, Schaffer DV & Arkin AP (2010) HIV promoter integration site primarily modulates transcriptional burst size rather than frequency. *PLoS Computational biology* **6:**

Vincent L (1993) Morphological grayscale reconstruction in image analysis: applications and efficient algorithms. *IEEE Trans Image Process* **2:** 176–201

Weinberger LS, Burnett JC, Toettcher JE, Arkin AP & Schaffer DV (2005) Stochastic Gene Expression in a Lentiviral Positive-Feedback Loop: HIV-1 Tat Fluctuations Drive Phenotypic Diversity. *Cell* **122:** 169–182
