# Supplementary material for: Orthogonal control of expression mean and variance by epigenetic features at different genomic loci
Source: Mol Syst Biol. 2015 May 5;11(5):806. doi: 10.15252/msb.20145704 (PMC4461400; doi:10.15252/msb.20145704)
Supplement: Supplementary file 3 [file msb0011-0806-sd3.pdf]

molecular systems biology

Orthogonal control of expression mean and variance by epigenetic features at different genomic loci

Siddharth S. Dey, Jonathan E. Foley, Prajit Limsirichai, David V. Schaffer, Adam P. Arkin

Corresponding author: David V. Schaffer, University of Calfornia, Berkeley

Review timeline:

Submission date:	19 August 2014
Editorial Decision:	10 October 2014
Revision received:	29 January 2015
Editorial Decision:	04 March 2015
Revision received:	25 March 2015
Accepted:	31 March 2015

Editor: Maria Polychronidou

Transaction Report:

(Note: With the exception of the correction of typographical or spelling errors that could be a source of ambiguity, letters and reports are not edited. The original formatting of letters and referee reports may not be reflected in this compilation.)

1st Editorial Decision 10 October 2014

Thank you again for submitting your work to Molecular Systems Biology. We have now heard back from the three referees who agreed to evaluate your work. As you will see from the reports below, the referees acknowledge that the presented findings seem potentially interesting. However, they raise a series of concerns, which should be carefully addressed in a revision of the manuscript. The referees' recommendations are clear in this regard.

Reviewer #1:

In the manuscript entitled "Orthogonal control of expression mean and variance by epigenetic features at different genomic loci", Dey et al integrate a reporter gene at many different locations within human genome and then explore how the noise characteristics of its expression varies across different integration sites. HIV LTR is used both as a mean to drive single-copy insertions at nearly random locations in the genome and as a promoter to drive the expression of reporter. Although similar approach has previously been used by this and other groups, present study offers some new insights. In particular, they show that mean of mRNA (or protein) population in single cells is not correlated with coefficient of variation across integration sites. By the independence of these statistical parameters authors infer that cells are able to control the expression mean by independently controlling the size of gene expression bursts and the "on" rate of the promoter switching (the terminology refers to an earlier two-state promoter switching model). Finally, the authors explore how the distributions of nucleosomes over the promoter sequence will confer such an ability on the reporter.

This is an interesting work that goes further than the author's earlier work in exploring the molecular

© European Molecular Biology Organization

origins of eukaryotic gene expression noise. The model experimental system is "clean" in that the effects of different chromatin contexts can be studied for the same promoter. However, it is limited because the natural genes have different promoters, as well as, different genomic locations. The quality of experimental work appears to be high.

I have a few concerns:
1. The conclusions differ from a number of previous studies. The differences and what they are attributed to should be carefully considered (by other reviewers). For example, Dar et al PNAS, 2012, conclude that both the frequency and size of the transcriptional bursts varies equally across the human genome, independent of promoter sequence. Perhaps the only way to reconcile these different interpretations is to not abstract the on rates and burst sizes from statistical parameters, as both of these studies do, but to measure them directly in live single cells (by approaches like MS2 or molecular beacons). Similarly, differences with Sutter et al 2011, and Bar-Even et al 2006 should be considered.

2. Integration of HIV genome (and therefore the authors reporters) is more likely to occur in relatively open chromatin, therefore full range of different chromatin contexts are unlikely to have been sampled in the present set of clones. I think there are studies related to this issue in HIV literature perhaps the authors should point to that caveat.

3. The description of figure 4E was confusing. The pairs of histograms are presumably from clone pairs that have the same mean but different noise. This should be clearly indicated both in the legend and in the relevant text. It would be cleared if they dropped prefix LGM2 from the clone names in this figure as they did in figure 6 B-E. Also, are the same set of clones being described in the two figures?

4. A basic prediction is that clones with more accessible promoter regions will have higher level of expression. With that in mind, shouldn't a graph of levels of mean expression with the accessibility be provided for all clones? Closest figure to that is S18 but it does not directly address that issue.

5. What is author's view on how the same promoter ends up with different nucleosome distributions depending on the chromatin context.

Reviewer #2:

Dey and colleagues report an interesting experiment: a lentiviral-based reporter vector driving GFP was integrated in different (random and uncharacterized) positions in the genome. 227 clones were selected, and their single-cell distribution of GFP fluorescence was measured to define the mean expression and expression noise characterizing each of the clones. Out of those, 25 clones were selected for measuring the transcription levels using smFISH. This resulted in two main findings: (1) expression noise (CV) is uncorrelated with mean expression, (2) when comparing (six) clone pairs of similar expression, higher noise was associated with repressed chromatin (measured by DNase I sensitivity).
This experiment is interesting and presents a substantial amount of high-quality work.
From result (1), the authors conclude that the noise does not result from a simple Poisson process. They fit the data to a two-state model of gene expression, and from this derive the burst size and burst frequency characterizing each clone. From that, they concluded that noise depends only on burst frequency, while mean expression depends (mostly) on burst size. This differs from previous results in mammalian cells, and also departs from results in model organisms including yeast and bacteria.
I see a potential problem with the analysis in that the authors did not consider the possibility of external noise (noise that results from variability upstream to the promoter, e.g. cell size, amount of ribosomes, transcription factors etc.). This extrinsic noise is not correlated with expression and will therefore provide the same lack of correlation between noise and mean expression, as reported here. In fact, also in yeast and bacteria, where the scaling of noise with expression is well established, a regime of independency exists for high expression where extrinsic noise dominates. This is

discussed in very many papers. So one possible interpretation of the lack of correlation is that extrinsic noise is very high, or we are in this regime where extrinsic noise dominates. The authors should include this possibility in their model and make sure that it cannot explain their data. (In this context, error bars are missing from figure 2 but are in fact quite essential - in particular the [likely] error in estimating noise. Such error-bars are typically generated by re-growing the same clones several times and comparing the distributions; likeliness of different models should be compared relative to those experimental errors)
With regards to the two-state models: it is nice that that the model can fit the disrtibutions, but I wonder how restrictive this fit is, since there are enough parameters one can adjust. A more intuitive explanation of the model will also help. A well appreciated result is that in the two-state model, noise^2 is given by 1/[burst-frequency], while mean expression is given by [burst_size* burst_frequency]. This should be mentioned and explained. In this context, the work of Johan Paullson who played a central role in setting up this analysis should be cited. The work of Nir Friedman et al (PRL) is also highly relevant and should be cited.
When considering the result mentioned above, the fact that noise correlates with burst frequency may be a the result of the fitting procedure, so I'm not sure its biological interpretation is justified. Mean expression is explained by the combination of both burst size and burst frequency, and the fact that, overall, expression changes more than noise, will result in a dominating effect of burst size, as observed. Again, one possibility that the author should consider is that this is the mere result of fitting, and not the only biological interpretation. A more rigorous evaluation of this point is therefore needed, in particular considering the possibility of extrinsic noise, as I suggested above, but perhaps additional possibilities.

Additional remarks:

1. How were the error-bars measured for mRNA expression/noise? do they reflect measurements on different days (should be)

2. The effect if chromatin on burst size was discussed quite extensively in the yeast model where promoter structure was shown to strongly correlate with the normalized-noise, which, by definition, is burst size. This should be mentioned. (e.g. hornung et al. Mol Cell 2012 ; Tirosh and Barkai Genome Research 208 or other related papers)

3. The discussion is too much centered on comparison with previous studies. This will greatly interest the experts, but less so the general reader. I would suggest the authors to (perhaps more briefly) discuss the disagreement in the results/ supplementary and devote the discussion to the more general implications of their results.

Reviewer #3:

Dey et al explore the causes that explain variations in noise due to chromosomal position effect on a lentiviral gene expression cassette. In particular, they find that nucleosome accessibility is decreased between clones displaying high noise, and it is the on-rate in the two-state promoter model that mediates this effect. While chromosome position affects burst size to modulate mean expression levels (as already indicated in their earlier study), burst frequency explains those variations in noise that deviate from the constant trend line of CV values as the mean is varied.
While the main findings on nucleosome accessibility and corollaries are interesting it is often difficult to find the data that would permit to assess the consistency of the observations and to compare to other studies.

I suggest the following changes before publication:

1. Relevant range of parameter values. It is very difficult to collect the parameter values that have been fitted for the clones.
A. Figure S13: Negative values for mRNA decay rates are presented. Even if regression is log-

© European Molecular Biology Organization

linear, which is quite common to fit decay rates, the values for all biochemical rates should be presented in original (non-transformed) positive numbers.
B. Figures 5, 7: On rate is normalized to RNA decay rate. Quite surprisingly, absolute on rates are never presented even though they can be easily calculated. There should be a (supplementary) table provided where all the fitted parameters (in Figure S14) should be presented for a set of clones (e.g. for those shown in Figure 7). These values will permit verifying predictions by time-series measurements. If the absolute values of off-rates and transcription rates cannot be assessed independently then their ratios (burst size) should be indicated.

2. Relevance of the study. Both in the introduction and discussion, the results are compared to studies that explore genome wide trends of different genes (or promoters). On the other hand, the promoter is identical in the different clones in this study and only chromosomal position is varied. While it is reasonable to compare these two types of studies, there is no need to expect consistency between the trends found in these studies. Furthermore, expression driven by different promoters often spans a range of more than three orders of magnitudes. On the other hand, the majority of the clones in this study spans a range of one order of magnitude.

3. There is no any discussion of potential mechanisms that could account for the central message of the paper, the orthogonal control of expression mean and variance, even though there are compatible mechanisms known. Epigenetic modification is one of the major mechanisms known to modulate nucleosome occupancy as a function of chromosomal position since such modifications can spread along the chromosome. For example, epigenetic silencing in yeast can result in orthogonal control of expression mean and variance reactions (Kelemen et al (2010) PLOS Biology, Spatial epigenetic control of mono- and bistable gene expression.) Bimodal expression (and hence large noise) can arise when epigenetic modifiers are recruited on both ends of the gene (bilateral recruitment). Conversely, noise is smaller when the modifiers are recruited only on one end of the gene (unilateral recruitment), even though the mean of expression level can be reduced massively by this form of silencing. Silencing in yeast can also decreases nucleosome accessibility.
The authors claim that the telegraph model is "abstract". It would be useful to provide a couple of statements how the interpretation of data will change if the transition between the two states is not a simple linear process.

Minor comments:
A. CV values should be shown consistently. Since CV values are already normalized they should be shown in linear scale (just as in Figure 5). On the other hand, the logarithms of CV values are shown in other figures.
B. Those clones to highlight/label in Figure 2 that have been selected for the nucleosome experiments (Figure 6, 7).

1st Revision - authors' response 29 January 2015

(see next page)

We thank the reviewers for finding the results presented in this manuscript interesting and for their positive feedback. Below, we have addressed the concerns of the reviewers in detail. Reviewer comments are *italicized*:

Reviewer 1

In the manuscript entitled "Orthogonal control of expression mean and variance by epigenetic features at different genomic loci", Dey et al integrate a reporter gene at many different locations within human genome and then explore how the noise characteristics of its expression varies across different integration sites. HIV LTR is used both as a mean to drive single-copy insertions at nearly random locations in the genome and as a promoter to drive the expression of reporter. Although similar approach has previously been used by this and other groups, present study offers some new insights. In particular, they show that mean of mRNA (or protein) population in single cells is not correlated with coefficient of variation across integration sites. By the independence of these statistical parameters authors infer that cells are able to control the expression mean by independently controlling the size of gene expression bursts and the "on" rate of the promoter switching (the terminology refers to an earlier two-state promoter switching model). Finally, the authors explore how the distributions of nucleosomes over the promoter sequence will confer such an ability on the reporter.

We thank the reviewer for appreciating the new insights presented in this work, including where we showed that the observed independence of expression mean and coefficient of variation (CV) can be explained by the orthogonal control of mean expression by burst size and CV by burst frequency.

This is an interesting work that goes further than the author's earlier work in exploring the molecular origins of eukaryotic gene expression noise. The model experimental system is "clean" in that the effects of different chromatin contexts can be studied for the same promoter. However, it is limited because the natural genes have different promoters, as well as, different genomic locations. The quality of experimental work appears to be high.

We thank the reviewer for finding our experimental system clean and for finding the data to be of high quality.

I have a few concerns:

1. The conclusions differ from a number of previous studies. The differences and what they are attributed to should be carefully considered (by other reviewers). For example, Dar et al PNAS, 2012, conclude that both the frequency and size of the transcriptional bursts varies equally across the human genome, independent of promoter sequence. Perhaps the only way to reconcile these different interpretations is to not abstract the on rates and burst sizes from statistical parameters, as both of these studies do, but to measure them directly in live single cells (by approaches like MS2 or molecular beacons). Similarly, differences with Sutter et al 2011, and Bar-Even et al 2006 should be considered.

We thank the reviewer for raising this point. In our manuscript, we have paid particular attention in noting differences in results from previous studies and the possible reasons for these discrepancies. A critical and concerning underlying assumption that led Dar *et al.* to conclude equal modulation of burst size and burst frequency across genomic locations was based on the assumption that clustered polyclonal populations (i.e. virus integrated in many genomic locations) with similar CV are biologically similar to an isogenic clone (i.e. virus integrated at the same location). Therefore, it is unclear how such a clustering procedure and the use of polyclonal populations (instead of isogenic populations) could influence the estimation of the kinetic parameters in the two-state model. Furthermore, the use of destabilized (and thus considerably less sensitive) GFP to measure gene expression limits their analysis to clones with very high expression, and this bias only towards genomic locations that enable high expression levels (burst sizes of >100 for most clones lie at the higher end of LTR driven gene expression) can also artifactually impact their results.

In contrast, our use of isogenic populations that span a large range of mean gene expression levels, together with direct measurement of both single mRNA molecules and protein, potentially reduces biases introduced from these confounding factors. This is the most probable cause for the differences in results between previous work (Dar *et al*, 2012) and our results, as we note in the Discussion section.

In addition, previous results from our group and other groups (Dar *et al*, 2012; Skupsky *et al*, 2010; Singh *et al*, 2010a) have shown that genomic locations regulate burst size and/or frequency. This stands in contrast to work showing that cis-regulatory elements, but not the epigenetic features at a genomic region, influence gene expression noise (Suter *et al*, 2011). As discussed in our manuscript, this latter conclusion was based on analyzing a few isogenic clones that were treated with trichostatin A (TSA), a highly cytotoxic histone deacetylase inhibitor that globally remodels chromatin and therefore has unknown molecular effects at the genomic locations studied. Furthermore, careful analysis of Suter *et al.*'s supplementary dataset shows that a few clones exhibit large changes in burst size and transcription rates upon stimulation with TSA, implying that the chromatin environment could influence gene expression noise. This suggests that their conclusions are likely influenced by the small sample size, the use of TSA, and indirect protein measurement (as opposed to mRNA measurements) to estimate transcriptional kinetic parameters. Taken together, work by Suter *et al.* and other previous work (Dar *et al*, 2012; Skupsky *et al*, 2010; Singh *et al*, 2010a) implies that both the chromatin environment and cis-regulatory sequences could regulate gene expression noise. Thus, in the context of these previous studies, our work is the first systematic study that deconvolves the effects of the promoter sequence from the chromatin environment in regulating gene expression noise.

Also, while large-scale studies in *S. cerevisiae* have investigated how gene expression noise varies with mean expression (Bar-Even *et al*, 2006), the Poissonian scaling observed between CV and mean expression in these studies arises from contribution of both cis-regulatory sequences and the chromatin environment. Thus, from these studies it is not clear how the chromatin environment regulates gene expression noise, independent of cis-regulatory

sequences. Furthermore, while several transcriptional regulatory mechanisms are conserved from yeast to mammals, additional complexity in the mammalian genome (Rando & Chang, 2009; Court *et al*, 2011) may limit extrapolating results from yeast to mammalian systems. Thus, the limitations of these previous studies highlight the importance of our work, in which we systematically study the influence of only one variable, the chromatin environment, on gene expression noise. Finally, in addition to the differences noted above that might contribute to different results among previous studies, the use of different model organisms and differences in measurement techniques may also contribute to some of the differences in results.

The reviewer's suggestion of using live imaging to estimate burst size and burst frequency would provide additional validation of the kinetic parameters that are estimated in this study from the steady-state mRNA distributions across clones. That said, since the two-state model is ergodic, we would expect the results from live-imaging to be consistent with our results. We thank the reviewer for this suggestion, which we look forward to pursuing as follow-up experiments in future studies.

> *2. Integration of HIV genome (and therefore the authors reporters) is more likely to occur in relatively open chromatin, therefore full range of different chromatin contexts are unlikely to have been sampled in the present set of clones. I think there are studies related to this issue in HIV literature perhaps the authors should point to that caveat.*

We thank the reviewer for mentioning this. While the reviewer correctly points out that HIV *preferentially* integrates within actively transcribing genes and activating chromatin marks (Schroder, 2002; Lewinski *et al*, 2005; Wang *et al*, 2007), it has also been found to integrate at a lower frequency (up to ~5%) within genomic locations associated with gene deserts, methylated CpG regions, and repressive chromatin marks such as H3K27me3 (Lewinski *et al*, 2005; Wang *et al*, 2007). Thus, while our large set of 418 (=227+191) single-integration clones biases integration within euchromatic regions of the genome, we would also sample repressive genomic loci. We have now mentioned this caveat within the main text:

"While we isolate clones from the entire range of mean expressions, preferential integration of HIV into actively transcribing genes and activating chromatin marks potentially results in more clones being integrated within such regions than within gene deserts, methylated CpG regions, and repressive chromatin marks (Schroder, 2002; Lewinski *et al*, 2005; Wang *et al*, 2007)."

> *3. The description of figure 4E was confusing. The pairs of histograms are presumably from clone pairs that have the same mean but different noise. This should be clearly indicated both in the legend and in the relevant text. It would be cleared if they dropped prefix LGM2 from the clone names in this figure as they did in figure 6 B-E. Also, are the same set of clones being described in the two figures?*

We thank for the reviewer for pointing this out. We have now removed the prefix LGM2 from Figure 4E. We have also included the following text in the figure legend:

"mRNA copy number distributions are depicted as paired density histograms for six representative pairs of high noise (pair left, light gray) and low noise (pair right, dark gray). Each pair represents clones with similar mean expression but different CV. Experimentally determined histograms are shown overlaid with the probability density functions (red curve) evaluated using best-fit model parameters for each clone."

Figure 4E and Figure 6B-E both use the same set of clones.

> *4. A basic prediction is that clones with more accessible promoter regions will have higher level of expression. With that in mind, shouldn't a graph of levels of mean expression with the accessibility be provided for all clones? Closest figure to that is S18 but it does not directly address that issue.*

The hypothesis that more accessible promoter regions have higher levels of expression has been shown once transcription factors are expressed at high levels. Several groups, and previous work from our group, has shown that in the presence of cellular and viral transcription factors, chromatin accessibility serves to set the threshold of the levels of transcription factor required to initiate strong gene expression (Lam *et al*, 2008; Miller-Jensen *et al*, 2012). However, under basal conditions where activating transcription factors are absent or present at very low levels (as is the case in this study with low levels of cellular transcription factors required to initiate gene expression from the HIV promoter, such as NF-κB and Sp1 or viral transcription factors such as Tat that are absent in this system), gene expression is low and not strongly correlated to chromatin accessibility. For example, it has been previously shown that chromatin accessibility under basal conditions primarily determines the binding of transcription factors under activated conditions (John *et al*, 2011). Thus, under basal conditions, chromatin accessibility mainly determines and primes transcription factor binding to its regulatory sequences, with gene expression subsequently determined by the levels of transcription factors. In accordance with this, we find that under basal conditions (where levels of activating transcription factors are low) chromatin accessibility for the different clones presented in this work is only weakly correlated to mean gene expression. Since we found that this was not directly related to the main results presented in this manuscript, we did not include it in the Supplementary Figures. However, below we show this correlation for the reviewer's convenience.

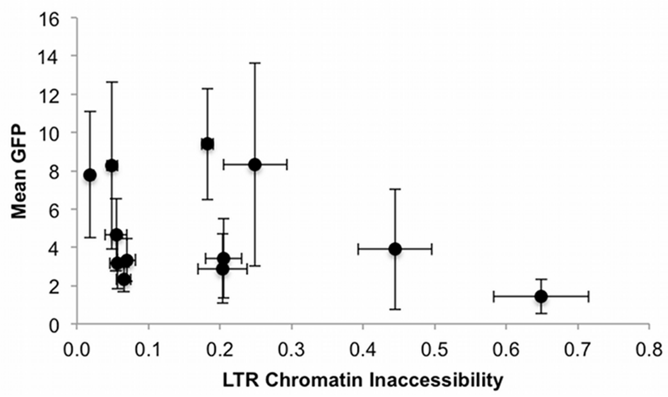

5. What is author's view on how the same promoter ends up with different nucleosome distributions depending on the chromatin context.

While the HIV promoter has been shown to contain well-positioned nucleosomes (Verdin *et al*, 1993; Pereira *et al*, 2000), the chromatin environment at the site of integration potentially plays a critical role in determining the extent of viral DNA compaction and the resulting variability in chromatin accessibility across integration sites. For example, DNA methylation within the HIV promoter at certain integration sites potentially recruits repressive factors that alter chromatin accessibility and gene expression (Kauder *et al*, 2009; Blazkova *et al*, 2009). Furthermore, differences in histone post-translational modifications at different integration sites potentially recruit different nucleosome remodeling complexes that influence genome compaction and chromatin accessibility (Rafati *et al*, 2011; Jiang & Pugh, 2009; Bell *et al*, 2011). Finally, differences in higher order chromatin structure and heterochromatin spreading (such as through the action of H3K9 methylation dependent recruitment of heterochromatin protein 1 (HP1)) results in less accessible chromatin at certain integration positions than others (Bell *et al*, 2011).

Reviewer 2

Dey and colleagues report an interesting experiment: a lentiviral-based reporter vector driving GFP was integrated in different (random and uncharacterized) positions in the genome. 227 clones were selected, and their single-cell distribution of GFP fluorescence was measured to define the mean expression and expression noise characterizing each of the clones. Out of those, 25 clones were selected for measuring the transcription levels using smFISH. This resulted in two main findings: (1) expression noise (CV) is uncorrelated with mean expression, (2) when comparing (six) clone pairs of similar expression, higher noise was associated with repressed chromatin (measured by DNase I sensitivity).

This experiment is interesting and presents a substantial amount of high-quality work. From result (1), the authors conclude that the noise does not result from a simple Poisson process. They fit the data to a two-state model of gene expression, and from this derive the burst size and burst frequency characterizing each clone. From that, they concluded that noise depends only on burst frequency, while mean expression depends (mostly) on burst size. This differs from previous results in mammalian cells, and also departs from results in model organisms including yeast and bacteria.

We thank the reviewer for considering our results interesting and for finding our data of high quality.

1. I see a potential problem with the analysis in that the authors did not consider the possibility of external noise (noise that results from variability upstream to the promoter, e.g. cell size, amount of ribosomes, transcription factors etc.). This extrinsic noise is not correlated with expression and will therefore provide the same lack of correlation between noise and mean expression, as reported here. In fact, also in yeast and bacteria, where the scaling of noise with expression is well established, a regime of independency exists for high expression

where extrinsic noise dominates. This is discussed in very many papers. So one possible interpretation of the lack of correlation is that extrinsic noise is very high, or we are in this regime where extrinsic noise dominates. The authors should include this possibility in their model and make sure that it cannot explain their data. (In this context, error bars are missing from figure 2 but are in fact quite essential - in particular the [likely] error in estimating noise. Such error-bars are typically generated by re-growing the same clones several times and comparing the distributions; likeliness of different models should be compared relative to those experimental errors).

We thank the reviewer for raising this point. In previous studies utilizing this experimental system, we systematically performed extensive controls to show that gene expression noise in our system is dominated by intrinsic noise and is not influenced by extrinsic sources of noise (Weinberger *et al*, 2005). Specifically, extensive controls were performed to show that extrinsic sources such as cell cycle, cell size, aneuploidy, and viral transcription factors do not influence gene expression noise in our system (Weinberger *et al*, 2005). Furthermore, other studies in mammalian systems have used two-color reporter systems to show that gene expression noise is dominated by intrinsic sources (Raj *et al*, 2006).

In this study, we have again considered the influence of extrinsic sources and found that it does not impact the results presented in this study. For example, we found that cell size (measured as forward scatter in flow cytometry) is not correlated to GFP fluorescence (slope is not significantly different from zero) for all the clones (Supplementary Figure 2). Furthermore, after applying a data-driven gating strategy that samples a small region of forward and side scatter space, we found no linear or other monotonic correlation between forward scatter and GFP expression (Supplementary Figure 3). These findings indicate that cell size does not influence the results presented in our study. Furthermore, we also analyzed if cell size potentially influences the relation between gene expression noise (CV) and mean expression. By choosing either a wide gate of forward and side scatter of live cells or a small gate centered on the density mode of forward and side scatter, we found that the observed independence of gene expression noise (CV) and mean expression (R^2 ~ 0) does not change (Supplementary Figure 4A). Finally, by changing the gate size centered around the density mode to capture between 10% to 90% of the cells, we found the slope of the variance in gene expression *vs.* mean expression best-fit line did not change (Supplementary Figure 4C). Taken together, and consistent with our prior work, these results strongly suggest that cell size does not influence the results presented in this study.

Next, we would like to note that possible differences in the amount of ribosomes between single cells do not seem to influence the results. Since we directly detect single mRNA molecules using FISH, the observed correlation between CV and mean mRNA expression (or variance and mean mRNA expression) is not statistically different from the same correlations at the protein (GFP) level. This suggests that the independent control of expression mean and noise is not influenced by the amount of ribosomes.

Finally, we make a theoretical argument that has previously been discussed in detail (Skupsky *et al*, 2010), for why other extrinsic factors, such as transcription factors, potentially do not influence the results of our study. First, the range of noise (CV) observed with the HIV promoter for different

clones used in this study (~0.25-0.8) is larger than that observed in other large-scale studies involving several cellular promoters (~0.1-0.3) (Sigal *et al*, 2006; Newman & Weissman, 2006). Next, the noise observed in the mRNA distributions (CV_t) are typically higher than those observed in the protein (GFP) distributions (CV_p) (Figures 2B and 5C). Further, assuming a bursting regime, the noise in promoter state transitions can be given by (Skupsky *et al*, 2010):

$$CV^2_{promoter} = \left(\frac{b}{b+1}\right)\left(\frac{k_r}{k_{t-}}\right) CV_t^2$$

In our dataset, we observe burst sizes (*b)* greater than 1 (Figure 4A), and thus the first term in the equation is close to 1. The second term is much greater than 1 for all the clones. This implies that the noise introduced from the promoter state transitions ($CV_{promoter}$) is greater than the noise in transcript counts (CV_t), which in turn is greater than noise in protein distributions (CV_p). In general, in a cascade of stochastic processes, for noise at a particular step to make a significant contribution and propagate to the next step, the upstream step needs to be noisier than the downstream step. But since it appears that the observed noise in protein (GFP) distributions from the HIV promoter is possibly higher than endogenous genes, it is unlikely that noise in transcription factors (or other cellular proteins) could influence and propagate through the HIV promoter to significantly affect the observed mRNA and GFP distributions.

Thus, based on rigorous previous experimental validation in our group (Weinberger *et al*, 2005), along with the analysis presented here (Supplementary Figures 1,2,3 and 4) and direct measurement of single mRNA molecules, the observed noise in gene expression should primarily be governed by intrinsic processes.

We thank the reviewer for pointing out that the error bars in Figure 2 are missing. We have now included error bars for this figure. For a detailed discussion on error-bars, please see the section on "Additional Remarks (#1)" from Reviewer 2 below. Briefly, we would like to mention that the data shown in this manuscript are at steady-state. We performed a time-course experiment for 5 days to show that the mean GFP expression and noise (CV) does not change for the clones (Supplementary Figure 8). Furthermore, we isolated an independent set of 191 clones (in addition to data from the 227 clones presented in the main manuscript) to show that the correlation between GFP variance *vs.* mean expression or GFP CV *vs.* mean expression was unchanged (Supplementary Figure 6). These results show that the GFP distributions were broadly unchanged and had achieved steady state. Finally, the shaded areas around the regression lines represent 95% confidence intervals, which show that the GFP variance can be related to mean expression with high confidence by $\sigma^2 \propto \mu^{2.1\pm0.1}$ (p < 0.001), thereby implying that the CV is uncorrelated to mean expression (R^2 = 0.013). These results and correlations were independently verified in the technical replicate of 191 clones (Supplementary Figure 6).

2. With regards to the two-state models: it is nice that that the model can fit the disrtibutions, but I wonder how restrictive this fit is, since there are enough parameters one can adjust. A more intuitive explanation of the model will also help. A well appreciated result is that in the two-state model, noise^2 is given by 1/[burst-frequency], while mean expression is given by

[burst_size burst_frequency]. This should be mentioned and explained. In this context, the work of Johan Paullson who played a central role in setting up this analysis should be cited. The work of Nir Friedman et al (PRL) is also highly relevant and should be cited.*

When considering the result mentioned above, the fact that noise correlates with burst frequency may be a the result of the fitting procedure, so I'm not sure its biological interpretation is justified. Mean expression is explained by the combination of both burst size and burst frequency, and the fact that, overall, expression changes more than noise, will result in a dominating effect of burst size, as observed. Again, one possibility that the author should consider is that this is the mere result of fitting, and not the only biological interpretation. A more rigorous evaluation of this point is therefore needed, in particular considering the possibility of extrinsic noise, as I suggested above, but perhaps additional possibilities.

In fitting the two-state model to the mRNA distributions, we have fit two parameters, as described in detail in the Supplementary section "Model Fitting" (Supplementary Figure 14). A simpler model with a single parameter would be a Poisson model in which transcripts are produced at a constant rate. Since such a model does not fit the mRNA distributions well (Figure 2), the two-state model where we fit two parameters is one of the simplest models (that has been frequently used in literature (Skupsky *et al*, 2010; Raj *et al*, 2006; Raser & O'Shea, 2004; Dar *et al*, 2012)) that parsimoniously explains the observed mRNA distributions. To convince the reviewer further, we have performed additional sensitivity analysis to show that the maximum likelihood fits are restrictive and explain the data well.

To perform sensitivity analysis, we compared the experimental mRNA FISH distributions for each clone to theoretical distributions (Supplementary Figure 14B) for different parameter values of burst frequency and burst size. We used the Kullback-Leibler (KL) divergence to quantify how closely the theoretical distribution with a particular set of parameters approximates the experimental distribution (The Kullback-Leibler divergence is a information theoretic measure that quantifies the extra bits of information required for the theoretical distribution, with two parameters in this case, to approximate the experimental distribution). Smaller KL divergences imply that a particular set of parameters in the theoretical distribution approximates the experimental distribution more closely. For all the clones we observed that the MLE best-fits parameters gave the lowest KL divergences with these divergences increasing in all directions in the 2-D parameter space (Supplementary Figure 14D). The heatmap shows KL divergences for different parameter values (centered around the MLE best-fit parameter values) with the coloring pattern serving as an guide to the eye. A heatmap for one clone is included below and similar sensitivity analysis for all the other clones are now included in Supplementary Figure 14D.

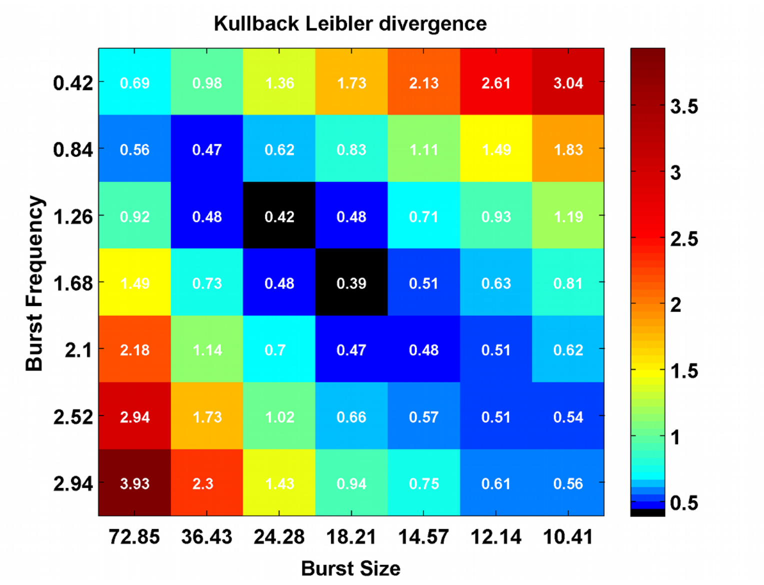

Supplementary Figure 14D

We thank the reviewer for pointing out some well-known results that can be derived from the two-state model. However, we would like to point out that these results only arise when assuming the bursting regime ($k_r >> k_{t-}$ and $k_r >> k_a$) in the two-state model. In estimating model parameters, we do not use the moments but use the entire analytically derived mRNA distribution in the two-state model to estimate these parameters. Thus, the reviewer's concerns that the results obtained in this work could potentially be explained by the results mentioned by him/her are not necessarily the case since the results are obtained by solving and fitting the data to the entire analytical solution (Supplementary Figure 14B). In fitting the data, we make no assumptions of the "regime" in which the parameters lie. Therefore, *a priori*, using the two-state model does not imply observing the correlations found in our data. For example, noise (CV) is a function of both burst size and burst frequency in the complete two-state model without assuming the bursting regime. The analytical solution for the mRNA distribution (Supplementary Figure 14B) can be used to estimate the first two moments of the distribution (Peccoud & Ycart, 1995):

Mean:

$$\mu = \left(\frac{k_a}{k_a + k_r}\right)\left(\frac{k_{t+}}{k_{t-}}\right)$$

Variance:

$$\sigma^2 = \left(\frac{k_a}{k_a + k_r}\right)\left(\frac{k_{t+}}{k_{t-}}\right) + \left[\frac{k_a k_r}{(k_a + k_r)^2}\right]\left[\frac{{k_{t+}}^2}{k_{t-}(k_a + k_r + k_{t-})}\right]$$

Thus, CV is given by:

$$CV^2 = \frac{\sigma^2}{\mu^2} = \left(\frac{k_{t-}}{k_a}\right)\left\{\frac{k_a + k_r}{k_{t+}} + \frac{k_r}{k_a + k_r + k_{t-}}\right\}$$

In the bursting regime (k_r >> k_{t-} and k_r >> k_a), these equations reduce to:

$$\mu = \bar{k}_a b$$
$$\sigma^2 = \bar{k}_a b(b+1)$$
$$\sigma^2 \approx \bar{k}_a b^2 \quad \text{..... for } b>>1$$

Thus,

$$CV^2 = \frac{\sigma^2}{\mu^2} = \frac{1}{\bar{k}_a}$$

where $\bar{k}_a$ is the normalized burst frequency and b is the burst size.

Thus, the results presented in this work are derived from the entire mRNA distribution where the first two moments of the distribution (mean and variance) are a function of both burst frequency and burst size and no assumptions about any particular regime have been made. Therefore, the correlations observed in our dataset between mean expression and burst size or gene expression noise (CV) and burst frequency are not dependent on the equations for the first two moments of the distribution assuming a bursting regime.

We have now included and explained the above equations for the first two moments of the distribution (with or without assuming the bursting regime) in Supplementary Information (Supplementary Figure 14C). For a more intuitive explanation of the two main parameters of the model we have now included the following text in the legend of Supplementary Figure 14:

"The burst frequency describes the rate at which the promoter switches from an inactive (or Off state from which no transcripts are produced) to an active state (or On state from which transcripts are produced at a rate of k_{t+}). The transition from the Off to On state can be thought of as a transition from a repressed promoter to an active promoter state where the transcription machinery binds and RNA polymerase initiates the production of transcripts. The burst size describes the average number of transcripts that are produced every time the promoter transitions to the On state."

We thank the reviewer for mentioning the work of Johan Paulsson and Nir Friedman. In addition to Johan Paulsson, we have now cited the work of Nir Friedman in developing theoretical frameworks for analyzing gene expression noise (Paulsson, 2004; Friedman *et al*, 2006). Finally, for a detailed discussion on why extrinsic sources of noise do not influence the results presented in this study, please see Response to Reviewer 2 (#1).

Additional remarks:

1. How were the error-bars measured for mRNA expression/noise? do they reflect measurements on different days (should be)

All measurements of GFP expression and single mRNA molecules were made at steady-state. This was verified using a time course experiment where we observed that GFP expression of the

clones did not change over the course of 5 days (Supplementary Figure 8). We found that the slope of the GFP variance *vs.* mean expression best-fit line did not change over time (Supplementary Figure 8A,B). Furthermore, the mean expression and CV of individual clones did not change over time (Supplementary Figure 8C,D), suggesting that the GFP distributions were broadly unchanged over the time course experiment. Finally, to ensure that the results presented in this manuscript were robust and free of technical variabilities, we also repeated the entire experimental setup to isolate 191 clones in addition to the data presented from the 227 clones presented in the main manuscript and found the same scaling between noise (CV) and mean expression. The error-bars presented in the manuscript are based on bootstrapping the data as explained under the subsection "Statistical Analysis" in Materials and Methods.

Furthermore, single molecule mRNA FISH results were also unchanged over time. Technical replicates of mRNA FISH data performed on different days are shown below for one clone. The data shows minimal technical variability in single-molecule mRNA FISH results.

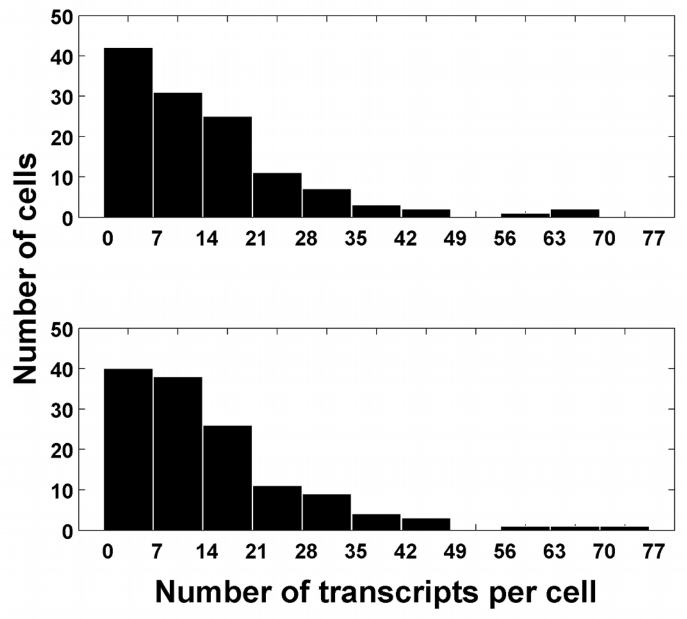

2. The effect if chromatin on burst size was discussed quite extensively in the yeast model where promoter structure was shown to strongly correlate with the normalized-noise, which, by definition, is burst size. This should be mentioned. (e.g. hornung et al. Mol Cell 2012 ; Tirosh and Barkai Genome Research 208 or other related papers)

We have now included these references (Tirosh & Barkai, 2008; Hornung *et al*, 2012) together with several additional studies that we had discussed in the main manuscript which studied the influence of chromatin in regulating gene expression dynamics (Miller-Jensen *et al*, 2012; Mao *et al*, 2010; Lam *et al*, 2008). Finally, as discussed above (please see Response to Reviewer 2 (#2)), in arriving at our conclusions we made no *a priori* assumptions about how burst size and burst frequency correlate with gene expression noise.

3. The discussion is too much centered on comparison with previous studies. This will greatly interest the experts, but less so the general reader. I would suggest the authors to (perhaps more briefly) discuss the disagreement in the results/ supplementary and devote the discussion to the more general implications of their results.

We thank the reviewer for this suggestion. We have now discussed in greater detail how the chromatin environment at the promoter may play an important in fine-tuning transcription to produce different cellular phenotypes. In the context of HIV, this may be an important factor that could bias the provirus towards a lytic or latent outcome. We have now included the following text in the Discussion section of the main manuscript:

"This work shows that in mammalian systems nucleosomes and chromatin density around the TSS may also be important in fine-tuning transcription. Such a strategy where the chromatin environment regulates gene expression noise could be an important mechanism to generate different cellular phenotypes from isogenic populations in a manner that can confer increased evolutionary fitness. For example, in simple eukaryotes, such phenotypic switching has been shown to confer increased survival fitness (Acar *et al*, 2008). Similarly, such cell-to-cell heterogeneity in cancer populations may be an important mechanism that contributes to different drug sensitivities and drug-tolerant states (Sharma *et al*, 2010; Singh *et al*, 2010b; Spencer *et al*, 2009; Cohen *et al*, 2008). Finally, phenotypic heterogeneity may be an important contributor to producing low frequency latent HIV-1 infections that remain one of the main obstacles to completely eliminating the virus from a patient (Weinberger *et al*, 2005; Ho *et al*, 2013). Thus, understanding the contribution of the chromatin environment in regulating gene expression noise and the resulting phenotypic heterogeneity may be important for understanding the design principles governing evolution and for developing better treatments in a variety of diseased states."

Reviewer 3

Dey et al explore the causes that explain variations in noise due to chromosomal position effect on a lentiviral gene expression cassette. In particular, they find that nucleosome accessibility is decreased between clones displaying high noise, and it is the on-rate in the two-state promoter model that mediates this effect. While chromosome position affects burst size to modulate mean expression levels (as already indicated in their earlier study), burst frequency explains those variations in noise that deviate from the constant trend line of CV values as the mean is varied.
While the main findings on nucleosome accessibility and corollaries are interesting it is often difficult to find the data that would permit to assess the consistency of the observations and to compare to other studies.

We thank the reviewer for finding our results interesting, including measurements where we show that chromosomal locations associated with reduced chromatin accessibility exhibit increased

gene expression noise which is explained entirely by reduced burst frequencies. In the revised manuscript, we have included the raw data and fit parameters as an additional supplementary table as detailed below.

I suggest the following changes before publication:
1. Relevant range of parameter values. It is very difficult to collect the parameter values that have been fitted for the clones.
A. Figure S13: Negative values for mRNA decay rates are presented. Even if regression is log-linear, which is quite common to fit decay rates, the values for all biochemical rates should be presented in original (non-transformed) positive numbers.
B. Figures 5, 7: On rate is normalized to RNA decay rate. Quite surprisingly, absolute on rates are never presented even though they can be easily calculated. There should be a (supplementary) table provided where all the fitted parameters (in Figure S14) should be presented for a set of clones (e.g. for those shown in Figure 7). These values will permit verifying predictions by time-series measurements. If the absolute values of off-rates and transcription rates cannot be assessed independently then their ratios (burst size) should be indicated.

In Supplementary Figure 13, we have now presented the mRNA decay rates as positive numbers along with the half-lives of these species.

The use of normalized On rates is a natural consequence of parameter estimation using the analytical mRNA distribution (Supplementary Figure 14) and has been used in previous studies (Skupsky *et al*, 2010). Estimating the absolute On rate (=Normalized On rate*Transcript degradation rate) is trivial since we have experimentally measured the transcript degradation rate. As the reviewer suggested, we have now provided the absolute On rate and the burst size together with other experimentally measured values (for clones shown in Figure 7) in Supplementary Table 1.

Clone	GFP (Mean)	GFP (Variance)	GFP (CV)	Number of Cells (mRNA FISH)	mRNA (Mean)	mRNA (Variance)	mRNA (CV)	Absolute Burst Frequency k_a (hour^{-1})	Burst Size	Chromatin Inaccessibility (Nuc1)	Chromatin Inaccessibility (HSS)	Chromatin Inaccessibility (Nuc0)	Chromatin Inaccessibility (Large Region around TSS)
CA1	9.398	8.432	0.309	1027	17.430	111.004	0.604	0.856	7.667	0.773	0.491	0.596	0.182
CA2	3.420	4.255	0.603	1058	7.126	25.993	0.715	1.021	2.472	1.092	0.966	0.974	0.205
CD2	4.655	3.583	0.407	482	6.041	30.634	0.916	0.406	5.228	0.776	0.547	0.626	0.055
EC5	3.899	9.890	0.807	816	7.217	41.613	0.894	0.466	5.488	1.023	1.126	1.268	0.444
FA1	3.179	1.804	0.422	1050	4.635	13.580	0.795	0.868	1.865	0.643	0.425	0.609	0.056
FA6	3.330	1.240	0.334	1061	11.425	67.679	0.720	0.721	5.758	0.686	0.565	0.780	0.069
FC5	2.323	0.431	0.283	782	7.084	29.483	0.766	0.691	3.633	0.730	0.478	0.760	0.065
GA2	2.881	3.307	0.631	679	6.454	37.508	0.949	0.375	6.073	1.016	0.885	0.981	0.203
GD1	8.318	28.012	0.636	513	26.012	353.836	0.723	0.570	18.213	0.969	1.072	0.987	0.249
HA4	1.421	0.806	0.632	469	5.079	33.701	1.143	0.236	7.506	0.925	1.163	1.269	0.649
IB4	8.262	18.933	0.527	372	18.543	135.074	0.627	0.864	8.152	0.641	0.778	0.989	0.049
IC4	7.778	10.858	0.424	435	19.041	104.307	0.536	1.088	6.664	0.394	0.350	0.543	0.018

Supplementary Table 1

2. Relevance of the study. Both in the introduction and discussion, the results are compared to studies that explore genome wide trends of different genes (or promoters). On the other hand, the promoter is identical in the different clones in this study and only chromosomal position is varied. While it is reasonable to compare these two types of studies, there is no need to

expect consistency between the trends found in these studies. Furthermore, expression driven by different promoters often spans a range of more than three orders of magnitudes. On the other hand, the majority of the clones in this study spans a range of one order of magnitude.

We thank the reviewer for mentioning this. We agree that the use of different model organisms, cell lines, promoters, and chromosomal locations could all influence the final results and trends observed in previous studies. We have now mentioned this in the Discussion section:

"While differences in results between these studies could arise from some of the reasons mentioned above, the use of different model organisms, cell types, promoters, and chromosomal locations could also influence the final conclusions of these studies. Taken together, these recent studies and our work suggest that both promoter architecture and the local chromatin environment may combine to yield the observed expression distributions and inferred transcription dynamics."

Finally, we agree that while endogenous genes typically span three orders of magnitude in expression levels, and the data presented in our study spans one to two orders of magnitude (Figure 1A), this work provides the first systematic study that deconvolves the effect of the promoter to understand how the chromatin environment regulates gene expression noise. We hope this study motivates other groups and ours to perform more extensive genome-wide studies that analyze other promoters to see if this provides additional novel insights into gene regulation.

3. There is no any discussion of potential mechanisms that could account for the central message of the paper, the orthogonal control of expression mean and variance, even though there are compatible mechanisms known. Epigenetic modification is one of the major mechanisms known to modulate nucleosome occupancy as a function of chromosomal position since such modifications can spread along the chromosome. For example, epigenetic silencing in yeast can result in orthogonal control of expression mean and variance reactions (Kelemen et al (2010) PLOS Biology, Spatial epigenetic control of mono- and bistable gene expression.) Bimodal expression (and hence large noise) can arise when epigenetic modifiers are recruited on both ends of the gene (bilateral recruitment). Conversely, noise is smaller when the modifiers are recruited only on one end of the gene (unilateral recruitment), even though the mean of expression level can be reduced massively by this form of silencing. Silencing in yeast can also decreases nucleosome accessibility.

As the reviewer suggests, several mechanisms could potentially regulate the independent control of expression mean and noise. In this study we show that the nucleosome positioned at the transcription start site (Nuc-1) is the most important regulator of burst frequency and gene expression noise. It is plausible that the transcription machinery is blocked by Nuc-1 in the Off state, transcription initiates only when Nuc-1 transiently disassembles, and this binding and unbinding of the nucleosome regulates the burst frequency and gene expression noise. Independent control of the burst size and therefore mean expression could arise from the availability or recruitment of transcription factors or chromatin remodeling factors to particular genomic locations that are dependent on chromatin modifications at that location. In addition to chromatin accessibility, chromatin modifications and transcription factor recruitment, other factors

such as DNA methylation, polymerase pausing and release could potentially result in the orthogonal control of expression mean and noise. In the Discussion section, we have now discussed this briefly:

"Several biochemical processes could result in the independent control of expression mean and noise observed in this study. While we show that the nucleosome positioned at the transcription start site plays the most important role in regulating gene expression noise, variability in chromatin modifications and transcription factor recruitment at different genomic locations may influence polymerase processivity or burst size, thereby independently regulating mean gene expression. Finally, the interplay between these processes, together with other mechanisms of gene regulation such as DNA methylation or polymerase pausing, could potentially result in the independent control of expression mean and noise."

The authors claim that the telegraph model is "abstract". It would be useful to provide a couple of statements how the interpretation of data will change if the transition between the two states is not a simple linear process.

While the single-cell mRNA distributions presented in this study could be fit to more complex gene expression models (such as those in which the transition between the Off and On states are governed by non-linear processes), we find that the two-state model is the most parsimonious model that explains the data well. Since the parameters in these models represent lumped biochemical processes, it is unclear if more complex models with additional fit parameters would yield any new biological insights.

Minor comments:
A. CV values should be shown consistently. Since CV values are already normalized they should be shown in linear scale (just as in Figure 5). On the other hand, the logarithms of CV values are shown in other figures.

The reason the CV is shown on a log scale in all figures (except Figure 5) is that in the other figures the CV is plotted against mean expression. Since we were interested in understanding if stochastic gene expression in our system is governed by a Poissonian process, where $CV \propto 1/\sqrt{\mu}$, a plot on a log-log scale allows simple visual inspection and fitting to observe if the slope of the line in log-log space deviates from -1/2.

B. Those clones to highlight/label in Figure 2 that have been selected for the nucleosome experiments (Figure 6, 7).

The clones on which we performed chromatin accessibility assays and single-molecule mRNA FISH have now been highlighted in Figure 2.

References

Acar M, Mettetal JT & van Oudenaarden A (2008) Stochastic switching as a survival strategy in fluctuating environments. *Nature Genetics* **40:** 471–475

Bar-Even A, Paulsson J, Maheshri N, Carmi M, O'Shea E, Pilpel Y & Barkai N (2006) Noise in protein expression scales with natural protein abundance. *Nature Genetics* **38:** 636–643

Bell O, Tiwari VK, Thomä NH & Schübeler D (2011) Determinants and dynamics of genome accessibility. *Nat Rev Genet* **12:** 554–564

Blazkova J, Trejbalova K, Gondois-Rey F, Halfon P, Philibert P, Guiguen A, Verdin E, Olive D, Van Lint C, Hejnar J & Hirsch I (2009) CpG methylation controls reactivation of HIV from latency. *PLoS Pathog.* **5:** e1000554

Cohen AA, Geva-Zatorsky N, Eden E, Frenkel-Morgenstern M, Issaeva I, Sigal A, Milo R, Cohen-Saidon C, Liron Y, Kam Z, Cohen L, Danon T, Perzov N & Alon U (2008) Dynamic proteomics of individual cancer cells in response to a drug. *Science* **322:** 1511–1516

Court F, Miro J, Braem C, Lelay-Taha M-N, Brisebarre A, Atger F, Gostan T, Weber M, Cathala G & Forné T (2011) Modulated contact frequencies at gene-rich loci support a statistical helix model for mammalian chromatin organization. *Genome Biology* **12:** R42

Dar RD, Razooky BS, Singh A, Trimeloni TV, McCollum JM, Cox CD, Simpson ML & Weinberger LS (2012) Transcriptional burst frequency and burst size are equally modulated across the human genome. *Proc. Natl. Acad. Sci. U.S.A.* **109:** 17454–17459

Friedman N, Cai L & Xie XS (2006) Linking stochastic dynamics to population distribution: an analytical framework of gene expression. *Phys. Rev. Lett.* **97:** 168302

Ho Y-C, Shan L, Hosmane NN, Wang J, Laskey SB, Rosenbloom DIS, Lai J, Blankson JN, Siliciano JD & Siliciano RF (2013) Replication-competent noninduced proviruses in the latent reservoir increase barrier to HIV-1 cure. *Cell* **155:** 540–551

Hornung G, Oren M & Barkai N (2012) Nucleosome organization affects the sensitivity of gene expression to promoter mutations. *Mol. Cell* **46:** 362–368

Jiang C & Pugh BF (2009) Nucleosome positioning and gene regulation: advances through genomics. *Nat Rev Genet* **10:** 161–172

John S, Sabo PJ, Thurman RE, Sung M-H, Biddie SC, Johnson TA, Hager GL & Stamatoyannopoulos JA (2011) Chromatin accessibility pre-determines glucocorticoid receptor binding patterns. *Nature Genetics* **43:** 264–268

Kauder SE, Bosque A, Lindqvist A, Planelles V & Verdin E (2009) Epigenetic regulation of HIV-1 latency by cytosine methylation. *PLoS Pathog.* **5:** e1000495

Lam FH, Steger DJ & O'Shea EK (2008) Chromatin decouples promoter threshold from dynamic range. *Nature* **453:** 246–250

Lewinski MK, Bisgrove D, Shinn P, Chen H, Hoffmann C, Hannenhalli S, Verdin E, Berry CC, Ecker JR & Bushman FD (2005) Genome-wide analysis of chromosomal features repressing human immunodeficiency virus transcription. *J. Virol.* **79:** 6610–6619

Mao C, Brown CR, Falkovskaia E, Dong S, Hrabeta-Robinson E, Wenger L & Boeger H (2010) Quantitative analysis of the transcription control mechanism. *Mol. Syst. Biol.* **6:** 431

Miller-Jensen K, Dey SS, Pham N, Foley JE, Arkin AP & Schaffer DV (2012) Chromatin accessibility at the HIV LTR promoter sets a threshold for NF-κB mediated viral gene expression. *Integr Biol (Camb)* **4:** 661

Newman JRS & Weissman JS (2006) Systems biology: many things from one. *Nature* **444:** 561–562

Paulsson J (2004) Summing up the noise in gene networks. *Nature* **427:** 415–418

Peccoud J & Ycart B (1995) Markovian Modeling of Gene-Product Synthesis. *Theoretical Population Biology*

Pereira LA, Bentley K, Peeters A, Churchill MJ & Deacon NJ (2000) A compilation of cellular transcription factor interactions with the HIV-1 LTR promoter. *Nucleic Acids Research* **28:** 663–668

Rafati H, Parra M, Hakre S, Moshkin Y, Verdin E & Mahmoudi T (2011) Repressive LTR nucleosome positioning by the BAF complex is required for HIV latency. *PLoS Biol.* **9:** e1001206

Raj A, Peskin CS, Tranchina D, Vargas DY & Tyagi S (2006) Stochastic mRNA Synthesis in Mammalian Cells. *PLoS Biol.* **4:** e309

Rando OJ & Chang HY (2009) Genome-wide views of chromatin structure. *Annu. Rev. Biochem.* **78:** 245–271

Raser JM & O'Shea EK (2004) Control of stochasticity in eukaryotic gene expression. *Science* **304:** 1811–1814

Schroder ARW (2002) HIV-1 Integration in the Human Genome Favors Active Genes and Local Hotspots. **:** 1–9

Sharma SV, Lee DY, Li B, Quinlan MP, Takahashi F, Maheswaran S, McDermott U, Azizian N, Zou L, Fischbach MA, Wong K-K, Brandstetter K, Wittner B, Ramaswamy S, Classon M & Settleman J (2010) A chromatin-mediated reversible drug-tolerant state in cancer cell subpopulations. *Cell* **141:** 69–80

Sigal A, Milo R, Cohen A, Geva-Zatorsky N, Klein Y, Liron Y, Rosenfeld N, Danon T, Perzov N & Alon U (2006) Variability and memory of protein levels in human cells. *Nature* **444:** 643–646

Singh A, Razooky B, Cox CD, Simpson ML & Weinberger LS (2010a) Transcriptional bursting from the HIV-1 promoter is a significant source of stochastic noise in HIV-1 gene expression. *Biophys. J.* **98:** L32–4

Singh DK, Ku C-J, Wichaidit C, Steininger RJ, Wu LF & Altschuler SJ (2010b) Patterns of basal signaling heterogeneity can distinguish cellular populations with different drug sensitivities. *Mol. Syst. Biol.* **6:** 369

Skupsky R, Burnett JC, Foley JE, Schaffer DV & Arkin AP (2010) HIV promoter integration site primarily modulates transcriptional burst size rather than frequency. *PLoS Computational biology* **6:**

Spencer SL, Gaudet S, Albeck JG, Burke JM & Sorger PK (2009) Non-genetic origins of cell-to-cell variability in TRAIL-induced apoptosis. *Nature* **459:** 428–432

Suter DM, Molina N, Gatfield D, Schneider K, Schibler U & Naef F (2011) Mammalian genes are transcribed with widely different bursting kinetics. *Science* **332:** 472–474

Tirosh I & Barkai N (2008) Two strategies for gene regulation by promoter nucleosomes. *Genome Research* **18:** 1084–1091

Verdin E, Paras P Jr & Van Lint C (1993) Chromatin disruption in the promoter of human immunodeficiency virus type 1 during transcriptional activation. *EMBO J.*

Wang GP, Ciuffi A, Leipzig J, Berry CC & Bushman FD (2007) HIV integration site selection: analysis by massively parallel pyrosequencing reveals association with epigenetic modifications. *Genome Research* **17:** 1186–1194

Weinberger LS, Burnett JC, Toettcher JE, Arkin AP & Schaffer DV (2005) Stochastic Gene Expression in a Lentiviral Positive-Feedback Loop: HIV-1 Tat Fluctuations Drive Phenotypic Diversity. *Cell* **122:** 169–182

2nd Editorial Decision 04 March 2015

Thank you again for submitting your work to Molecular Systems Biology. We have now heard back from the referees who agreed to evaluate your manuscript. As you will see below, while reviewer #1 is satisfied with the modifications made and thinks that the study is now suitable for publication, reviewer #2 has some remaining concerns regarding the potential contribution of extrinsic noise. We have circulated the reports to all reviewers as part of our 'pre-decision cross-commenting' policy. During this process, reviewer #1, mentioned: "I think the author's response to the concern of reviewer #2 regarding extrinsic noise is reasonable and doing two color experiments that are rather arduous and have been done before (albeit in different contexts), is too much to ask for. However, the authors should discuss this issue in the light of forgoing discussion". As such, we think that further analyses are not mandatory for the acceptance of this work (of course we would encourage you to include them, in case they are already available) and we would like to ask you to include a brief discussion on this point.

Reviewer #1:

After carefully considering the authors responses to my concerns to those of other reviewers, I am satisfied. The paper makes exciting contribution in the field of noise in gene expression.

Reviewer #2:

In my previous reports I raised two concerns. The first (and major one) was dismissal of extrinsic noise as a possible explanation for the observed lack of correlation between noise and mean expression. The second was the missing error-bars and lack of biological repeat for measurements of individual clones.

The second concern was partially answered with the addition of error bars, but no independent repeats were performed to estimate day-to-day variability. Following a cell over time is very different from biological repeat. However, I am ok with the explanations given by the authors - although not optimal, the results are probably valid.

with regards to my main concern - possibility of external noise - here I must say I wasn't convinced by the authors' discussion. There are so many possibilities for extrinsic noise that this cannot be dismissed because of lack of correlation with FACS-based size measurements of based on a paper from over ten years ago. The decisive experiment is clear - the dual color approach. Alternatively, this possibility needs to be included in the model and discussed on equal footing with the burst-size dependent explanation.

Again, the problem in my mind is that i external noise is dominating this will completely change THE major conclusion of paper. for this reason, I don't see how it can be taken slightly and refuted based on arguments that are not direct.

2nd Revision - authors' response 25 March 2015

We would like to thank you and the referees for the positive feedback on our revised Orthogonal control of expression mean and variance by epigenetic features at different genomic loci. In this revised manuscript, we have addressed the remaining comments of the referees. Below we describe in detail all the changes included in the manuscript.

We would like to thank Reviewer 1 for finding this study ready for publication and an exciting contribution to the field of gene expression noise.

© European Molecular Biology Organization

We would like to thank Reviewer 2 for his/her constructive comments.
To address the reviewer's comment on performing independent repeats, we would like to point out that the lack of correlation between mean expression and CV in 227 clones was independently validated in a second experiment with 191 clones (Figure 2 and Supplementary Figure S6). Additionally, to ensure that there were no day-to-day variations and that the system had reached steady-state, we did a time course experiment to show that the GFP distributions were unchanged over time (Supplementary Figure S8).

To address the reviewer's comment on extrinsic noise influencing the results of the study, we have now discussed this in detail in the main manuscript and Supplementary information as suggested by you and by Reviewer 1 in the pre-decision cross-commenting section. In the main manuscript (pages 8-9), we have now discussed how cell size did not correlate with and influence the GFP distributions in this study (Supplementary Figures S2,S3 and S4). Furthermore, we have discussed previous experiments from our group (Weinberger et al, 2005) showing that extrinsic sources of noise, such as cell cycle and/or DNA content, do not influence gene expression noise in our system.

Finally, as mentioned by Reviewer 1, we have now cited a previous study in a mammalian system that used a two-color reporter system to show that gene expression noise is dominated by intrinsic sources (Raj et al, 2006). Extending the discussion on extrinsic noise further (middle paragraph on page 10), we now mention that a similar lack of correlation between mean expression and CV on the mRNA level suggests that extrinsic sources of noise related to translation, such as the numbers of ribosomes, do not influence gene expression noise in our system. Finally, in the Supplementary information section (pages 3-4), we have now added a theoretical discussion on why gene expression noise in our system is potentially dominated by manuscript intrinsic sources compared to other sources of extrinsic noise, such as transcription factors.
